# Supplementary material for: Genetic hierarchy and temporal variegation in the clonal history of acute myeloid leukaemia
Source: Nat Commun. 2016 Aug 18;7:12475. doi: 10.1038/ncomms12475 (PMC4992157; doi:10.1038/ncomms12475)
Supplement: Supplementary Information — Supplementary Figures 1-9 and Supplementary Tables 1-14. [file ncomms12475-s1.pdf]

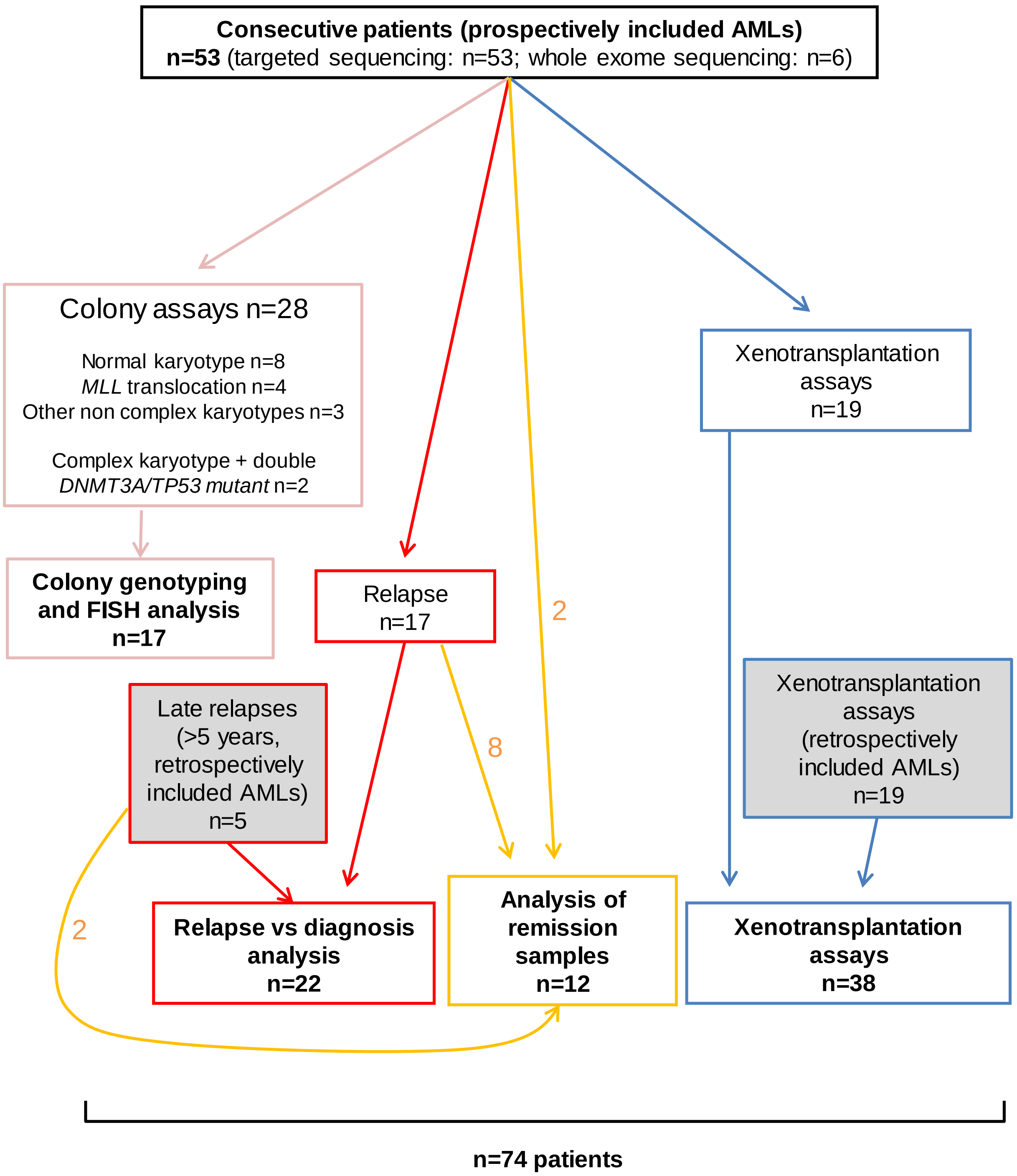

Supplementary Figure 1 | Flowchart of the study

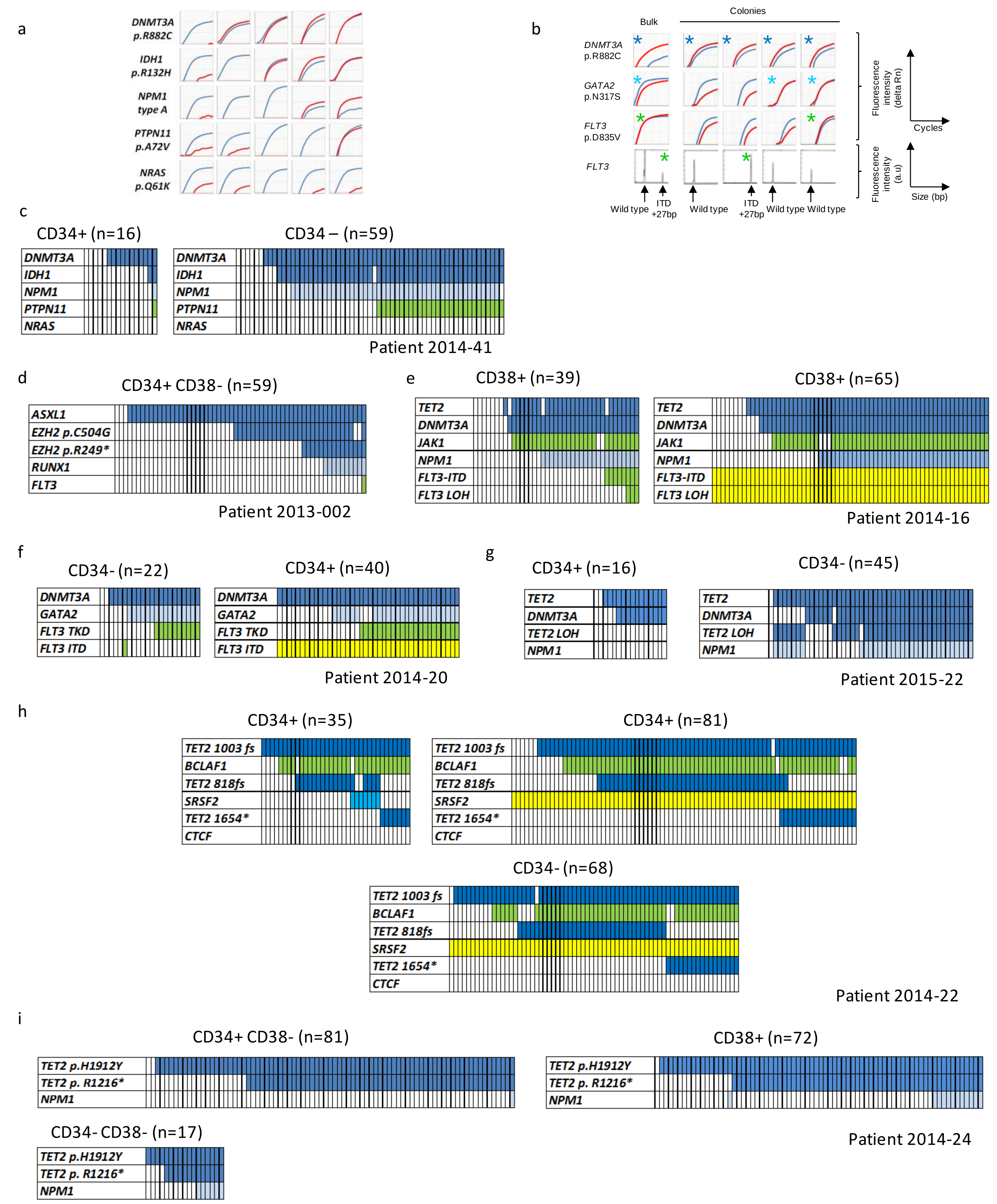

Supplementary Figure 2 | Summary of single cell derived colony assays in seven patients with normal karyotype. a, PCR amplification plots of allele-specific SNP (AS) assays in five colonies (colonies: columns; mutations: rows) from UPN2014-041 as in Fig.1a. **b,** Allele specific PCR amplification plots, as in Fig.1a, and traces of high resolution sizing of fluorescent PCR amplicons of *FLT3* in bulk AML and four colonies from patient UPN2014-020. **c, d, e, f, g, h, i,** Results of AS assays in 7 patients as in figure 1. For each patient, the cell phenotype before culture and the number of analysed colonies are indicated above the table. Yellow box means no data.

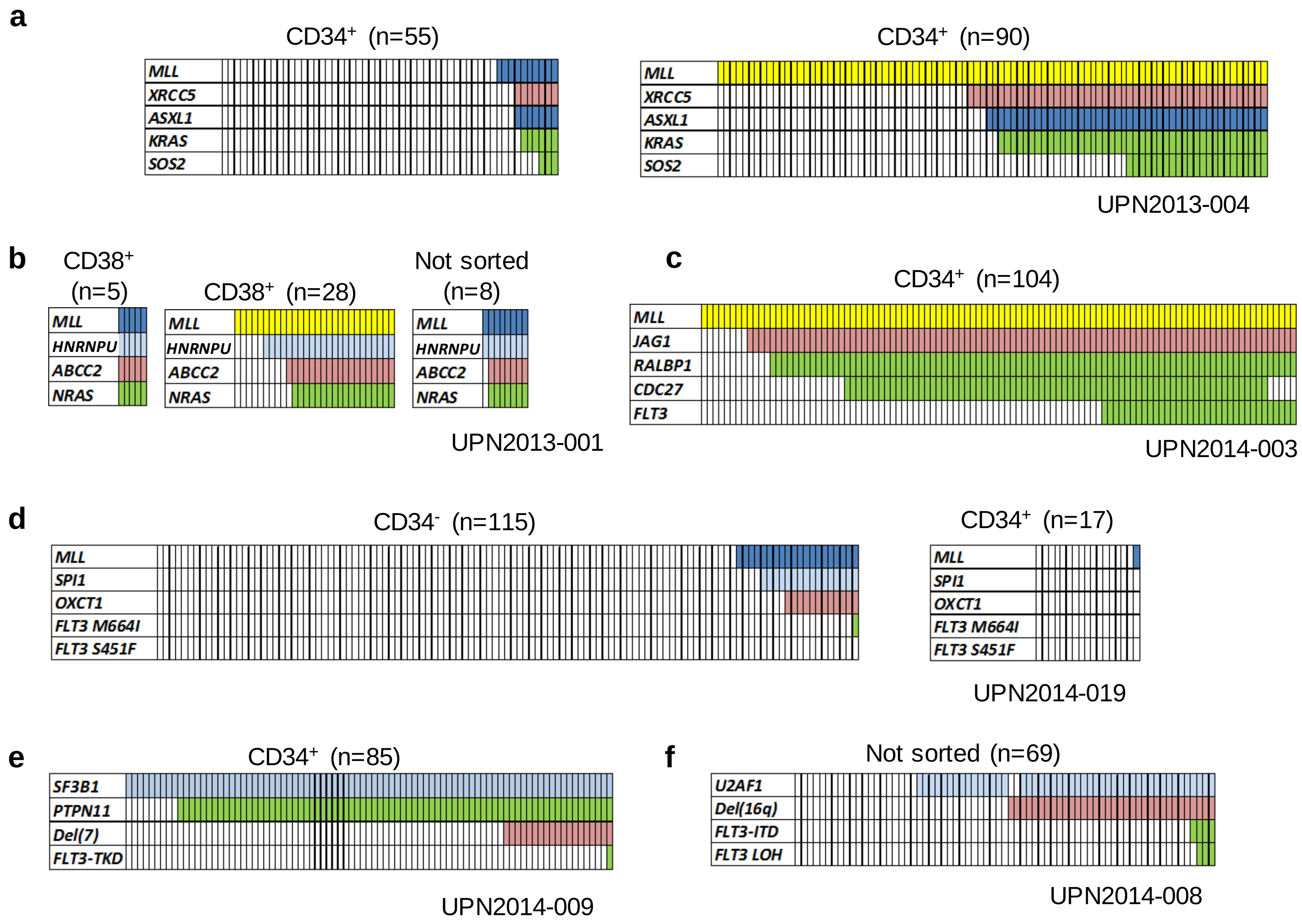

Supplementary Figure 3 | Summary of single cell derived colony assays in six patients with abnormal karyotype. a,b,c,d,e,f, Results of AS and FISH assays, as in **Fig.1**, for patients UPN2013-004 (**a**), UPN2013-001 (**b**), UPN2014-003 (**c**), UPN2014-019 (**d**), UPN2014-009 (**e**), and UPN2014-008 (**f**). For each patient, the cell phenotype before culture and the number of analysed colonies are indicated above the table. Yellow box means no data.

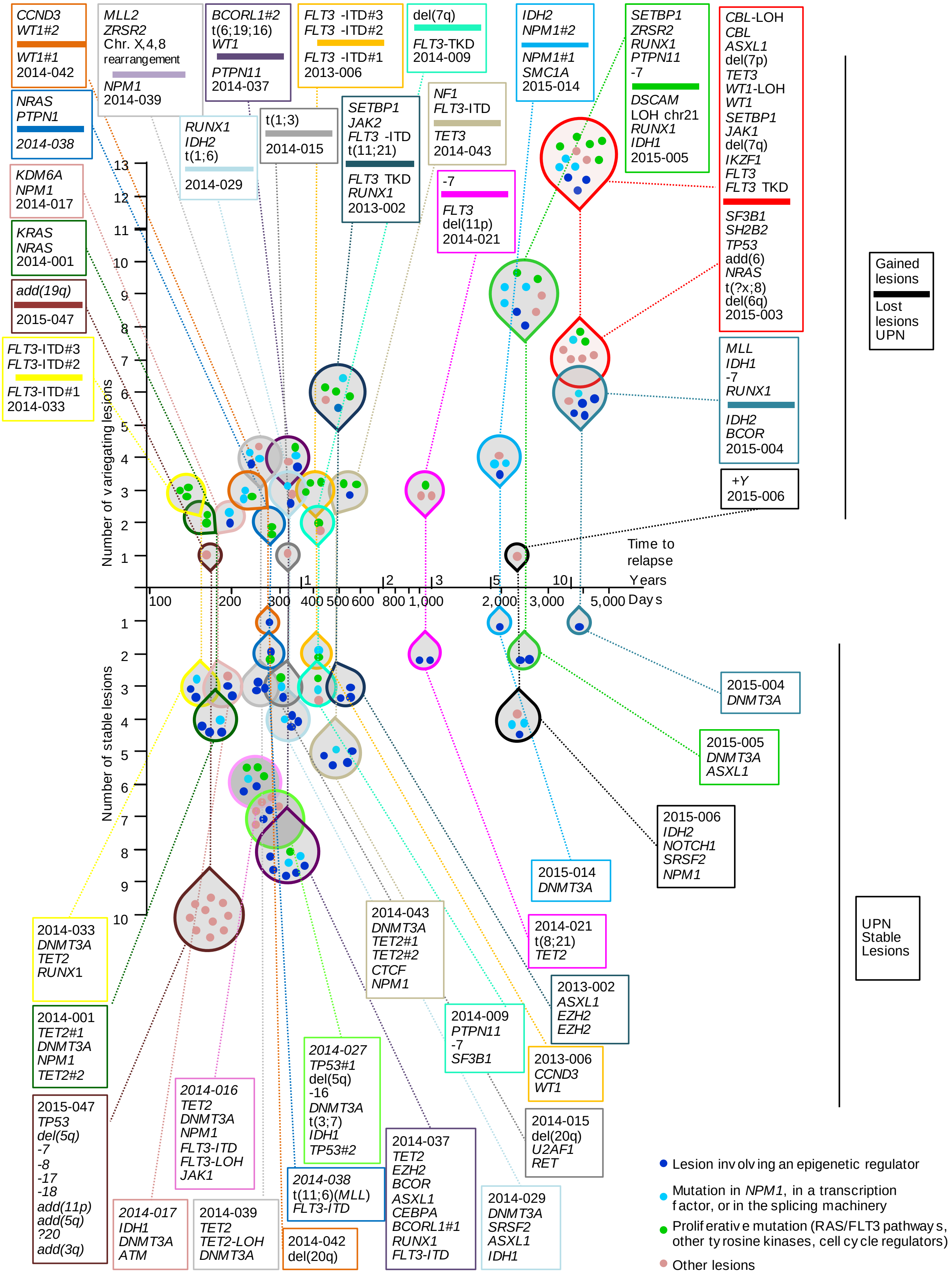

Supplementary Figure 4 | Temporal evolution of AML clones at relapse. Clonal composition of 22 AMLs at diagnosis and at relapse. The numbers of stable or variegating lesions are plotted as droplets above or below the time axis, respectively. Except for two cases (patients UPN2014-016 and UPN2014-027) with no changes in clonal composition (circles), coloured droplets of single patients are connected by vertical dashed lines. Droplets and circles are filled with dots that represent individual lesions from the four categories listed in the key, and summarized for each patient in connected boxes as indicated. Stable and variegating lesions are listed in coloured boxes below or above the time axis as indicated.

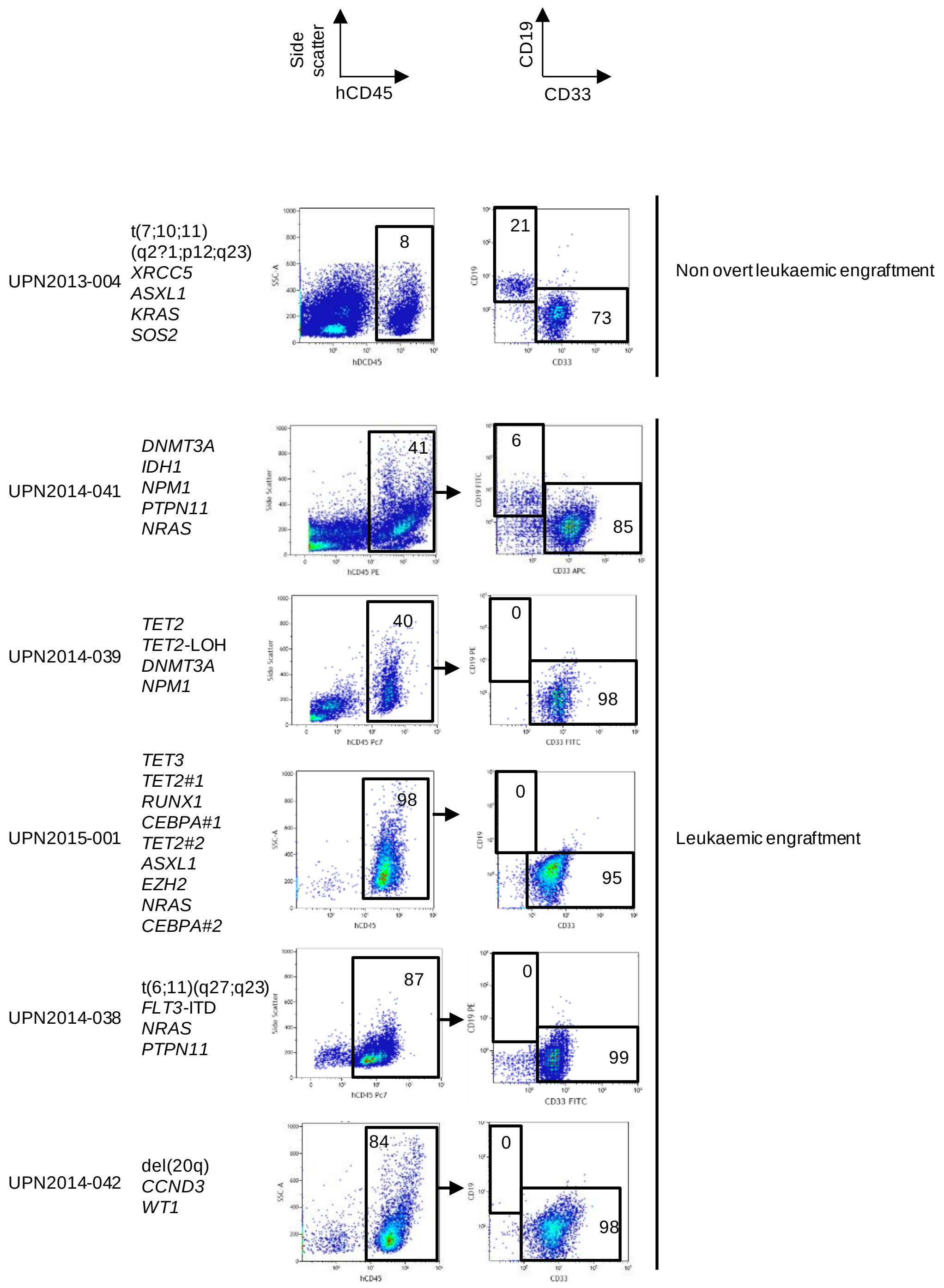

Supplementary Figure 5 | Leukaemic and non leukaemic repopulation of NSG bone marrow by AML cells. Flow cytometric analysis of NSG bone marrow repopulated with non leukaemic and leukaemic engraftments. Chromosomal aberrations and mutant genes detected in the injected samples are indicated.

a

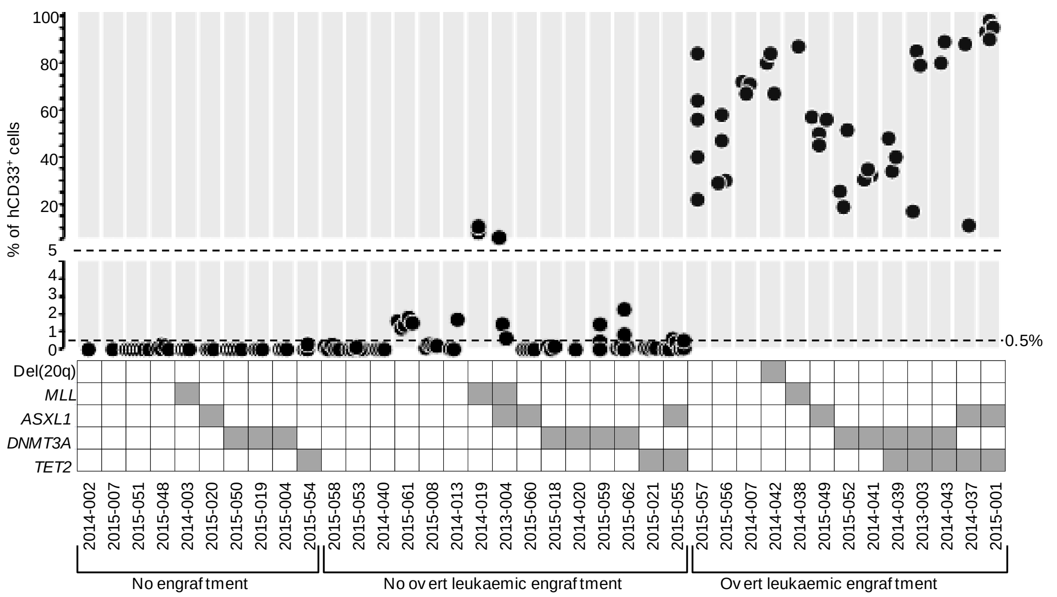

b

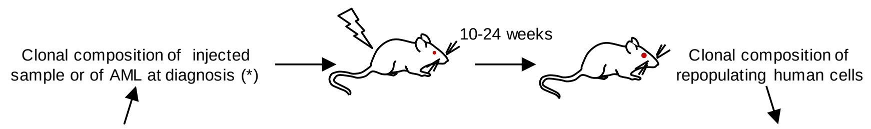

UPN	*IDH2*	der(11)	*DNMT3A*	*FBXW7*
2015-018	0.512	0.955	0.42	0.337

IDH2	der(11)	*DNMT3A*	*FBXW7*
0.536	ND	0.46	0.491

UPN	inv(16)	*KIT*
2014-007	0.903	0.435

inv(16)	*KIT*
Detected	0.482

UPN	*DNMT3A*	*IDH1*	*NPM1*	*PTPN11*	*NRAS*
2014-041	0.468	0.498	0.438	0.161	0.246

DNMT3A	*IDH1*	*NPM1*	*PTPN11*	*NRAS*
0.494	0.384	0.439	0.341	not detected
0.526	0.358	0.345	0.327	not detected

UPN	*TET2*	*SMC1A*	*DNMT3A*	*NPM1*	*FLT3-ITD*	*CBL*
2013-003	0.483	0.4777	0.439	0.413	0.412	0.07

TET2	*SMC1A*	*DNMT3A*	*NPM1*	*FLT3-ITD*	*CBL*
0.457	0.489	0.546	0.417	1	0.07

UPN	*DNMT3A*	*CTCF*	*TET2#1*	*TET2#2*	*NPM1*	*NF1*	*FLT3-ITD*
2014-043	0.47	0.472	0.448	0.502	0.513	0.495	0.379

DNMT3A	*CTCF*	*TET2#1*	*TET2#2*	*NPM1*	*NF1*	*FLT3-ITD*
0.4939	0.517	0.5049	0.509	0.441	0.462	0.334

UPN	*TET3*	*TET2#1*	*RUNX1*	*CEBPA*	*TET2#2*	*ASXL1*	*EZH2*	*NRAS*	*CEBPA*
2015-001	0.535	0.511	0.5	0.465	0.464	0.451	0.451	0.379	0.297

TET3	*TET2#1*	*RUNX1*	*CEBPA*	*TET2#2*	*ASXL1*	*EZH2*	*NRAS*	*CEBPA*
0.51	0.519	0.444	0.496	0.432	0.529	0.571	0.469	0.245

UPN	del(20q)	*CCND3*	*WT1*
2014-042	0.957	0.427	0.37

del(20q)	*CCND3*	*WT1*
0.97	0.489	0.289

UPN*	*JAK2*	*JAK2-LOH*	*TP53*	del(5q)	-7	der(17)	add(22)x2	del(1p)
2015-057	0.999	0.999	0.401	0.53	0.51	0.46	<0.46	<0.46

JAK2	*JAK2-LOH*	*TP53*	del(5q)	-7	der(17)	add(22)x2	del(1p)
0.998	0.998	0.992	>0.99	>0.99	ND	ND	ND

UPN	*TET2*	*DNMT3A*	*TET2-LOH*	*NPM1*	*ZRSR2*
2014-039	0.916	0.483	0.916	0.318	0.037

TET2	*DNMT3A*	*TET2-LOH*	*NPM1*	*ZRSR2*
Detected	Detected	ND	Detected	ND

Supplementary Figure 6 | Clonal composition of NSG repopulating leukaemia mirrors that of injected samples. a, Percentages of human $CD45^+$ $CD33^+$ ($hCD33^+$) cells in the bone marrow of NSG mice 8 to 43 weeks after injection of 5.10^6 mononuclear cells from 38 AML blood samples. The co-mutation table is as in Figure **3a**. **b,** Clonal composition of injected samples (left panel) and repopulating human cells after engraftment (right panel) in nine patients with overt leukaemic engraftment. VAFs are indicated for mutations. *FLT3-ITD* was detected by high resolution sizing of PCR products. Chromosomal aberrations were quantified using karyotype or interphasic FISH results and conversion of VAFs to cell fractions when informative SNPs were available. In post engraftment sample, Inv(16) was studied using RT-PCR for the CBFB-MYH11 transcript. *, For patient UPN2015-057, cells from relapse AML were injected, but only cells from diagnosis were available for clonal composition studies.

a

Experiment	Condition	Mouse	hCD45+	hCD19+	hCD33+
Exp#1	ShRNA_scramble	#136	1.2	ND	0.2
Exp#1	ShRNA_scramble	#137	12.1	ND	1.8
Exp#1	ShRNA_scramble	#138	3.5	ND	1.2
Exp#1	ShRNA_scramble	#139	0.2	ND	0.0
Exp#1	ShRNA_scramble	#140	42.3	ND	8.4
Exp#1	ShRNA_scramble	#144	12.3	ND	3.1
Exp#2	ShRNA_scramble	#910	20.4	15.4	3.4
Exp#2	ShRNA_scramble	#911	3.1	2.2	1.4
Exp#2	ShRNA_scramble	#918	13.3	6.7	7.4
Exp#2	ShRNA_scramble	#919	20.5	10.3	5.3
Exp#2	ShRNA_scramble	#921	48.5	20.5	16.2
Exp#3	ShRNA_scramble	#334	48.3	40.3	7.1
Exp#3	ShRNA_scramble	#338	37.6	29.9	5.5
Exp#3	ShRNA_scramble	#339	4.2	1.7	3.0
		median	12.8	12.8	3.2
	shRNA_Scramble	mean	19.1	15.9	4.6
		SEM	4.8	4.9	1.2

Experiment	Condition	Mouse	hCD45+	hCD19+	hCD33+
Exp#1	ShRNA_TET2	#132	60.0	ND	7.5
Exp#1	ShRNA_TET2	#135	20.8	ND	0.9
Exp#1	ShRNA_TET2	#141	20.3	ND	2.2
Exp#1	ShRNA_TET2	#142	30.8	ND	2.8
Exp#1	ShRNA_TET2	#143	22.4	ND	3.4
Exp#1	ShRNA_TET2	#149	57.0	ND	5.2
Exp#2	ShRNA_TET2	#906	26.2	22.2	2.9
Exp#2	ShRNA_TET2	#908	68.5	55.2	8.9
Exp#2	ShRNA_TET2	#912	41.2	18.9	15.2
Exp#2	ShRNA_TET2	#913	75.5	62.5	8.4
Exp#2	ShRNA_TET2	#917	90.9	29.7	47.5
Exp#2	ShRNA_TET2	#920	64.4	33.1	24.4
Exp#3	ShRNA_TET2	#324	53.7	48.6	5.8
Exp#3	ShRNA_TET2	#337	70.7	58.7	9.2
Exp#3	ShRNA_TET2	#345	50.2	29.8	5.1
		median	53.7	33.1	5.8
	shRNA_TET2	mean	50.2	39.8	10.0
		SEM	5.8	5.5	3.1

Mann-Whitney test	hCD45+	hCD19+	hCD33+
shRNA_scramble vs shRNA_TET2 p=	0.0003	0.0071	0.0505

b

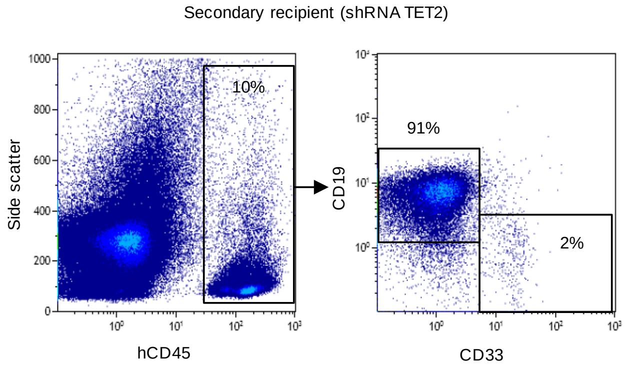

Supplementary Figure 7 | TET2 depletion enhances the repopulation of NSG mouse bone marrow by human cord blood CD34$^+$ cells. **a,** Percentages of human CD45, CD19, and CD33 positive cells in bone marrow of primary recipient mice 15-17 weeks after injection of cord blood CD34$^+$ cells transduced with lentiviruses expressing shRNAs designed against scramble and TET2 sequences. Statistical analysis was performed using the Mann-Whitney test. **b,** Flow cytometric analyses of the bone marrow from a secondary recipient mouse injected with human cells sorted from the bone marrow of mice #324, #337, and #345.

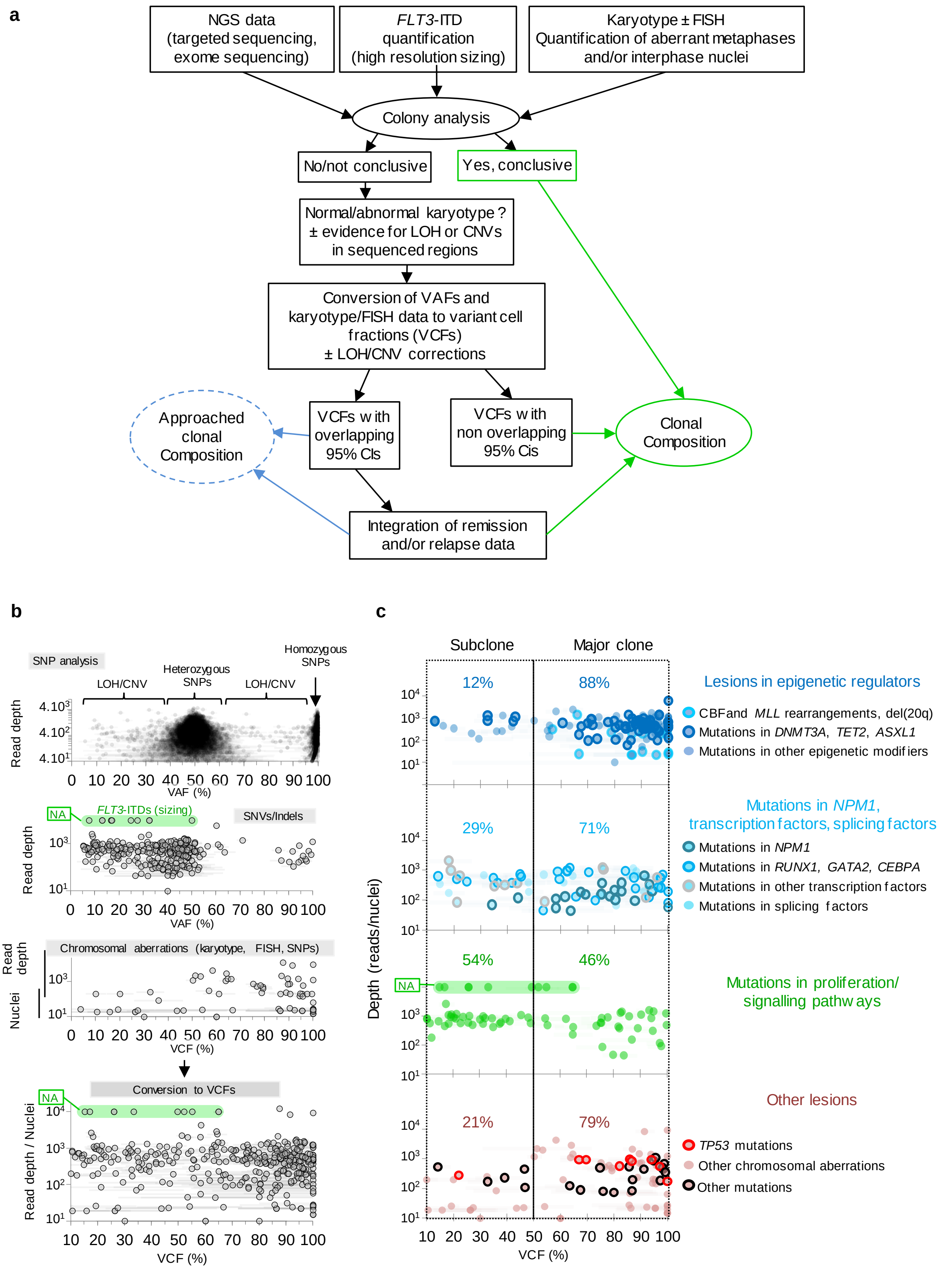

Supplementary Figure 8 | Multiparametric determination of clonal composition. **a,** Flow chart for the determination of the order of events in AML. **b,** Allele frequencies of SNPs, SNVs, and indels are plotted against read depth. Chromosomal aberrations were quantified by karyotypic and FISH analyses. When available, the quantification was inferred from VAFs of SNPs in imbalanced regions (LOH, CNVs). The values are plotted against the number of analysed nuclei (karyotype/FISH) or against the cumulated read depth of the SNPs used for quantification. Quantitative data were then converted to variant cell fractions (VCFs) for all detected chromosomal and genomic lesions above a threshold of 10% of cells. **c,** VCFs according to distinct categories of lesions.
Some *FLT3*-ITDs, only detected by the sizing technique, are plotted within green areas. Shaded error bars span 95% confidence intervals.

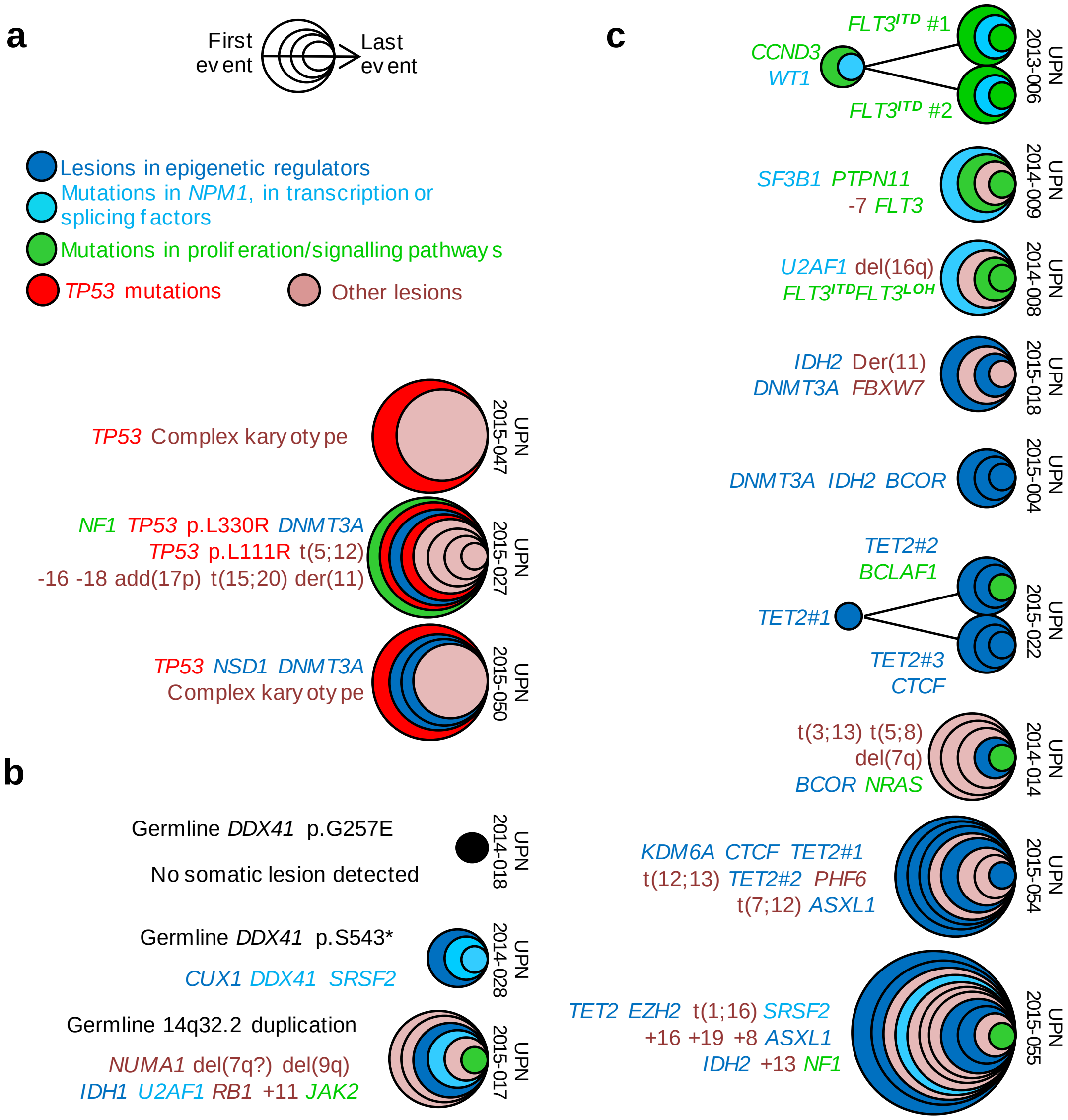

Supplementary Figure 9 | Clonal composition of 15/21 AMLs with no putative pre-leukaemic chromosomal aberration and no mutations in *NPM1*, *RUNX1*, *GATA2*, or *CEBPA*. a, AMLs with *TP53* mutations. **b**, AMLs with germline predispositions**. c,** Other cases. Internally tangent circles represent successive events. For patients UPN2015-047 and UPN2015-050 the chromosomal aberrations from complex karyotype were merged.

Supplementary Table 1 | Summary of patients' characteristics and clinical history

	characteristics at diagnosis											Treatment		Characteristics at relapse						treatment at relapse		Last follow-up	
UPN	Gender	Age	Prior myeloid malignancy	Prior exposition to cytotoxic chemotherapy or radiation therapy	WBC (x 10 9/L)	FAB	WHO	% Blast cells in BM	Karyotype	FLT3-ITD (sizing)	CEBPA mutation (HRM and sanger)	intensive chemotherapy	Allogeneic BMT in first CR	Relapse ?	Time to relapse (days)	%Blast cells in BM	Karyotype	FLT3-ITD (sizing)	CEBPA mutation (HRM and sanger)	Nature	Results	time from diagnosis (days)	alive at last follow-up
2013-001	M	31	No	No	17.6	M2	AML NOS	43	46, XY, t(11;19)(q23;p13)[14].ish t(11;19)(5'MLL+, 3'MLL-;5'MLL-, 3'MLL+)[3].nuc ish(MLLx2, 5'MLL sep 3'MLLx1)[199/200]	WT	WT	yes	yes	no								671	yes
2013-002	M	74	No	No	3.57	M2	AML NOS	44	46, XY	WT	WT	yes	no	yes	497	16	46, XY, t(11;21)(q13 or q21;q22)[5]/46, XY[17].ish t(11;22)(MLL-, 3'AML+;MLL+, 5'AML1+)[1].nuc ish(MLLx2)[200], (AML1x2)(5'AML1 sep 3'AML1x1)[68/200]	MUT (mut/wt=2, 3%)	WT	5-azacytidine	CR2	559	yes
2013-003	F	57	No	No	61	M1	AML with NPM1 mutation	91	46, XX	MUT (mut/wt=0.70)	WT	yes	yes	no								736	yes
2013-004	F	53	No	No	71	M5a	AML NOS	94	46, XX, der(7)t(7;10)(q2?1;p12), der(10)t(7;10;11)(q2?1;p12;q23)inv(11)(q13q23), der(11)t(10;11)(p12;q23)[19]/46, XX[3]	WT	WT	yes	no	no (died from sepsis in CR1, 108 days after diagnosis)								108	no
2013-006	F	18	No	No	12.2	M5a	AML NOS	85	46, XX	MUT	WT	yes	yes	yes (post BMT)	413	93	46, XX	MUT	WT	intensive chemotherapy	CR2	869	yes
2014-001	M	65	No	No	30	M5b	AML with NPM1 mutation	53	46, XY	WT	WT	yes	no	yes	173	11	46, XY	WT	WT	intensive chemotherapy	CR2	763	yes
2014-002	F	30	No	No	14.6	M2	AML with t(8;21)	35	46, XX, t(8;21)(q22;q22)[19]/46, XX[3]	WT	WT	yes	no	no								428	yes
2014-003	M	58	No	No	53	M4	AML NOS	61	46, XY, t(11;19)(q23;p13)[21]	WT	WT	yes	no	no								743	yes
2014-004	M	74	No	No	3.11	M2	AML NOS	24	46, XY	WT	WT	yes	no	no								953	yes
2014-006	M	71	No	No	6.7	M1	AML with NPM1 mutation	84	46, XY	WT	WT	yes	no	no								665	yes
2014-007	M	54	No	No	16.5	M4	AML with inv(16)	49	46, XY, inv(16)(p13;q22)[28]/46, XY[3]	WT	WT	yes	no	no								702	yes
2014-008	M	56	No	No	29	M5a	AML NOS	89	46, XY, del(16)(q12)[21]/46, XY[1]	MUT	WT	yes	yes	no								766	yes
2014-009	M	38	No	No	3.58	M4	AML NOS	38	45, XY, -7[5]/46, XY[22]	WT	WT	yes	yes	yes (post BMT)	414	9	46, XY, -7, +mar[cp8]/46, XY[9]	WT	WT	intensive chemotherapy	CR2	606	yes
2014-010	M	68	Yes (MDS)	No	9.54	M2	sAML	30	46, XY	WT	WT	yes	yes	no								566	yes
2014-013	F	48	No	No	1.17	M1	AML with NPM1 mutation	62	47, XX, +8[18]/46, XX[1]	MUT (mut/WT=0.33)	MUT	yes	yes	no								532	yes
2014-014	F	56	No	Yes (chemotherapy and radiation therapy for breast cancer)	8.26	M2	tAML	22	46, XX, add(3)(p11), add(5)(q11~12) or der(5)t(8;5)(q11~12;q21), del(7)(q21q36), add(9)(q11), del(12)(p11), ?add(14)(q24), -15, +mar[der(15)][cp24]	WT	WT	yes	yes	yes (post BMT)	194	ND (hemodilution)	47, X, add(X)(q21), del(1)(p2?2p3?6), add(3)(p11), add(5)(q11~12), del(7)(q21q36), add(14)(q24), -15, -21, +3mar[2]/46, XX[2]	WT	WT	no treatment		231	no
2014-015	M	74	No	No	1.6	M2	AML NOS	21	46, XY, del(20)(q11q13)[12]/46, XY[7]	WT	WT	yes	no	yes	322	12	46, XY, del(20)(q11q13)[1]/46, sl, t(3;7)(q21;q21) [21]/46, XY[2]	WT	WT	5-azacytidine	CR2	559	yes
2014-016	F	47	No	No	117	M5a	AML NOS	97	46, XX	MUT	WT	yes	no (relapse before BMT)	yes	240	48	46, XX	MUT	WT	sequential BMT	CR2	564	yes
2014-017	F	63	No	No	16.4	M1	AML with NPM1 mutation	95	46, XX	WT	WT	yes	yes (after relapse)	yes	175	9	46, XX	WT	WT	5-azacytidine	CR2	479	yes
2014-018	F	59	No	No	0.82	M1	AML NOS	65	46, XX	WT	WT	yes	yes	no								520	yes
2014-019	F	50	No	No	81.4	M5a	AML with t(9;11)	95	46, XX, t(9;11)(p22;q23)[6]/46, XX[15]	WT	WT	yes	no	no (died from sepsis in CR1, 76 days after diagnosis)								76	no
2014-020	M	55	No	No	29.7	M2	AML NOS	34	46, XY	MUT	WT	yes	yes	no								481	yes
2014-021	F	63	No	Yes (chemotherapy and radiation therapy for breast cancer)	193	M1	tAML	96	46, XX, t(8;21)(q22;q22), del(11)(p11p14) [21] / 46, XX [1]	WT	WT	yes	no	yes	1038	8	45, XX, t(8;21)(q22;q22)[1]/46, XX[23]	WT	WT	intensive chemotherapy	CR2	1517	yes
2014-022	F	81	No	No	2.6	M6/M7	AML NOS	44	46, XY	WT	WT	yes	no	no								639	yes
2014-024	F	72	No	No	71.3	M4	AML with NPM1 mutation	89	46, XY	WT	WT	yes	no	no								336	yes
2014-025	M	75	No	No	1.62	M2	AML NOS	24	46, XY	WT	WT	yes	no	no								376	yes
2014-027	F	72	No	No	4.94	M2	AML NOS	41	45, XX, add(3)(q21), del(5)(q21q34), der(7)t(3;7) (q13~21;q21 or q31), -16[18]	WT	WT	yes	yes	yes (post BMT)	287	25	45, XX, der(3)(pter->3q21~22::?), del(5)(q21q34), der(7)(7pter->7q21::3q21~22->3qter), -16[cp5]/46, XY[19]	WT	WT	5-azacytidine	failure	413	yes

UPN	Gender	Age	Prior myeloid malignancy	Prior exposition to cytotoxic chemotherapy or radiation therapy	WBC (x 10 9/L)	FAB	WHO	% Blast cells in BM	Karyotype	FLT3-ITD (sizing)	CEBPA mutation (HRM and sanger)	intensive chemotherapy	Allogeneic BMT in first CR	Relapse ?	Time to relapse (days)	%Blast cells in BM	Karyotype	FLT3-ITD (sizing)	CEBPA mutation (HRM and sanger)	Nature	Results	time from diagnosis (days)	alive at last follow-up
2014-028	M	53	No	No	0.95	M2	AML NOS	50	46, XY	WT	WT	yes	yes	no (donor cell leukemia)	763	69	48, XY, +6, der(13)t(13;?)(p11;?), +21 [12] /46, XY [3]	WT	WT	intensive chemotherapy	early death after treatment	845	no
2014-029	M	53	No	No	1.96	M2	AML NOS	37	46, XY	WT	WT	yes	no	yes	321	17	46, XY, t(1;6)(p3?4;q2?3)[4]/46, XY[2]	WT	WT	intensive chemotherapy	early death after treatment	415	no
2014-031	M	65	No	No	3.85	M2	AML NOS	44	48, XY, +10, +21[18]/48, idem, i(18)(q10)[5]/46, XY[3]	WT	WT	yes	yes	no								361	yes
2014-032	F	66	No	No	2.38	M2	AML NOS	33	46, XX	WT	WT	yes	yes	no								382	yes
2014-033	M	45	No	No	70.04	M1	AML NOS	87	46, XY	MUT (21bp ITD, mut/WT=0.13; 78bp ITD, mut/WT=0.13)	WT	yes	yes	yes	162	6	46, XY	MUT (mut/wt=3 %, 24bp ITD))	WT	sequential BMT	CR2	361	yes
2014-034	F	59	No	No	0.46	M1	AML wit CEBPA mutation	78	46, XX	WT	MUT	yes	yes	no								265	no
2014-036	M	44	No	No	39.6	M1	AML with NPM1 mutation	96	46, XY	MUT	WT	yes	yes	yes	284	61	ND	ND	ND	intensive chemotherapy	CR2	385	yes
2014-037	F	77	No	No	44	M5b	AML wit CEBPA mutation	62	46, XX	MUT	MUT	yes	no	yes	322	86	46, XX, t(6;19;16)(q2?1;q11;p13)[13]/46, XX[13].	MUT	MUT	no treatment		357	no
2014-038	M	31	No	No	96.4	M5a	AML NOS	88	46XY t(6;11)(q27;q23) [24]	MUT	WT	yes	yes	yes (post BMT)	273	80	46, XY, t(6;11)(q27;q23)[19]/46, XY[2]	MUT	WT	anti-FLT3 therapy	failure	427	yes
2014-039	M	65	No	No	50	M5b	AML with NPM1 mutation	74	46, XY	WT	WT	yes	no	yes	253	25	48, XY, der(4)?t(4;8)(q13;q22-23), +8, der(8)t(4;8)(q13;q22-23)del(8)(q23), der(8)t(X;8)(p11;p11)[12]/ 46, XY[16]	WT	WT	intensive chemotherapy	failure	362	no
2014-040	M	64	No	No	155	M0	AML NOS	89	46, XY, t(3;13)(p24;q13), t(5;8)(q14;q23) or t(5;8)(q12;q21), del(7)(q2?1q3?5)[15]	WT	WT	yes	no	yes	580	ND	ND	ND	ND	no treatment		610	yes
2014-041	M	40	No	No	90.7	M5a	AML with NPM1 mutation	70	46, XY	WT	WT	yes	no	no								812	yes
2014-042	M	48	No	No	2.3	M2	AML NOS	20	46, XY, del(20)(q12) [7] / 46, XY [20]	WT	WT	yes	yes	yes (post BMT)	272	65	FISH (20q12; 20q13.2); del(20q) in 201 /267 nuclei	WT	WT	5-azacytidine	failure	401	no
2014-043	F	73	No	Yes (radiation therapy for adenocarcinoma of the endometrium)	205	M1	AML NOS	93	46, XX	WT	WT	yes	no	yes	491	90	46, XX	MUT	WT	intensive chemotherapy	CR2	724	no
2015-001	M	68	Yes (MDS)	Yes (Radiation therapy for prostate cancer and 5azacytidine for MDS)	8.45	M2	sAML	25	46, XY	WT	MUT	no	no	no (no CR)								59	no
2015-002	F	66	No	No	1.2	M4eo	AML with inv(16)	28	46, XX, inv(16)(p13q22)[26]/47, sl, +8[1]	WT	WT	yes	no	no								950	yes
2015-003	F	19	No	No	3.6	M2	AML NOS	51	46XX, del(6)(q?12q?24)[5] / 46XX, -8, +t(?x;8)(?p10, ?q10)[5] / 46XX, add(6)(?p22)[2] / anomalies variables [3] / 46XX [4]	WT	WT	yes	no	yes (CMML-2)	3910	16	46, XX, del(7)(p11) [24] / 46, idem, del(7)(q22qter) [5]	WT	WT	multiple treatments	failure	5548	no
2015-004	F	57	No	Yes (chemotherapy and radiotherapy for breast cancer)	0.6	M1	tAML	76	46, XX	WT	WT	yes	no	yes (RAEB 2)	3939	14	45, XX, -7[24]	WT	WT	5-azacytidine	failure	4358	no
2015-005	F	56	No	No	1.7	M0	AML NOS	60	46, XX	WT	WT	yes	no	yes	2411	24	46XX;-7	WT	WT	intensive chemotherapy	failure	3062	no
2015-006	M	55	No	No	259	M1	AML NOS	94	47, XY, +Y	WT	WT	yes	no	yes	2312	93	46, XY	WT	WT	intensive chemotherapy	CR2	4054	yes
2015-007	M	45	No	No	117	M4eo	AML with inv(16)	NA	46, XY, inv(16)(p13q22)[16]/46, XY[8]	WT	WT	yes	no	no								389	yes
2015-008	M	20	No	No	107	M4eo	AML with inv(16)	70	47, XY, inv(16)(p13q22), +22[20]/46, XY[1].ish inv(16)(p13)(MYH11+, CBFB+)(q22)(CBFB+, MYH11+) [5].nuc ish(MYH11x3), (CBFBx3), (MYH11 con CBFBx2) [177/200]	WT	WT	yes	no	no (died at induction)								2	no
2015-014	M	52	No	No	8.35	M2	AML NOS	35	46, XY	WT	WT	yes	no	yes	1966	25	46, XY	WT	WT	intensive chemotherapy	CR2	2232	yes
2015-017	F	70	Yes (MPN)	Yes (hydroxyurea during one year)	2.24	M2	sAML	36	46, XX, ?add or del(7)(q?), del(9)(q12q33)[3]/47, idem, +11[3]/46, XX[4]	WT	WT	yes	yes	no								235	yes

	characteristics at diagnosis											Treatment		Characteristics at relapse						treatment at relapse		Last follow-up	
UPN	Gender	Age	Prior myeloid malignancy	Prior exposition to cytotoxic chemotherapy or radiation therapy	WBC (x 10 9/L)	FAB	WHO	% Blast cells in BM	Karyotype	FLT3-ITD (sizing)	CEBPA mutation (HRM and sanger)	intensive chemotherapy	Allogeneic BMT in first CR	Relapse ?	Time to relapse (days)	%Blast cells in BM	Karyotype	FLT3-ITD (sizing)	CEBPA mutation (HRM and sanger)	Nature	Results	time from diagnosis (days)	alive at last follow-up
2015-018	M	50	No	No	164	M1	AML NOS	97	47, XY, +der(11)(qter->q22~23::p14->qter)[9]/46, XY[1]	WT	WT	yes	no	no (No CR)								83	yes
2015-019	F	90	Yes (MDS)	No	32.6	NA	sAML	ND	ND	WT	WT	no	no	no								13	no
2015-020	M	62	No	No	2.09	M2	AML NOS	60	46, XX, del(3)(q14)[5], 46XY[12]	ND	ND	yes	no	yes	280	7	ND	MUT	WT			1329	yes
2015-021	M	88	No	No	77.9	NA	AML with NPM1 mutation	ND	45, X, -Y[20]	WT	WT	no	no	no								19	yes
2015-022	M	67	No	No	93.6	M1	AML with NPM1 mutation	96	46, XY	WT	MUT	yes	no	no								211	yes
2015-024	M	60	No	No	93	M1	AML with NPM1 mutation	92	46, XY	WT	WT	yes	no	no								187	yes
2015-027	M	69	No	No	60	M5a	AML NOS	75	42~43, XY, der(5)?add(5)(p14)t(5;12)(q11;q11), der(11)del(11)(q14q22)amp(11)(q23), -12, -15, -16, add(17)(p11), -18, der(20)t(15;20)(q2?1;q1?3), +2mar[cp21]	WT	WT	no	no	no (died at induction)								4	no
2015-047	M	63	No	Yes (Radiation therapy for prostate cancer)	4.09	M1	tAML	55	44, XY, del(5)(q22q34), -7, -8, -17, -18, ?20, +mar1 [?der(7)del(7)(q?)?add(7)(p?)?add(7)(q?)], +mar2 [?der(17)(add(17)(p?)add(q?25)][18]/43-45, XY, add(3)(q1?), del(5)(q22q34)[2], add(5)(q?21)[3], -7, -8, add(11)(p1?3)[2], -17, -18, ?20, +mar1, +mar2[cp5]	WT	WT	yes	no	yes	164	73	43~44, XY, add(3)(q1?), del(5)(q22q34), -7, -8, add(11)(p13), -17, -18, add(19)(q13), add(20)(q1?2), +mar1[?der(7)del(7)(q?)?add(7)(p?)?add(7)(q?) or i(7)(p10], +mar2 [?der(17)(add(17)(p?)add(q?25)][cp8]/46, XY[2]	WT	WT	no treatment		177	yes
2015-048	M	60	No	No	90	M4eo	AML with inv(16)	31	46, XY, inv(16)(p13q22)[16]/46, ?idem[3]/46, XY[2]	WT	WT	yes	no	no (died at induction)								33	no
2015-049	M	65	No	No	11.4	M0	AML NOS	65	46, XY	WT	WT	yes	no	yes	365	63	46, XY, t(9;22)(q31;q11)[5]/46, XY[13]	ND	ND			580	no
2015-050	F	68	No	No	1.33	M2	AML NOS	23	56, XX, +2, add(5)(q?22), +6, +8, +8, +9, +11, +add(11)(p12), +13, del(17)(p1?2), +19, +22[9]/55, X, add(X)(q2?1), +2, add(5)(q?22), add(5)(q3?3), +6, +8, +8, +11, +11, 13, +19, +22[3]/57, sl2, +add(X)(q2?1), +10, -22[7]/46, XX[3]	WT	WT	no	no	no (no CR)								453	no
2015-051	M	42	No	No	69	M2	AML wit CEBPA mutation	71	46, XY	WT	WT	yes	yes	no								2009	yes
2015-052	F	66	No	No	77.5	M4	AML with NPM1 mutation	40	46, XX	WT	WT	yes	no	no (died at induction)								18	no
2015-053	F	35	No	No	195	M4	AML wit CEBPA mutation	85	46, XY	MUT	WT	yes	no	no								2438	yes
2015-054	F	79	Yes (CMML and CLL)	Yes (chlorambucil for CLL ; hydroxyurea and 5azacytidine for CMML)	5.59	M2	sAML	23	46, XX, t(12;13)(q13;q24)[4]/46, idem, t(7;12)(q22;q24)[24]	WT	WT	no	no	no (no CR)								438	no
2015-055	M	74	Yes (MDS)	Yes (5azacytidine for MDS)	13.17	M5a	sAML	55	46, XY, der16t(1;16)(q11;11)[2]/50, idem, +8, +13, +16, +19[24]	WT	WT	no	no	no (no CR)								55	yes
2015-056	F	48	No	Yes (Chemotherapy and radiation therapy for synoviosarcoma)	7.2	M0	tAML	58	45, XX, del(5)(q21-22q33-34), -7[21]/46, XX[5]	WT	WT	yes	no	no (no CR)								128	no
2015-057	F	60	Yes (MPN)	yes (pipobroman and hydroxyurea during 20 years)	13.2	M6v	sAML	60	45, XX, del(1)(p?34), del(5)(q12q34), -7, der(17)t(17;?)(?q;?;?), add(22)(p11)x2, var[21]/46XX[2]	WT	WT	yes	no	yes	119	61	ND	ND	ND			213	no
2015-058	M	76	No	No	64.61	M5a	AML NOS	87	46, XY	MUT	WT	yes	no	yes	431	ND	MUT	ND	ND			648	no
2015-059	M	37	No	No	87.47	M5b	AML with NPM1 mutation	90	46, XY	MUT	WT	yes	no	no								305	yes
2015-060	M	71	Yes (MDS)	No	2.01	M2	sAML	32	46, XY	WT	WT	no	no	no (no CR)								105	no
2015-061	M	56	No	No	11.18	M2	AML with NPM1 mutation	32	46, XY	WT	WT	yes	no	no								1450	yes
2015-062	F	50	No	No	8.95	M4	AML with NPM1 mutation	70	46, X, add(X)(p?21-23) or ?i(X)(q10)[15]/46, XX[7]	WT	WT	yes	no	no								1861	yes

MPN: myeloproliferative neoplasm; MDS: myelodysplastic syndrome; CLL: chronic lymhocytic leukaemia; CMML: chronic myelomonocytic leukaemia; BMT: bone marrow transplantation; MUT: mutant; WT: wild type; NOS: not otherwise specified; CR: complete remission, ND : no data

Supplementary Table 2 | list of the 122 genes included in the targeted re-sequencing panel

ABCB11	*DNM2*	*MAML1*	*SH2B3*
ACSS3	*DNMT3A*	*MAP2K1*	*SHKBP1*
AKAP13	*DOK2*	*MAP2K2*	*SMC1A*
APH1A	*DSCAM*	*MLL*	*SMC3*
ARHGEF2	*EED*	*MLL2*	*SPI1*
ASXL1	*EGR1*	*MPL*	*SRSF2*
ASXL2	*ERCC2*	*MSR1*	*SSRP1*
ATM	*ETV6*	*MYBL2*	*STAG1*
BCLAF1	*EZH2*	*NCOA7*	*STAG2*
BCOR	*FBXW7*	*NCSTN*	*STAT3*
BCORL1	*FLT3*	*NF1*	*SUZ12*
BOC	*FOXP1*	*NMNAT2*	*TEK*
BRAF	*FZD1*	*NOTCH1*	*TERC*
BRPF1	*GATA1*	*NOTCH2*	*TERT*
CBL	*GATA2*	*NPM1*	*TET2*
CBLB	*GATAD2B*	*NRAS*	*TET3*
CCND3	*GBP4*	*NSD1*	*TLE4*
CDKN2A	*GDF5*	*NUMA1*	*TP53*
CDKN2B	*GLI1*	*PBRM1*	*TP73*
CEBPA	*HJURP*	*PDS5B*	*TYK2*
CHEK2	*HRAS*	*PHF6*	*U2AF1*
CSF3R	*IDH1*	*PTEN*	*WAC*
CTCF	*IDH2*	*PTPN11*	*WT1*
CUL3	*IKZF1*	*RAD21*	*XRCC1*
CUX1	*ITGAX*	*RB1*	*XRCC3*
CUX2	*JAK1*	*RBMX*	*ZRSR2*
CXXC4	*JAK2*	*RET*	
DAAM2	*JAK3*	*RUNX1*	
DAXX	*JARID2*	*SETBP1*	
DDX1	*KDM6A*	*SF3A1*	
DDX41	*KIT*	*SF3B1*	
DIS3	*KRAS*	*SH2B2*	

Supplementary Table 3 | List of somatic variants detected with the targeted resequencing panel at diagnosis. The list of all detected variants is in **Supplementary Data Set 1** (Excel file).

patient	gene	chromosome	location	reference base	altered base	frequency	AA	NM
2013-001	NRAS	1	115256528	T	A	0.391	Q61H	NM_002524
2013-002	ASXL1	20	31021118	C	T	0.355	Q373*	NM_015338
2013-002	EZH2	7	148512036	A	C	0.322	C504G	NM_152998
2013-002	EZH2	7	148523591	G	A	0.290	R249*	NM_152998
2013-002	FLT3	13	28592628	A	T	0.088	D839E	NM_004119
2013-002	RUNX1	21	36259211	T	TGTCG	0.316	D66fs	NM_001001890
2013-003	CBL	11	119149290	C	T	0.074	P433L	NM_005188
2013-003	DNMT3A	2	25457242	C	T	0.434	R882H	NM_022552
2013-003	NPM1	5	170837543	C	CTCTG	0.413	L258fs	NM_002520
2013-003	SMC1A	X	53432045	G	A	0.478	R699C	NM_006306
2013-003	TET2	4	106164916	C	T	0.483	R1262W	NM_001127208
2013-004	ASXL1	20	31022572	AGT	A	0.402	K686fs	NM_015338
2013-004	KRAS	12	25398281	C	T	0.511	G13D	NM_004985
2013-006	CCND3	6	41903745	C	CG	0.467	R271fs	NM_001136017
2013-006	WT1	11	32417941	C	CACTTTTT	0.378	R141fs	NM_000378
2014-001	DNMT3A	2	25457242	C	T	0.399	R882H	NM_022552
2014-001	KRAS	12	25380275	T	G	0.132	Q61H	NM_004985
2014-001	NPM1	5	170837543	C	CTCTG	0.337	L258fs	NM_002520
2014-001	NRAS	1	115256530	G	T	0.098	Q61K	NM_002524
2014-001	TET2	4	106156978	CT	C	0.160	L627fs	NM_001127208
2014-001	TET2	4	106180853	A	G	0.484	Y1294C	NM_001127208
2014-002	NRAS	1	115258748	C	T	0.407	G12S	NM_002524
2014-003	FLT3	13	28602329	G	A	0.150	A680V	NM_004119
2014-006	DSCAM	21	41741170	A	G	0.368	S171P	NM_001389
2014-006	IDH1	2	209113113	G	C	0.351	R132G	NM_005896
2014-006	NPM1	5	170837543	C	CTCTG	0.377	L258fs	NM_002520
2014-007	KIT	4	55599320	G	T	0.432	D812Y	NM_001093772
2014-008	U2AF1	21	44524456	G	A	0.464	S34F	NM_001025203
2014-009	FLT3	13	28592642	C	A	0.120	D835Y	NM_004119
2014-009	PTPN11	12	112915455	T	C	0.446	F285S	NM_002834
2014-009	SF3B1	2	198267359	C	A	0.402	K666N	NM_012433
2014-010	RUNX1	21	36231773	C	T	0.489	R177Q	NM_001001890
2014-013	CEBPA	19	33793199	TGCGCGGGGCCC	T	0.520	p.A37fs	NM_004364
2014-013	NPM1	5	170837543	C	CTCTG	0.466	L258fs	NM_002520
2014-013	PDS5B	13	33306277	A	AC	0.164	P722fs	NM_015032
2014-013	WT1	11	32456289	G	T	0.193	Y201*	NM_000378
2014-013	WT1	11	32417911	A	ACC	0.119	S152fs	NM_000378
2014-014	TP53	17	7577094	G	A	0.334	R282W	NM_005475
2014-014	TP53	17	7577114	C	T	0.347	C276Y	NM_000546
2014-015	RET	10	43612093	G	A	0.173	G733D	NM_020630
2014-015	U2AF1	21	44524456	G	A	0.268	S34F	NM_001025203
2014-016	TET2	4	106164088	A	G	0.119	splice site	NM_001127208
2014-016	DNMT3A	2	25463175	A	T	0.457	L773H	NM_022552
2014-016	JAK1	1	65301882	A	C	0.430	L1053V	NM_002227
2014-016	NPM1	5	170837544	T	TCTGC	0.413	L258fs	NM_002520
2014-017	ATM	11	108186629	C	G	0.458	P2029R	NM_000051
2014-017	DNMT3A	2	25463287	G	A	0.461	R736C	NM_022552
2014-017	IDH1	2	209113113	G	A	0.483	R132C	NM_005896
2014-017	KDM6A	X	44920601	TG	T	0.409	V455fs	NM_021140
2014-017	NPM1	5	170837543	C	CTCTG	0.470	L258fs	NM_002520
2014-018	CUX1	7	101758539	AG	A	0.080	E221fs	NM_001202543
2014-018	DDX41	5	176939370	C	T	0.219	R525H	NM_016222
2014-018	SRSF2	17	74732956	G	A	0.262	P96L	NM_001195427
2014-019	FLT3	13	28602376	C	T	0.111	M664I	NM_004119
2014-019	FLT3	13	28610138	G	A	0.120	S451F	NM_004119
2014-019	SPI1	11	47377080	G	T	0.376	R171S	NM_003120
2014-020	DNMT3A	2	25457243	G	A	0.464	R882C	NM_022552
2014-020	FLT3	13	28592641	T	A	0.241	D835V	NM_004119
2014-020	FLT3	13	28608259	A	ATATTCATATTCTCTGAAATCAACGTAG	0.093		NM_004119
2014-020	GATA2	3	128202770	T	C	0.109	N317S	NM_001145661
2014-021	FLT3	13	28608317	T	G	0.475	Q580P	NM_004119
2014-021	TET2	4	106157554	TATAGTCAGACC	T	0.500	Y819fs	NM_001127208
2014-022	BCLAF1	6	136599694	G	A	0.199	R107C	NM_001077440
2014-022	CTCF	16	67650719	C	T	0.063	R14C	NM_001191022
2014-022	SRSF2	17	74732959	G	T	0.305	P95T	NM_001195427
2014-022	TET2	4	106157551	CCT	C	0.278	P818fs	NM_001127208
2014-022	TET2	4	106158106	TG	T	0.426	W1003fs	NM_001127208
2014-022	TET2	4	106196627	C	T	0.065	Q1654*	NM_001127208
2014-024	NPM1	5	170837543	C	CTCTG	0.373	L258fs	NM_002520
2014-024	TET2	4	106164778	C	T	0.388	R1216*	NM_001127208
2014-024	TET2	4	106197401	C	T	0.427	H1912Y	NM_001127208
2014-025	IDH2	15	90631934	C	T	0.475	R140Q	NM_002168
2014-025	SRSF2	17	74732935	CGGCGGCTGTGGTGTGAGTCCGGGG	C	0.455	PPDSHHSRR95R	NM_001195427
2014-027	DNMT3A	2	25468153	A	G	0.440	L508P	NM_022552
2014-027	IDH1	2	209113112	C	T	0.439	R132H	NM_005896
2014-027	TP53	17	7578190	T	C	0.485	Y220C	NM_000546
2014-027	TP53	17	7578454	G	A	0.410	A159V	NM_000546
2014-028	CUX1	7	101847813	TCCAGGGCCAGCAG	T	0.189	V1017fs	NM_001202543
2014-028	DDX41	5	176939370	C	T	0.155	R525H	NM_016222
2014-028	SRSF2	17	74732959	G	A	0.074	P95S	NM_001195427
2014-029	ASXL1	20	31022402	TCACCACTGCCATAGAGAGGCGGC	T	0.330	H630fs	NM_015338
2014-029	DNMT3A	2	25457242	C	T	0.351	R882H	NM_022552
2014-029	IDH1	2	209113113	G	A	0.451	R132C	NM_005896
2014-029	IDH2	15	90631934	C	T	0.007	R140Q	NM_002168
2014-029	RUNX1	21	36164838	C	CG	0.014	R319fs	NM_001001890
2014-029	SRSF2	17	74732959	G	T	0.020	P95T	NM_001195427
2014-031	BCOR	X	39932579	G	GT	0.217	H674fs	NM_001123383
2014-031	ETV6	12	11992103	G	GACTTTTCCTT	0.104	D65fs	NM_001987
2014-031	RUNX1	21	36231792	C	T	0.320	D171N	NM_001001890

patient	gene	chromosome	location	reference base	altered base	frequency	AA	NM
2014-032	DNMT3A	2	25457242	C	T	0.337	R882H	NM_022552
2014-032	RUNX1	21	36252876	C	G	0.071	R135S	NM_001001890
2014-032	RUNX1	21	36252937	G	GCC	0.226	A115fs	NM_001001890
2014-033	DNMT3A	2	25457243	G	A	0.430	R882C	NM_022552
2014-033	RUNX1	21	36164638	A	AG	0.352	S386fs	NM_001001890
2014-033	TET2	4	106158509	G	A	0.519	G1137D	NM_001127208
2014-034	BCOR	X	39922191	G	GT	0.370	N1293fs	NM_001123383
2014-034	BCOR	X	39911571	C	A	0.145	V1635L	NM_001123384
2014-034	CEBPA	19	33793174	CGGGGCGG	C	0.124	A47fs	NM_004364
2014-034	IDH2	15	90631839	T	A	0.403	R172W	NM_002168
2014-036	DNMT3A	2	25463576	A	T	0.447	D702E	NM_022552
2014-036	NPM1	5	170837543	C	CTCTG	0.500	L258fs	NM_002520
2014-037	ASXL1	20	31022402	TCACCACTGCCATAGAGAGGCGGC	T	0.420	H630fs	NM_015338
2014-037	BCOR	X	39932171	G	A	0.446	R810*	NM_001123383
2014-037	BCORL1	X	129155104	C	T	0.249	R1196*	NM_021946
2014-037	CEBPA	19	33793191	CG	C	0.350	P43fs	NM_004364
2014-037	EZH2	7	148506458	T	A	0.400	K629M	NM_001203249
2014-037	FLT3	13	28608242	A	AACTCCCATTTGAGATCAT	0.207	-605MISNGS	NM_004119
2014-037	PTPN11	12	112926910	G	T	0.137	Q510H	NM_002834
2014-037	RUNX1	21	36252866	G	GTGTC	0.106	V137fs	NM_001001890
2014-037	TET2	4	106196480	G	T	0.401	A1605S	NM_001127208
2014-037	WT1	11	32421575	G	C	0.016	Y110*	NM_001198552
2014-038	FLT3	13	28608251	T	TTGAGATCATATTCATATTCTC	0.277	-602ENMNMIS	NM_004119
2014-039	DNMT3A	2	25457242	C	T	0.481	R882H	NM_022552
2014-039	NPM1	5	170837543	C	CTCTG	0.318	L258fs	NM_002520
2014-039	TET2	4	106164913	C	T	0.914	R1261C	NM_001127208
2014-039	ZRSR2	X	15822319	A	T	0.000	E133V	NM_005089
2014-040	BCOR	X	39923053	C	A	0.946	E1167*	NM_001123384
2014-040	NRAS	1	115256529	T	C	0.473	Q61R	NM_002524
2014-041	DNMT3A	2	25457243	G	A	0.469	R882C	NM_022552
2014-041	IDH1	2	209113112	C	T	0.440	R132H	NM_005896
2014-041	NPM1	5	170837543	C	CTCTG	0.438	L258fs	NM_002520
2014-041	NRAS	1	115256530	G	T	0.246	Q61K	NM_002524
2014-041	PTPN11	12	112888199	C	T	0.161	A72V	NM_002834
2014-042	CCND3	6	41903698	CATCT	C	0.017	T285fs	NM_001136017
2014-042	WT1	11	32417907	G	GCCGA	0.176	A153fs	NM_000378
2014-042	WT1	11	32417924	C	CGGGGCTG	0.020	P148fs	NM_000378
2014-043	CTCF	16	67654643	G	A	0.530	R377H	NM_006565
2014-043	DNMT3A	2	25469541	C	T	0.545	W409*	NM_022552
2014-043	NPM1	5	170837545	C	CTGTA	0.291	W259fs	NM_002520
2014-043	TET2	4	106190795	G	A	0.475	C1358Y	NM_001127208
2014-043	TET2	4	106197002	G	GC	0.421	A1779fs	NM_001127208
2014-043	TET3	2	74320089	C	T	0.274	R899*	NM_144993
2015-001	ASXL1	20	31022405	C	CG	0.451	H631fs	NM_015338
2015-001	CEBPA	19	33792438	C	T	0.461	A295T	NM_004364
2015-001	CEBPA	19	33793191	G	GC	0.297	A44fs	NM_004364
2015-001	EZH2	7	148506433	A	C	0.442	N637K	NM_001203249
2015-001	NRAS	1	115258747	C	G	0.378	G12A	NM_002524
2015-001	RUNX1	21	36206720	C	CT	0.500	Q237fs	NM_001001890
2015-001	TET2	4	106157506	C	T	0.463	Q803*	NM_001127208
2015-001	TET2	4	106180783	TG	T	0.511	C1271fs	NM_001127208
2015-001	TET3	2	74327616	A	AG	0.535	N1099fs	NM_144993
2015-002	NRAS	1	115256530	G	T	0.325	Q61K	NM_002524
2015-002	NRAS	1	115258747	C	T	0.058	G12D	NM_002524
2015-002	TET2	4	106156095	C	A	0.432	C332*	NM_001127208
2015-003	NRAS	1	115258744	C	T	0.376	G13D	NM_002524
2015-003	SF3B1	2	198266818	C	CTGA	0.099	-705S	NM_012433
2015-003	SH2B2	7	101957773	C	G	0.129	A392G	NM_020979
2015-003	TP53	17	7577120	C	T	0.110	R273H	NM_000546
2015-004	BCOR	X	39932876	T	TG	0.409	N575fs	NM_001123383
2015-004	DNMT3A	2	25463536	C	T	0.466	V716I	NM_022552
2015-004	IDH2	15	90631838	C	T	0.464	R172K	NM_002168
2015-005	ASXL1	20	31023159	C	T	0.302	Q882*	NM_015338
2015-005	DNMT3A	2	25457242	C	T	0.358	R882H	NM_022552
2015-005	DSCAM	21	41414478	C	A	0.564	E1836*	NM_001389
2015-005	IDH1	2	209113113	G	A	0.315	R132C	NM_005896
2015-005	RUNX1	21	36259304	CGTCCGGG	C	0.000	A33fs	NM_001001890
2015-006	IDH2	15	90631934	C	T	0.512	R140Q	NM_002168
2015-006	NOTCH1	9	139397775	C	T	0.474	V1676I	NM_017617
2015-006	NPM1	5	170837543	C	CTCTG	0.402	L258fs	NM_002520
2015-006	SRSF2	17	74732959	G	T	0.443	P95T	NM_001195427
2015-007	CSF3R	1	36932135	C	CG	0.100	L778fs	NM_000760
2015-007	FLT3	13	28602380	T	C	0.052	K663R	NM_004119
2015-007	KIT	4	55589766	GACTTACG	G	0.176	Y418fs	NM_000222
2015-008	ATM	11	108143317	C	T	0.232	L1046F	NM_000051
2015-008	KIT	4	55599321	A	T	0.323	D812V	NM_001093772
2015-008	KIT	4	55589776	TTACGACAGGCTCG	TTACTACGACTGTC	0.052	YDRLV417YYDCL	NM_000222
2015-014	DNMT3A	2	25467172	CCCA	C	0.453	VG567G	NM_022552
2015-014	NPM1	5	170837543	C	CTCTG	0.400	L258fs	NM_002520
2015-014	SMC1A	X	53430550	G	A	0.865	R790W	NM_006306
2015-017	IDH1	2	209113113	G	A	0.278	R132C	NM_005896
2015-017	JAK2	9	5073770	G	T	0.106	V617F	NM_004972
2015-017	NUMA1	11	71718270	G	A	0.373	R1810C	NM_006185
2015-017	RB1	13	48878084	CGCCGCCGCT	C	0.285	AAA13-	NM_000321
2015-017	RB1	13	48878091	G	A	0.286	A15T	NM_000321
2015-017	U2AF1	21	44524456	G	A	0.239	S34F	NM_001025203
2015-018	DNMT3A	2	25457242	C	T	0.423	R882H	NM_022552
2015-018	FBXW7	4	153271203	T	G	0.337	E112A	NM_018315
2015-018	IDH2	15	90631934	C	T	0.512	R140Q	NM_002168
2015-019	BCOR	X	39913178	A	AG	0.491	L1612fs	NM_001123383
2015-019	DNMT3A	2	25463302	A	G	0.445	F731L	NM_022552
2015-019	FLT3	13	28592640	A	C	0.273	D835E	NM_004119
2015-019	IDH2	15	90631838	C	T	0.452	R172K	NM_002168
2015-019	RUNX1	21	36259202	A	AGTT	0.294	N69KT	NM_001001890
2015-019	STAG2	X	123179197	C	T	0.071	R216*	NM_001042749
2015-021	NPM1	5	170837543	C	CTCTG	0.376	L258fs	NM_002520
2015-021	TET2	4	106190797	C	T	0.955	R1359C	NM_001127208

patient	gene	chromosome	location	reference base	altered base	frequency	AA	NM
2015-022	CEBPA	19	33792553	C	CA	0.267	L256fs	NM_004364
2015-022	DNMT3A	2	25471025	C	CA	0.480	P245fs	NM_022552
2015-022	NPM1	5	170837545	C	CTGCA	0.171	W259fs	NM_002520
2015-022	TET2	4	106158468	TG	T	0.940	V1124fs	NM_001127208
2015-024	NPM1	5	170837543	C	CTCTG	0.391	L258fs	NM_002520
2015-024	TET2	4	106164778	C	T	0.508	R1216*	NM_001127208
2015-024	TET2	4	106196920	TA	T	0.475	K1752fs	NM_001127208
2015-027	DNMT3A	2	25458661	T	C	0.444	N838D	NM_022552
2015-027	NF1	17	29553477	A	AC	0.487	T676fs	NM_000267
2015-027	TP53	17	7576857	A	C	0.470	L330R	NM_000546
2015-027	TP53	17	7579355	A	C	0.429	L111R	NM_000546
2015-047	TP53	17	7578394	T	C	0.432	H179R	NM_000546
2015-048	NRAS	1	115258747	C	T	0.452	G12D	NM_002524
2015-048	SPI1	11	47381470	CATG	C	0.441	HM87Q	NM_003120
2015-049	ASXL1	20	31022402	TCACCACTGCCATAGAGAGGCGGC	T	0.510	H630fs	NM_015338
2015-049	BCOR	X	39934013	T	TGTAA	0.919	M196fs	NM_001123383
2015-049	ETV6	12	12006463	C	CTGCT	0.459	P144fs	NM_001987
2015-049	RUNX1	21	36206875	G	A	0.364	Q186*	NM_001001890
2015-049	SRSF2	17	74732959	G	GGGC	0.481	R94RR	NM_001195427
2015-050	DNMT3A	2	25457158	G	A	0.616	A910V	NM_022552
2015-050	NSD1	5	176720934	G	C	0.634	G1920R	NM_172349
2015-050	TP53	17	7577114	C	T	0.897	C276Y	NM_000546
2015-051	CCND3	6	41903779	G	A	0.151	Q179*	NM_001136017
2015-051	CEBPA	19	33793203	CG	C	0.440	P39fs	NM_004364
2015-051	GATA2	3	128202767	G	A	0.474	A318V	NM_001145661
2015-051	KIT	4	55599340	T	A	0.061	N818K	NM_001093772
2015-051	NRAS	1	115256529	T	C	0.190	Q61R	NM_002524
2015-051	NRAS	1	115258747	C	T	0.102	G12D	NM_002524
2015-052	DNMT3A	2	25468165	AG	A	0.483	L504fs	NM_022552
2015-052	IDH2	15	90631934	C	T	0.468	R140Q	NM_002168
2015-052	NPM1	5	170837544	T	TCTGC	0.308	L258fs	NM_002520
2015-052	SRSF2	17	74732960	G	C	0.435	P95A	NM_001195427
2015-053	CEBPA	19	33792362	T	TCGG	0.208	-320P	NM_004364
2015-054	ASXL1	20	31024450	C	T	0.355	A1312V	NM_015338
2015-054	CTCF	16	67654615	C	T	0.505	R368C	NM_006565
2015-054	KDM6A	X	44879974	A	T	0.524	K188M	NM_021140
2015-054	PHF6	X	133547940	C	T	0.494	R225*	NM_001015877
2015-054	TET2	4	106156748	GAGAT	G	0.499	D551fs	NM_001127208
2015-054	TET2	4	106156747	C	T	0.501	R550*	NM_001127208
2015-055	ASXL1	20	31022441	A	AG	0.215	G643fs	NM_015338
2015-055	EZH2	7	148511065	G	A	0.516	R557W	NM_001203249
2015-055	IDH2	15	90631838	C	T	0.191	R172K	NM_002168
2015-055	NF1	17	29588751	C	T	0.086	R1513*	NM_000267
2015-055	SRSF2	17	74732935	CGGCGGCTGTGGTGTGAGTCCGGGG	C	0.495	PPDSHHSRR95R	NM_001195427
2015-055	TET2	4	106197287	G	C	0.600	E1874Q	NM_001127208
2015-056	DDX1	2	15737544	A	G	0.490	D70N	NM_004939
2015-056	KRAS	12	25398284	G	C	0.410	G12A	NM_033360
2015-056	RUNX1	21	36231792	T	C	0.460	D198N	NM_001754
2015-057	JAK2	9	5073770	G	T	0.999	V617F	NM_004972
2015-057	TP53	17	7578266	T	A	0.401	I195F	NM_000546
2015-059	DNMT3A	2	25457242	C	T	0.474	R882H	NM_022552
2015-059	FLT3	13	28608262	T	TTCATATTCTCTGAAATCAACGTAG	0.943	E598ATLISENMK	NM_004119
2015-059	NPM1	5	170837545	C	CTGTT	0.468	L258fs	NM_002520
2015-060	ASXL1	20	31023388	C	G	0.163	S958*	NM_015338
2015-060	BCOR	X	39932183	C	CGGTCCCA	0.360	V806fs	NM_001123383
2015-060	IDH1	2	209113113	G	A	0.208	R132C	NM_005896
2015-060	PHF6	X	133559270	TGAA	T	0.467	E337-	NM_001015877
2015-060	RUNX1	21	36252866	G	A	0.174	R139*	NM_001001890
2015-060	RUNX1	21	36259173	C	G	0.224	W106C	NM_001754
2015-061	IDH1	2	209113112	C	T	0.371	R132H	NM_005896
2015-061	NPM1	5	170837543	C	CTCTG	0.291	L258fs	NM_002520
2015-061	NRAS	1	115258747	C	G	0.378	G12A	NM_002524
2015-061	SMC1A	X	53441941	C	T	0.857	R96H	NM_006306
2015-062	DNMT3A	2	25463289	T	C	0.409	Y735C	NM_022552
2015-062	FLT3	13	28602376	C	T	0.188	M664I	NM_004119
2015-062	NPM1	5	170837543	C	CTCTG	0.346	L258fs	NM_002520
2015-062	PTPN11	12	112926885	C	T	0.157	S502L	NM_002834

AA: amino-acid sequence

Supplementary Table 4 | list of somatic variants detected by exome sequencing

UPN	Chr.	Position	Gene	refseq	Protein variation	Variation type	Freq variant in tumor (%)	Depth used	Genotype	Base_ref	rs	EVS Variant Freq (%)	1000G Variant Freq (%)	Sanger sequencing confirmation
2013-001	1	115256528	*NRAS*	NM_002524	Q61H	missense	47	73	A	T				yes
2013-001	1	245019780	*HNRNPU*	NM_004501<br>NM_031844	E611fs	FRAMESHIFT	22	67	GGGGAAAG<br>CCGGGA				0	yes
2013-001	3	38103776	*DLEC1*	NM_007335<br>NM_007337	E264K<br>E264K	missense	44	124	A	G	rs116202356	1,63	1	
2013-001	3	108117621	*MYH15*	NM_014981	A1686T	missense	33	33	T	C				
2013-001	3	185826264	*ETV5*	NM_004454		5-UTR	53	53	G	C				
2013-001	5	54518098	*MCIDAS*	NM_001190787	R171H	missense	55	22	T	C				
2013-001	6	70098604	*BAI3*	NM_001704	A1464T	missense	40	135	A	G				
2013-001	8	101717195	*PABPC1*	NM_002568	L593V	missense	17	24	C	G	rs113574896			
2013-001	8	101717220	*PABPC1*	NM_002568	M584I	missense	17	29	T	C	rs112868101			
2013-001	10	101572813	*ABCC2*	NM_000392	V669A	missense	36	63	C	T				yes
2013-001	10	135084295	*ADAM8*	NM_001164490<br>NM_001109<br>NM_001164489	E488K<br>E527K<br>E527K	missense	48	73	T	C		0		
2013-001	11	67798356	*NDUFS8*	NM_002496		5-UTR	23	13	C	A	rs117359991			
2013-001	12	53298504	*KRT8*	NM_002273<br>NM_001256293<br>NM_001256282	R88C<br>R88C<br>R116C	missense	25	12	A	G				
2013-001	14	24608199	*EMC9*	NM_016049		3-UTR	52	21	T	C		0		
2013-001	14	105415607	*AHNAK2*	NM_138420	V2061L	missense	20	41	G	C	rs112699389	0,07	1	
2013-001	16	31470913	*ARMC5*	NM_024742<br>NM_001288767<br>NM_001105247	A23V<br>A118V<br>A23V	missense	44	46	T	C				
2013-001	17	38975149	*KRT10*	NM_000421	S546R	missense	29	17	T	G				
2013-001	17	45234707	*CDC27*	NM_001256<br>NM_001114091	L173F<br>L173F	missense	12	40	A	T	rs75353677			
2013-001	19	5229617	*PTPRS*	NM_130854<br>NM_002850	A732E<br>A745E	missense	21	14	T	G	rs1064295			
2013-001	X	135958704	*RBMX*	NM_002139	P167A	missense	21	14	C	G	rs112089728			
2014-003	2	85570767	*RETSAT*	NM_017750	Y562fs	FRAMESHIFT	13	75		AGAT			0	
2014-003	4	79343136	*FRAS1*	NM_025074<br>NM_001166133	M1554V<br>M1554V	missense	64	11	G	A				
2014-003	4	185615925	*CENPU*	NM_024629		3-UTR	32	53		CAAGAGT			0	
2014-003	4	185615925	*PRIMPOL*	NM_152683	Q559fs	FRAMESHIFT	32	53		CAAGAGT			0	
2014-003	5	837488	*ZDHHC11*	NM_024786	V298F	missense	77	13	A	C	rs28461719			
2014-003	6	167592571	*TCP10L2*	NM_001145121	A244T	missense	14	55	A	G	rs28690444		4	
2014-003	8	49642389	*EFCAB1*	NM_001142857<br>NM_024593	S69T<br>S121T	missense	61	46	T	A				
2014-003	8	80915262	*MRPS28*	NM_014018	V123I	missense	40	107	T	C				
2014-003	8	145577021	*TMEM249*	NM_001252404<br>NM_001252402<br>NM_001280561	Q200H<br>S232I<br>S232I	missense	33	21	A	C				
2014-003	9	77354770	*TRPM6*	NM_001177310<br>NM_001177311<br>NM_017662	V1781I<br>V1781I<br>V1786I	missense	41	71	T	C				
2014-003	10	70646119	*STOX1*	NM_152709<br>NM_001130161<br>NM_001130162	D856V<br>D856V<br>D746V	missense	48	173	T	A				
2014-003	11	1651615	*KRTAP5-5*	NM_001001480	Y182C	missense	15	33	G	A	rs74396270			
2014-003	11	110050621	*RDX*	NM_001260493		3-UTR	38	8	T	C				
2014-003	13	28602329	*FLT3*	NM_004119	A680V	missense	15	47	A	G		0,01		yes
2014-003	16	58075547	*MMP15*	NM_002428	T313P	missense	31	13	C	A				
2014-003	17	39262166	*KRTAP4-9*	NM_001146041	V176L	missense	13	39	C	G	rs75622986			
2014-003	17	45247397	*CDC27*	NM_001114091<br>NM_001256	G88E<br>G88E	missense	42	41	T	C				yes
2014-003	18	9522393	*RALBP1*	NM_006788	C313fs	FRAMESHIFT	38	80	T				0	yes
2014-003	19	50412220	*IL4I1*	NM_001258017<br>NM_172374<br>NM_001258018		5-UTR	50	12	C	G				
2014-003	19	50412220	*NUP62*	NM_153718<br>NM_016553<br>NM_012346<br>NM_001193357<br>NM_153719	T282S<br>T282S<br>T282S<br>T282S<br>T282S	missense	50	12	C	G				
2014-003	19	55344235	*KIR2DS4*	NM_001281971<br>NM_001281972<br>NM_012314	I6V<br>I6V<br>I6V	missense	20	41	G	A	rs113410873			
2014-003	20	10625880	*JAG1*	NM_000214	T713M	missense	38	91	A	G				yes
2014-003	22	45404497	*PHF21B*	NM_001284296		5-UTR	43	7	A	G	rs77409377	3,19	4	
2014-003	X	140993864	*MAGEC1*	NM_005462	F225S	missense	17	23	C	T	rs34836042			
2013-004	1	40253868	*BMP8B*	NM_001720	R97Q	missense	20	15	T	C		0,81	4	
2013-004	1	145209119	*NOTCH2NL*	NM_203458		5-UTR	42	12	C	G				
2013-004	1	161072096	*PFDN2*	NM_012394	M49V	missense	45	130	C	T				
2013-004	2	216977841	*XRCC5*	NM_021141	V42I	missense	43	90	A	G				yes
2013-004	4	85993	*ZNF595*	NM_001286053<br>NM_001286052<br>NM_001286054<br>NM_182524	P199fs	FRAMESHIFT	25	20	TACAAATGT<br>GAAAAATGT<br>GGCAAAGC<br>CTTTAATAG<br>GTCCACATC<br>ACTTAGTAA<br>ACATAAGAG<br>AATTC				0	

UPN	Chr.	Position	Gene	refseq	Protein variation	Variation type	Freq variant in tumor (%)	Depth used	Genotype	Base_ref	rs	EVS Variant Freq (%)	1000G Variant Freq (%)	Sanger sequencing confirmation
2013-004	4	996204	*IDUA*	NM_000203	T374P	missense	23	13	C	A				
2013-004	4	7735055	*SORCS2*	NM_020777	A1039P	missense	18	17	C	G				
2013-004	7	23871924	*STK31*	NM_001260505<br>NM_001260504<br>NM_032944<br>NM_031414	T977M<br>T977M<br>T977M<br>T1000M	missense	27	26	T	C	rs55794023	0,09		
2013-004	7	99526894	*GJC3*	NM_181538	Q117L	missense	37	94	A	T				
2013-004	7	131241055	*PODXL*	NM_001018111<br>NM_005397	S22P<br>S22P	missense	31	13	G	A	rs117510299			
2013-004	7	143096444	*EPHA1*	NM_005232	Q300*	nonsense	83	12	A	G				
2013-004	10	55626415	*PCDH15*	NM_001142765<br>NM_001142766<br>NM_001142771<br>NM_001142773<br>NM_001142772<br>NM_001142763<br>NM_001142767<br>NM_001142764<br>NM_033056<br>NM_001142768<br>NM_001142769<br>NM_001142770	K1164R<br>K1235R<br>K1240R<br>K1213R<br>K1235R<br>K1240R<br>K1198R<br>K1235R<br>K1235R<br>K1213R<br>K1247R<br>K1235R	missense	50	88	C	T				
2013-004	11	59480952	*OR10V1*	NM_001005324	Q123*	nonsense	24	63	A	G	rs499037	1,26	5	
2013-004	11	65414096	*SIPA1*	NM_006747<br>NM_153253	T531P<br>T531P	missense	20	15	C	A				
2013-004	11	92714834	*MTNR1B*	NM_005959	H149Y	missense	48	214	T	C				
2013-004	11	117109543	*RNF214*	NM_001077239<br>NM_207343	V112M<br>V112M	missense	46	110	A	G				
2013-004	12	25398281	*KRAS*	NM_033360<br>NM_004985	G13D<br>G13D	missense	40	37	T	C	rs112445441			yes
2013-004	12	47630036	*PCED1B*	NM_001281429<br>NM_138371	H397R<br>H397R	missense	48	166	G	A				
2013-004	12	57114838	*NACA*	NM_001113203	T159N	missense	42	117	T	G				
2013-004	14	50623814	*SOS2*	NM_006939	T654A	missense	49	86	C	T				yes
2013-004	16	30485552	*ITGAL*	NM_001114380<br>NM_002209	A33T<br>A33T	missense	49	41	A	G				
2013-004	16	72821638	*ZFHX3*	NM_006885<br>NM_001164766	S3513G<br>S2599G	missense	50	6	C	T			1	
2013-004	19	16045064	*CYP4F11*	NM_001128932<br>NM_021187	P52L<br>P52L	missense	48	56	A	G				
2013-004	20	31022573	*ASXL1*	NM_015338	K886fs	FRAMESHIFT	43	96		GT			0	yes
2014-019	3	62478027	*CADPS*	NM_003716<br>NM_183394<br>NM_183393	P941H<br>P951H<br>P911H	missense	41	140	T	G				
2014-019	3	195451841	*ENSG00000176945*	ENST00000320736<br>ENST00000447234<br>NM_001282506<br>ENST00000436408<br>ENST00000445522	G123R<br>G123R<br>G123R<br>G123R<br>G88R	missense	41	17	A	G				
2014-019	3	195512143	*MUC4*	NM_018406	S2103F	missense	26	23	A	G				
2014-019	5	41805770	*OXCT1*	NM_000436	R285Q	missense	40	98	T	C				yes
2014-019	6	30994862	*MUC22*	NM_001198815	G552fs	FRAMESHIFT	18	133		GGCTCTGAGACCACTATGGCC<br>TCTACCATAGGCCCTGAGACC<br>ACCAAGGTCTCCACTGCAAGC<br>TCTGAGGTGACCACAGTCTTT<br>GCTGCAGGCTCTGAGACAATC<br>AGAGCCTCTACCGTAGGCTCT<br>GAGACCACCACAGTCTCTACC<br>ACAGGCTCTGAGACCACCACA<br>GCCTCCATCATGGGCTCTGAG			0	
2014-019	6	32557536	*HLA-DRB1*	NM_001243965<br>NM_002124		5-UTR	16	19	A	G	rs17211071			
2014-019	6	36688986	*RAB44*	NM_001257357	P359A	missense	17	24	G	C				
2014-019	7	44161487	*POLD2*	NM_001256879<br>NM_006230<br>NM_001127218	T56P<br>T91P<br>T56P	missense	21	19	G	T				
2014-019	9	95618618	*ZNF484*	NM_001007101<br>NM_001261459<br>NM_001261460		5-UTR	22	18	A	C	rs66503633			
2014-019	9	95618619	*ZNF484*	NM_001261460<br>NM_001007101<br>NM_001261459		5-UTR	17	18	C	A				
2014-019	11	5632402	*TRIM6*	NM_058166<br>NM_001198645<br>NM_001198644<br>NM_001003818	V433I<br>V258I<br>V258I<br>V461I	missense	37	142	A	G				
2014-019	11	47377080	*SPI1*	NM_003120<br>NM_001080547	R171S<br>R172S	missense	39	46	T	G				yes
2014-019	12	6640736	*NCAPD2*	NM_014865		3-UTR	50	6	T	C				
2014-019	12	22065906	*ABCC9*	NM_020297<br>NM_005691	R304H<br>R304H	missense	14	73	T	C				
2014-019	19	46242936	*ENSG0000023745*	ENST00000457052	E42K	missense	46	102	A	G				
2014-019	19	54976259	*CDC42EP5*	NM_145057		3-UTR	50	10	A	G	rs113630689	0,02	1	
2014-019	21	45959819	*KRTAP10-1*	NM_198691	T72I	missense	18	17	A	G				
2015-056	1	220142184	*EPRS*	NM_004446.2	P1501fs	FRAMESHIFT	42	136	AG	A				
2015-056	6	142737163	*GPR126*	NM_198569.2	I967T	missense	53	65	T	C				
2015-056	11	33565657	*KIAA1549L*	NM_012194.2	G553R	missense	45	380	G	A				
2015-056	12	25398284	*KRAS*	NM_033360.3	G12A	missense	41	87	C	G	rs121913529			
2015-056	16	71976871	*PKD1L3*	NM_181536.1	P1304fs	FRAMESHIFT	46	160	TG	T				
2015-056	2	28739693	*PLB1*	NM_153021.4	T25I	missense	44	158	C	T				
2015-056	21	36231792	*RUNX1*	NM_001754.4	D198N	missense	46	274	C	T				
2015-056	10	105561071	*SH3PXD2A*	NM_014631.2	Y41C	missense	36	52	T	C				
2015-056	2	138033576	*THSD7B*	NM_001080427.1	G796V	missense	43	101	G	T				
2015-056	2	15737544	*DDX1*	NM_004939.2	D70N	missense	49	63	G	A				

UPN	Chr.	Position	Gene	refseq	Protein variation	Variation type	Freq variant in tumor (%)	Depth used	Genotype	Base_ref	rs	EVS Variant Freq (%)	1000G Variant Freq (%)	Sanger sequencing confirmation
2014-009	2	17898117	*SMC6*	NM_024624<br>NM_001142286	L413I<br>L413I	missense	11	91	T	A				
2014-009	2	69472439	*ANTXR1*	NM_032208	P506Q	missense	7	132	A	C		0.01		
2014-009	2	175289311	*SCRN3*	NM_001193528<br>NM_024583	K335N<br>K342N	missense	8	66	T	G				
2014-009	2	241622273	*AQP12B*	NM_001102467		5-UTR	22	78	G	A	rs114235449			
2014-009	2	241622274	*AQP12B*	NM_001102467		5-UTR	21	61	A	T	rs114613033			
2014-009	3	23959970	*RPL15*	NM_002948<br>NM_001253382<br>NM_001253380<br>NM_001253383<br>NM_001253379<br>NM_001253384	R71L<br>R71L<br>R71L<br>R71L<br>R71L<br>R71L	missense	20	60	T	G				
2014-009	4	367413	*ENSG00000131127*	ENST00000240499<br>NM_003441	K396T<br>K396T	missense	10	226	C	A				
2014-009	4	367452	*ENSG00000131127*	ENST00000240499<br>NM_003441	D409V<br>D409V	missense	9	226	T	A	rs75820398			
2014-009	6	32552017	*HLA-DRB1*	NM_001243965<br>NM_002124	D71E<br>T80R	missense	9	93	C	G	rs1059582			
2014-009	7	104753095	*KMT2E mll5*	NM_018682<br>NM_182931	P1631Q<br>P1631Q	missense	12	54	A	C				
2014-009	9	2060847	*SMARCA2*	NM_001289396<br>NM_001289397<br>NM_003070<br>NM_139045	I518T<br>I518T<br>I518T<br>I518T	missense	8	530	C	T				
2014-009	9	2060862	*SMARCA2*	NM_139045<br>NM_001289397<br>NM_001289396<br>NM_003070	D523V<br>D523V<br>D523V<br>D523V	missense	9	575	T	A				
2014-009	9	2088516	*SMARCA2*	NM_001289396<br>NM_001289397<br>NM_139045<br>NM_003070	E929G<br>E871G<br>E929G<br>E929G	missense	9	211	G	A				
2014-009	11	124669789	*MSANTD2*	NM_001301087		5-UTR	8	138	T	G				
2014-009	11	124669789	*MSANTD2*	NM_024631	S163Y	missense	8	138	T	G				
2014-009	12	53344140	*KRT18*	NM_199187<br>NM_000224	R149H<br>R149H	missense	10	81	A	G				
2014-009	12	122097107	*MORN3*	NM_173855	D98G	missense	7	220	C	T				
2014-009	13	28592642	*FLT3*	NM_004119	D835Y	missense	9	223	A	C				yes
2014-009	16	53480967	*RBL2*	NM_005611	T196S	missense	6	234	T	A				
2014-009	17	7758934	*TMEM88*	NM_203411	R128*	nonsense	12	280	T	C				
2014-009	19	12542411	*ZNF443*	NM_005815	R192H	missense	23	132	T	C				
2014-009	19	19010494	*COPE*	NM_199442<br>NM_007263<br>NM_199444	S256R<br>S307R<br>S255R	missense	13	51	T	G		0	0.05	
2014-009	19	44117475	*SRRM5*	NM_001145641	H401R	missense	9	126	G	A	rs118016293			
2014-009	2	198267359	*SF3B1*	NM_012433	K666N	missense	43	199	A	C				yes
2014-009	12	112915455	*PTPN11*	NM_002834	F285S	misense	50	96	C	T				yes
2014-018	1	46073470	*NASP*	NM_001195193<br>NM_002482	E232A<br>E296A	missense	10	101	C	A				
2014-018	3	10085280	*FANCD2*	NM_001018115<br>NM_033084		intron+splice<br>intron	16	57	G	A	rs664233			
2014-018	5	176939370	*DDX41*	NM_016222	R525H	missense	32	302	T	C				yes
2014-018	6	32057104	*TNXB*	NM_019105	A804G	missense	25	310	C	G				
2014-018	6	110948334	*CDK19*	NM_001300964<br>NM_015076<br>NM_001300960<br>NM_001300963	G161S<br>G221S<br>G177S<br>G161S	missense	27	89	T	C				
2014-018	9	134358743	*PRRC2B*	NM_013318		intron+splice<br>intron	12	120	T	C				
2014-018	10	7285668	*SFMBT2*	NM_001029880<br>NM_001018039		intron+splice<br>intron	9	77	A	C				
2014-018	11	67270150	*PITPNM1*	NM_001130848<br>NM_004910	V40M<br>V40M	missense	13	241	T	C				
2014-018	11	113813821	*HTR3B*	NM_006028	V272L	missense	27	478	T	G				
2014-018	12	102122910	*SYCP3*	NM_153694<br>NM_001177949<br>NM_001177948	Q212K<br>Q212K<br>Q212K	missense	11	47	T	G				
2014-018	17	64225437	*APOH*	NM_000042	R21W	missense	13	223	A	G		0.02		
2014-018	17	74732956	*MFSD11*	NM_001242534		5-UTR	24	193	A	G				
2014-018	17	74732956	*SRSF2*	NM_001195427<br>NM_003016	P96L<br>P96L	missense	24	193	A	G				yes
2014-018	19	11594482	*ZNF653*	NM_138783		3-UTR	25	150	G	C				
2014-018	19	45448707	*APOC4*	NM_001646		3-UTR	11	65	A	G				
2014-018	19	53303025	*ZNF28*	NM_006969	Y691*	nonsense	8	96	T	G	rs111678182			
2014-018	19	56422023	*NLRP13*	NM_176810	E730K	missense	14	177	T	C				
2014-018	X	70775916	*OGT*	NM_181672<br>NM_181673	A346V<br>A336V	missense	10	215<br>345	T	C				
2014-018	X	154305490	*BRCC3*	NM_001242640<br>NM_024332<br>NM_001018055	R82*<br>R81*<br>R81*	nonsense	32	57<br>123	T	C				

Supplementary Table 5 | Classification of chromosomal and genetic events in four categories

Lesions in epigenetic regulators	Mutations in NPM1, transcription factors, and splicing factors	Proliferative mutations	Other events
ASXL1	*CEBPA*	*BCLAF1*	*ABCC2*
BCOR	CDK19	*CBL*	*ATM*
BCORL1	*DDX41*	*CCND3*	*BRCC3*
CTCF	*ETV6*	*CDC27*	*DDX1*
CUX1	*GATA2*	*CSF3R*	*FBXW7*
DNMT3A	*HNRNPU*	*DSCAM*	*JAG1*
EZH2	*IKZF1*	*FLT3*	*NOTCH1*
IDH1	*NPM1*	*JAK1*	*NUMA1*
IDH2	*RUNX1*	*JAK2*	*OXCT1*
KDM6A	*SF3B1*	*KIT*	*PDS5B*
MLL	*SPI1*	*KRAS*	*PHF6*
MLL2	*SRSF2*	*NF1*	*RB1*
NSD1	*U2AF1*	*NRAS*	*SMC1A*
OGT	*WT1*	*PTPN11*	*STAG2*
SETBP1	*ZRSR2*	*RALBP1*	*TP53*
TET2		*RET*	*XRCC5*
TET3		*SH2B2*	Any other cytogenetic abnormality
del(20q)		*SOS2*	
t(11;v) involving MLL			
inv(16)			
t(8;21)			

Supplementary Table 6 | List of somatic variants detected with the targeted resequencing panel at relapse. The list of all detected variants is in **Supplementary Data Set 2** (Excel file).

patient	gene	chromosome	loc	ref base	altered base	VAF	AA	NM
2013-002	ASXL1	20	31021118	C	T	0.398	Q373*	NM_015338
2013-002	EZH2	7	148512036	A	C	0.410	C504G	NM_152998
2013-002	EZH2	7	148523591	G	A	0.449	R249*	NM_152998
2013-002	JAK2	9	5073770	G	T	0.095	V617F	NM_004972
2013-002	SETBP1	18	42531913	G	A	0.065	G870S	NM_015559
2013-006	CCND3	6	41903745	C	CG	49.738	R271fs	NM_001136017
2013-006	WT1	11	32417941	C	CACTTTTT	38.451	R141fs	NM_000378
2014-001	DNMT3A	2	25457242	C	T	0.020	R882H	NM_022552
2014-001	NPM1	5	170837543	C	CTCTG	0.024	L258fs	NM_002520
2014-001	TET2	4	106157554	TATAGTCAGACC	T	0.047	Y819fs	NM_001127208
2014-001	TET2	4	106180853	A	G	0.063	Y1294C	NM_001127208
2014-009	PTPN11	12	112915455	T	C	0.125	F285S	NM_002834
2014-009	SF3B1	2	198267359	C	A	0.063	K666N	NM_012433
2014-015	RET	10	43612093	G	A	0.090	G733D	NM_020630
2014-015	U2AF1	21	44524456	G	A	0.448	S34F	NM_001025203
2014-016	DNMT3A	2	25463175	A	T	0.174	L773H	NM_022552
2014-016	FLT3	13	28622454	TTTCGAGAGAAGG	T	0.112	TFSRK384K	NM_004119
2014-016	JAK1	1	65301882	A	C	0.098	L1053V	NM_002227
2014-016	NPM1	5	170837544	T	TCTGC	0.164	L258fs	NM_002520
2014-016	TET2	4	106164088	A	G	0.119	splice site	NM_001127208
2014-017	ATM	11	108186629	C	G	0.114	P2029R	NM_000051
2014-017	DNMT3A	2	25463287	G	A	0.101	R736C	NM_022552
2014-017	IDH1	2	209113113	G	A	0.125	R132C	NM_005896
2014-021	TET2	4	106157554	TATAGTCAGACC	T	0.130	Y819fs	NM_001127208
2014-027	DNMT3A	2	25468153	A	G	0.299	L508P	NM_022552
2014-027	IDH1	2	209113112	C	T	0.381	R132H	NM_005896
2014-027	TP53	17	7578190	T	C	0.336	Y220C	NM_000546
2014-027	TP53	17	7578454	G	A	0.317	A159V	NM_000546
2014-029	ASXL1	20	31022402	TCACCACTGCCATAGAGAGGCGGC	T	0.229	H630fs	NM_015338
2014-029	DNMT3A	2	25457242	C	T	0.237	R882H	NM_022552
2014-029	IDH1	2	209113113	G	A	0.098	R132C	NM_005896
2014-029	IDH2	15	90631934	C	T	0.121	R140Q	NM_002168
2014-029	RUNX1	21	36164838	C	CG	0.104	R319fs	NM_001001890
2014-029	SRSF2	17	74732959	G	T	0.210	P95T	NM_001195427
2014-033	DNMT3A	2	25457243	G	A	0.120	R882C	NM_022552
2014-033	RUNX1	21	36164638	A	AG	0.067	S386fs	NM_001001890
2014-033	TET2	4	106158509	G	A	0.072	G1173D	NM_001127208
2014-037	ASXL1	20	31022402	TCACCACTGCCATAGAGAGGCGGC	T	0.406	H630fs	NM_015338
2014-037	BCOR	X	39932171	G	A	0.387	R810*	NM_001123383
2014-037	BCORL1	X	129155104	C	T	0.311	R1196*	NM_021946
2014-037	BCORL1	X	129162789	C	T	0.094	R1420*	NM_021946
2014-037	CEBPA	19	33793191	CG	C	0.368	P43fs	NM_004364
2014-037	EZH2	7	148506458	T	A	0.548	K629M	NM_001203249
2014-037	FLT3	13	28608242	A	AACTCCCATTTGAGATCAT	0.288	-605MISNGS	NM_004119
2014-037	RUNX1	21	36252866	G	GTGTC	0.177	V137fs	NM_001001890
2014-037	TET2	4	106196480	G	T	0.420	A1605S	NM_001127208
2014-037	WT1	11	32421575	G	C	0.131	Y110*	NM_001198552
2014-038	FLT3	13	28608251	T	TTGAGATCATATTCATATTCTC	0.302	-602ENMNMIS	NM_004119
2014-038	NRAS	1	115256530	G	T	0.100	Q61K	NM_002524
2014-038	PTPN11	12	112888198	G	A	0.057	A72T	NM_002834
2014-039	DNMT3A	2	25457242	C	T	0.449	R882H	NM_022552
2014-039	MLL2	12	49433247	G	A	0.061	R2734*	NM_003482
2014-039	NPM1	5	170837543	C	CTCTG	0.051	L258fs	NM_002520
2014-039	TET2	4	106164913	C	T	0.857	R1261C	NM_001127208
2014-039	ZRSR2	X	15822319	A	T	0.163	E133V	NM_005089
2014-042	CCND3	6	41903698	CATCT	C	0.437	T285fs	NM_001136017
2014-042	WT1	11	32417924	C	CGGGGCTG	0.367	P148fs	NM_000378
2014-043	CTCF	16	67654643	G	A	0.471	R377H	NM_006565
2014-043	DNMT3A	2	25469541	C	T	0.469	W409*	NM_022552
2014-043	NF1	17	29527448	TC	T	0.495	L300fs	NM_000267
2014-043	NPM1	5	170837545	C	CTGTA	0.513	W259fs	NM_002520
2014-043	TET2	4	106197002	G	GC	0.502	A1779fs	NM_001127208
2014-043	TET2	4	106190795	G	A	0.446	C1358Y	NM_001127208
2015-003	ASXL1	20	31022441	A	AG	0.241	G643fs	NM_015338
2015-003	CBL	11	119148919	T	C	0.944	L380P	NM_005188
2015-003	FLT3	13	28592641	T	A	0.073	D835V	NM_004119
2015-003	FLT3	13	28610160	C	G	0.105	E444Q	NM_004119
2015-003	IKZF1	7	50450385	G	C	0.366	G103A	NM_001220766
2015-003	JAK1	1	65349155	G	C	0.449	L4V	NM_002227
2015-003	SETBP1	18	42531913	G	A	0.469	G870S	NM_015559
2015-003	TET3	2	74273972	C	G	0.513	P175A	NM_144993
2015-003	WT1	11	32417947	G	A	0.615	R140*	NM_001198552
2015-004	DNMT3A	2	25463536	C	T	0.382	V716I	NM_022552
2015-004	IDH1	2	209113113	G	T	0.320	R132S	NM_005896
2015-004	MLL	11	118375166	G	A	0.073	M2850I	NM_005933
2015-004	RUNX1	21	36252877	C	T	0.118	R135K	NM_001001890
2015-005	ASXL1	20	31023159	C	T	0.418	Q882*	NM_015338
2015-005	DNMT3A	2	25457242	C	T	0.458	R882H	NM_022552
2015-005	PTPN11	12	112888199	C	T	0.417	A72V	NM_002834
2015-005	RUNX1	21	36231783	G	A	0.000	R174*	NM_001001890
2015-005	SETBP1	18	42531907	G	A	0.407	D868N	NM_015559
2015-005	ZRSR2	X	15838412	G	A	0.447	V304M	NM_005089
2015-006	IDH2	15	90631934	C	T	0.508	R140Q	NM_002168
2015-006	NOTCH1	9	139397775	C	T	0.516	V1676I	NM_017617
2015-006	NPM1	5	170837543	C	CTCTG	0.515	L258fs	NM_002520
2015-006	SRSF2	17	74732959	G	T	0.489	P95T	NM_001195427
2015-014	DNMT3A	2	25467172	CCCA	C	0.327	VG567G	NM_022552
2015-014	IDH2	15	90631934	C	A	0.370	R140L	NM_002168
2015-014	NPM1	5	170837543	C	CTCTG	0.253	L258fs	NM_002520
2015-020	ASXL1	20	31022441	A	AG	0.211	G643fs	NM_015338
2015-020	BCOR	X	39932084	T	TG	0.969	K839fs	NM_001123383
2015-020	FLT3	13	28608255	G	GATCATATTCATATTCTCTGAA	0.456	D600DSENMNMI	NM_004119
2015-020	SRSF2	17	74732959	G	T	0.429	P95T	NM_001195427
2015-020	STAG2	X	123195170	G	T	0.980	E505*	NM_001042749
2015-047	TP53	17	7578394	T	C	0.512	H179R	NM_000546
2015-058	RUNX1	21	36206875	G	A	0.482	Q186*	NM_001001890
2015-058	SF3B1	2	198267705	C	T	0.480	E592K	NM_012433
2015-058	SMC1A	X	53432009	G	A	0.973	R711W	NM_006306

Supplementary Table 7 | Temporal evolution of AML clones at relapse

UPN	Delay to relapse (days)	Stable lesions	Gained lesions	Lost lesions	Quantitative changes (>5 fold) or switch in dominant clone
2014-033	162	*DNMT3A* p.R882C*, RUNX1* p.S386fs*,* *TET2* p.G1137D	*FLT3-ITD* 24bp	*FLT3-ITD* 21bp *FLT3-ITD* 78 bp	
2015-047	164	*TP53* p.H179R, del(5q), -7, -8, -17, -18, add(11p), add(5q), ?20, add(3q)	add(19q)		
2014-001	173	*TET2* p.Y1294C, *DNMT3A* p. R882H, *NPM1* p.L258fs, *TET2* p.L627fs			*NRAS* p.Q61K↘ *KRAS* p.Q61H↘
2014-017	175	*IDH1* p.R132C, *DNMT3A* p.R736C, *ATM* p.P2029R		*KDM6A* p.V455fs *NPM1* p. L258fs	
2014-016	240	*TET2 splice site, DNMT3A* p.L773H, *JAK1* p.L1053V, *NPM1* p. L258fs, *FLT3*-ITD 63bp,*FLT3*-LOH			
2014-039	253	*TET2* p.R1261C, *TET2*-LOH, *DNMT3A* p.R882H	*MLL2* p.R2734*, Complex Chr.X,4,8 rearrangement		*NPM1* p.L258fs ↘ *ZRSR2* p.E133V↗
2014-042	272	del(20q)		*WT1* p.A153fs	*WT1* p.P148fs↗ *CCND3* p.T285fs↗
2014-038	273	t(6;11), *FLT3*-ITD	*NRAS* p.Q61K, *PTPN11* p.A72T		
2014-027	287	*TP53* p.Y220C, *DNMT3A* p.L508P, *IDH1* p.R132H, *TP53* p.A159V, del(5q), -16, t(3;7)			
2014-029	321	*DNMT3A* p.R882H, *SRSF2* p.P95T *ASXL1* p.H630fs, *IDH1* p.R132C	*RUNX1* p.R319fs, t(1;6)		*IDH2* p.R140Q↗
2014-037	322	*TET2* p.A1605S, *EZH2* p.K629M *BCOR* p.R810*, *ASXL1* p.H630fs *CEBPA* p.43fs, *BCORL1* p.R1196* *RUNX1* p.V137fs, *FLT3*-ITD	*BCORL1* p.R1420*, t(6;19;16)	*PTPN11* p.Q510H	*WT1* p.Y510*↗
2014-015	322	del(20q), *U2AF1* p.S34F, *RET* p.G733D	t(1;3)		
2013-006	413	*CCND3* p.R271fs, *WT1* p.R141fs	*FLT3*-ITD 39bp, *FLT3*-ITD 66bp	*FLT3*-ITD 129bp	
2014-009	414	*PTPN11* p.F285S, *SF3B1* p.K666N, -7	del(7q)	*FLT3* p.D835Y	
2014-043	491	*DNMT3A* p.W409*, *TET2* p.A1779fs, *TET2* p.C1358Y, *CTCF* p.R377H, *NPM1* p.W259fs	*NF1* p. L300fs *FLT3*-ITD 45bp	*TET3* p.R899*	
2013-002	497	*ASXL1* p.Q373*, *EZH2* p.C504G, *EZH2* p. R249*	*SETBP1* p.G870S, *JAK2* p.V617F, *FLT3*-ITD, t(11;21)	*FLT3* p.D839E, *RUNX1* p.D66fs	
2014-021	1038	t(8;21), *TET2* p.Y819fs		*FLT3* p.Q580p, del(11p)	-7 ↗
2015-014	1966	*DNMT3A* p.VG567G	*IDH2* p.R140L, *NPM1* p.L258fs	*NPM1* p.L258fs, *SMC1A* p. R790W	
2015-006	2312	*IDH2* p.R140Q, *NOTCH1* p.V1676I, *SRSF2* p.95T, *NPM1* p.L258fs		+Y	
2015-005	2411	*DNMT3A* p.R882H, *ASXL1* p.Q882*	*SETBP1* p.D868N, *ZRSR2* p.V304M, *RUNX1* p.R174*, *PTPN11* p.A72V, -7	*DSCAM* p.E1836*, LOH chr(21) *RUNX1* p.A33fs, *IDH1* p.R132C	
2015-003	3910	none	*CBL* p.L380P, *CBL* LOH, *ASXL1* p.G643fs, *TET3* p.P175A, *WT1* p.R140*, *WT1* LOH, *SETBP1* p.G870S, *JAK1* p.L4V, *IKZF1* p.G103A, *FLT3* p.D835V, *FLT3* p.E444Q, del(7p), del(7q),	*SF3B1* p.-705S, *SH2B2* p.A392G, *TP53* p.R273H, *NRAS* p.G13D, add(6), t(?X;8), del(6q)	
2015-004	3939	*DNMT3A* p.V716I	*MLL* p.M2850I, *IDH1* p.R132H, -7, *RUNX1* p.R135K	*IDH2* p.R172K, *BCOR* p.N575fs	

Supplementary Table 8 | deep sequencing analyses in twelve patients at diagnosis relapse or in complete remission at different steps of follow-up. Data show the numbers of amplicon families (see methods) with the known alterated bases, alternative (non alterated/non reference) bases, and the total number of amplicon families (amplicons).

UPN 2014-001						CR after one course				CR after two courses			
Gene	AA	Chr	Pos	Reference base	alterated base	n alterated bases	n alternative bases	amplicons	frequency	n alterated bases	n alternative bases	amplicons	frequency
TET2	Y1294C	4	106180853	A	G	25	10	11810	0.0021	165	6	18226	0.009053001
DNMT3A	R882H	2	25457242	C	T	14	1	6395	0.00218921	78	2	7913	0.009857197
NPM1	L258fs	5	170837543	C	CTCTG	11	na	3007	0.003658131	32	na	4460	0.007174888
KRAS	Q61H	12	25380275	T	G	16	6	6446	0.002482159	0	0	11309	0
TET2	L627fs	4	106156978	CT	C	8	na	7816	0.001023541	49	na	11538	0.004246837
NRAS	Q61K	1	115256530	G	T	1	5	5372	<0.001	0	6	9053	0

UPN 2014- 039						diagnosis (deep sequencing)				CR after one course				CR after two courses			
Gene	AA	Chr	Pos	reference base	alterated base	n alterated bases	n alternative bases	amplicons	frequency	n alterated bases	n alternative bases	amplicons	frequency	n alterated bases	n alternative bases	amplicons	frequency
TET2	R1261C	4	106164913	C	T	5942	5	6689	0.88	3382	3	6998	0.49	2183	26	4548	0.478
NPM1	L258fs	5	170837544	C	CTCTG	1327		3545	0.374	8	na	3586	0.0022	0	na	2296	0
DNMT3A	R882H	2	25457242	C	T	2346	9	6572	0.356	1649	5	6389	0.258	857	5	3799	0.226
ZRSR2	E133V	X	15822319	A	T	21	2	570	0.037	10	1	527	0.019	13	4	468	0.028

UPN 2014-029						diagnosis (deep sequencing)				CR after one course			
Gene	AA	Chr	Pos	reference base	alterated base	n alterated bases	n alternative bases	amplicons	frequency	n alterated bases	n alternative bases	amplicons	frequency
DNMT3A	R882H	2	25457242	C	T	2424	1	7092	0.342	1825	1	7249	0.252
IDH2	R140Q	15	90631934	C	T	26	5	10278	0.0025	12	11	9596	0.0012
ASXL1	H630fs	20	31022403	TCACCACTGCCATAGAGAGGCGGC	T	1842	na	7703	0.239	1140	na	6727	0.169
IDH1	R132C	2	209113113	G	A	2800	5	6905	0.405	1939	7	6465	0.3
RUNX1	R319fs	21	36164838	C	CG	159	na	12724	0.0124	1049	na	12910	0.081

UPN 2015-014						diagnosis (deep sequencing)				CR after one course			
Gene	AA	Chr	Pos	reference base	alterated base	n alterated bases	n alternative bases	amplicons	frequency	n alterated bases	n alternative bases	amplicons	frequency
DNMT3A	VG378G	2	25467172	CCCA	C	961	na	2038	0.4715	2	na	1630	0.00122
NPM1	L258fs	5	170837544	C	CTCTG	1412	na	2981	0.473	0	na	2102	0
SMC1A	R790W	X	53430550	G	A	2159	5	2447	0.882	0	18	2137	0
IDH2	R140L	15	90631934	C	A	8	10	9052	<0.001	0	8	5377	0

UPN 2015-014						CR after 3 courses				CR 8 months before relapse				relapse (deep sequencing)			
Gene	AA	Chr	Pos	reference base	alterated base	n alterated bases	n alternative bases	amplicons	frequency	n alterated bases	n alternative bases	amplicons	frequency	n alterated bases	n alternative bases	amplicons	frequency
DNMT3A	VG378G	2	25467172	CCCA	C	6	na	2026	0.0029	259	na	1404	0.1844	1772	na	4527	0.3914
NPM1	L258fs	5	170837544	C	CTCTG	4	na	4011	<0.001	4	na	2890	0.001384083	1098	na	7415	0.1480782
SMC1A	R790W	X	53430550	G	A	3	8	3060	<0.001	1	10	2044	<0.001	8	2	7770	0.0010296
IDH2	R140L	15	90631934	C	A	0	7	8306	0	984	7	5156	0.191	5954	14	17704	0.336

UPN 2014-038						diagnosis (deep sequencing)				CR after one course			
Gene	AA	Chr	Pos	reference base	alterated base	n alterated bases	n alternative bases	amplicons	frequency	n alterated bases	n alternative bases	amplicons	frequency
FLT3	ITD	13	28608252	T	TTGAGATCATATTCATATTCTC	2998	na	11936	0.251	0	na	9409	0
NRAS	Q61K	1	115256530	G	T	1	2	8375	<0.001	0	0	7089	0
PTPN11	A72T	12	112888198	G	A	0	0	1440	0	0	0	1031	0

UPN 2014-037						CR after one course			
Gene	AA	Chr	Pos	reference base	alterated base	n alterated bases	n alternative bases	amplicons	frequency
ASXL1	H630fs	20	31022402	TCACCACTGCCATAGAGAGGCGGC	T	317	na	6014	0.052
BCOR	R810*	X	39932171	G	A	217	15	9854	0.021
BCORL1	R1196*	X	129155104	C	T	2	7	8976	<0.001
CEBPA	P43fs	19	33793191	CG	C	117	na	6924	0.016897747
EZH2	K629M	7	148506458	T	A	38	1	2417	0.016
FLT3	ITD	13	28608243	A	AACTCCCATTTGAGATCAT	0	na	7859	0
RUNX1	V137fs	21	36252871	G	GTGTC	5	na	5365	0.000931966
TET2	A1605S	4	106196480	G	T	733	2	3064	0.238
WT1	Y110*	11	32421575	G	C	0	3	6117	0
PTPN11	Q510H	12	112926910	G	T	147	4	7898	0.019
BCORL1	R1420*	X	129162789	C	T	0	8	9016	0

UPN 2014-017						CR after one course			
Gene	AA	Chr	Pos	reference base	altered base	n altered bases	n alternative bases	amplicons	frequency
DNMT3A	R736C	2	25463287	G	A	133	4	4275	0.031
ATM	P2029R	11	108186629	C	G	80	1	2163	0.037
IDH1	R132C	2	209113113	G	A	149	3	5499	0.027
NPM1	L258fs	5	170837544	C	CTCTG	0	na	3504	0
KDM6A	V455fs	X	44920601	TG	G	0	na	5601	0

UPN 2014-033						CR after one course			
Gene	AA	Chr	Pos	reference base	altered base	n altered bases	n alternative bases	amplicons	frequency
DNMT3A	R882C	2	25457243	G	A	608	5	5344	0.114
RUNX1	S386fs	21	36164639	A	AG	492	na	4193	0.117
TET2	G1137D	4	106158509	G	A	518	4	3749	0.138

UPN 2015-004						diagnosis (deep sequencing)				relapse (deep sequencing)			
Gene	AA	Chr	Pos	reference base	altered base	n altered bases	n alternative bases	amplicons	frequency	n altered bases	n alternative bases	amplicons	frequency
BCOR	N575fs	X	39932877	T	TG	3549	na	7582	0.468	0	na	7110	0
DNMT3A	V716I	2	25463536	C	T	5832	5	13851	0.421	4502	3	12200	0.369
IDH2	R172K	15	90631838	C	T	6182	19	14059	0.44	0	4	12985	0
IDH1	R132S	2	209113113	G	T	3	5	6666	<0.001	1856	2	5785	0.321
RUNX1	R135K	21	36252877	C	T	0	12	7597	0	471	2	6192	0.076

UPN 2015-005						diagnosis (deep sequencing)				relapse (deep sequencing)			
Gene	AA	Chr	Pos	reference base	altered base	n altered bases	n alternative bases	amplicons	frequency	n altered bases	n alternative bases	amplicons	frequency
ASXL1	Q882*	20	31023159	C	T	1498	6	5425	0.276	1720	1	4451	0.386
DNMT3A	R882H	2	25457242	C	T	2483	9	8115	0.305	2502	7	6902	0.36
PTPN11	A72V	12	112888199	C	T	0	2	1178	0	572	2	1234	0.462
RUNX1	A33fs	21	36259304	CGTCCGGG	C	0	na	15996	0	3569	na	11990	0.297
SETBP1	D868N	18	42531907	G	A	2	6	17543	<0.001	5832	6	14149	0.412
ZRSR2	V304M	X	15838412	G	A	0	4	9693	0	3588	5	8063	0.445
RUNX1	R174*	21	36231783	G	A	4986	4	10416	0.478	2	4	12101	< 0.001
IDH1	R132C	2	209113113	G	A	2104	3	7562	0.278	0	4	6934	0
DSCAM	E1836*	21	41414478	C	A	4899	1	8024	0.61	0	1	6500	0

UPN 2014-041						CR post one course				CR post two courses				CR post six courses			
Gene	AA	Chr	Pos	reference base	altered base	n altered bases	n alternative bases	amplicons	frequency	n altered bases	n alternative bases	amplicons	frequency	n altered bases	n alternative bases	amplicons	frequency
DNMT3A	R882C	2	25457243	G	A	3002	14	8014	0.373	994	6	4606	0.215	2083	6	6390	0.326
IDH1	R132H	2	209113112	C	T	0	3	7026	0	3	7	3197	< 0.001	0	5	4968	0
PTPN11	A72V	12	112888199	C	T	0	2	1440	0	0	1	600	0	0	0	855	0
NPM1	L258fs	5	170837544	C	CTCTG	0	na	3743	0	0	na	2368	0	0	na	3005	0
NRAS	Q61K	1	115256530	G	T	0	2	7937	0	0	3	3883	0	0	0	6070	0

UPN 2014-015						CR (after one course)			
Gene	AA	Chr	Pos	reference base	altered base	n altered bases	n alternative bases	amplicons	frequency
U2AF1	S34F	21	44524456	G	A	11	2	3612	0.0030
RET	G733D	10	43612093	G	A	12	1	4321	0.0028

na: not applicable (for indels, see methods)

Supplementary table 9 | Analysis of NSG bone marrow repopulation by primary AML cells.

UPN	Time from injection to analysis	MOUSE	hCD45%	CD45$^+$ CD33$^+$%	CD45$^+$ CD19$^+$%	CD45$^+$ CD3$^+$%
2014-002	28	mouse 1	0.1	0	0	0
		mouse 2	0.4	0	0	0
2015-007	25	mouse 1	0	0	0	0
2015-051	26	mouse 1	0	0	0	0
		mouse 2	0	0	0	0
		mouse 3	0	0	0	0
		mouse 4	0	0	0	0
		mouse 5	0	0	0	0
		mouse 6	0	0	0	0
		mouse 7	0	0	0	0
2015-058	20	mouse 1	0.6	0.15	0	0.48
		mouse 2	0	0	0	0
		mouse 3	0.5	0.25	0	0.25
		mouse 4	0	0	0	0
		mouse 5	0.03	0	0	0
		mouse 6	0	0	0	0
2015-048	14	mouse 1	0.3	0.29	0	0
		mouse 2	0.06	0.05	0	0
		mouse 3	0.02	0.02	0	0
		mouse 4	0.2	0.2	0	0
		mouse 5	0	0	0	0
2014-003	13	mouse 1	0	0	0	0
		mouse 2	0	0	0	0
		mouse 3	0	0	0	0
2015-020	19	mouse 1	0	0	0	0
		mouse 2	0	0	0	0
		mouse 3	0	0	0	0
2015-050	16	mouse 1	0	0	0	0
		mouse 2	0	0	0	0
		mouse 3	0	0	0	0
		mouse 4	0	0	0	0
		mouse 5	0	0	0	0
2015-019	19	mouse 1	0	0	0	0
		mouse 2	0	0	0	0
		mouse 3	0	0	0	0
2015-004	19	mouse 1	0	0	0	0
		mouse 2	0	0	0	0
		mouse 3	0	0	0	0
2015-054	22	mouse 1	0	0	0	0
		mouse 2	0	0	0	0
		mouse 3	0.3	0.3	0	0
2015-053	30	mouse 1	0	0	0	0
		mouse 2	0	0	0	0
		mouse 3	0	0	0	0
		mouse 4	2	0.12	1.8	0
		mouse 5	0.12	0	0.11	0.01
		mouse 6	1.2	0.12	1.08	0
2014-040	12	mouse 1	1	1	0	0
		mouse 2	0.5	0.5	0	0
		mouse 3	2	2	0	0
2015-061	13	mouse 1	1.6	1.6	0	0
		mouse 2	1.2	1.2	0	0
		mouse 3	1.7	1.39	0.22	0
		mouse 4	1.8	1.8	0	0
		mouse 5	1.5	1.5	0	0
2015-008	20	mouse 1	1	0.09	0.77	0
		mouse 2	2	0.3	1.34	0
		mouse 3	0.5	0.21	0.21	0
		mouse 4	4	0.2	3.36	0
2015-060	21	mouse 1	2	0	0	2
		mouse 2	0	0	0	0
		mouse 3	0	0	0	0
		mouse 4	0.2	0	0	0.2
2014-013	12	mouse 1	0.7	0.04	0.02	0.62
		mouse 2	0.5	0	0	0.5
		mouse 3	2	1.7	0.2	0

UPN	Time from injection to analysis	MOUSE	hCD45%	CD45$^+$ CD33$^+$%	CD45$^+$ CD19$^+$%	CD45$^+$ CD3$^+$%
2015-018	12	mouse 1	2	0.16	0	0
		mouse 2	0	0	0	0
		mouse 3	2	0.16	0	0
2014-020	24	mouse 1	0.5	0	0.5	0
2015-059	29	mouse 1	71	1.42	ND *	8.52
		mouse 2	45	0.45	ND *	7.65
		mouse 3	8	0	ND *	7.2
2015-062	14	mouse 1	0.1	0.06	0.04	0
		mouse 2	0.2	0.17	0.03	0
		mouse 3	2.5	2.28	0.2	0
		mouse 4	0.3	0.12	0.18	0
		mouse 5	7	0.84	0.35	ND **
2015-021	15	mouse 1	11.33	0.09	11.24	0
		mouse 2	5.54	0.05	5.49	0
		mouse 3	12.66	0.1	12.56	0
		mouse 4	8.83	0.07	8.74	0
2015-055	16	mouse 1	3	0	0	3
		mouse 2	0.7	0	0.13	0.61
		mouse 3	0	0	0	0
		mouse 4	0.6	0.6	0	0
	35	mouse 5	0.4	0.4	0	0
		mouse 6	0.03	0.03	0	0
		mouse 7	0.08	0.08	0	0
		mouse 8	0.5	0.5	0	0
2015-057	10	mouse 1	64	64	0	0
		mouse 2	22	22	0	0
		mouse 3	40	40	0	0
		mouse 4	84	84	0	0
		mouse 5	56	56	0	0
2015-056	8	mouse 1	58	58	0	0
		mouse 2	30	30	0	0
		mouse 3	29	29	0	0
		mouse 4	47	47	0	0
2014-007	24	mouse 1	72	72	0	0
		mouse 2	71	71	0	0
		mouse 3	67	67	0	0
2014-042	11	mouse 1	80	80	0	0
		mouse 2	84	84	0	0
		mouse 3	67	67	0	0
2014-038	11	mouse 1	85	85	0	0
		mouse 2	87	87	0	0
2014-019	11	mouse 1	45	8.1	33.75	0
		mouse 2	70	10.5	11.9	47.6
2013-004	20	mouse 1	8	5.84	1.68	0
		mouse 2	2.5	1.43	0.75	0
		mouse 3	4.5	0.63	3.15	0
2015-049	28	mouse 1	50	50	0	0
		mouse 2	45	45	0	0
		mouse 3	57	57	0	0
		mouse 4	56	56	0	0
2014-037	19	mouse 1	88	88	0	0
		mouse 2	11	11	0	0
2015-052	43	mouse 1	28	25.48	0.84	2.52
		mouse 2	23	19.55	0.46	2.53
		mouse 3	52	49.4	2.6	2.6
2014-041	18	mouse 1	39	32.37	2.535	0
		mouse 2	39	30.42	5.46	0
		mouse 3	41	34.85	2.67	0
2014-039	22	mouse 1	48	48	0	0
		mouse 2	34	34	0	0
		mouse 3	40	40	0	0
2013-003	22	mouse 1	17	17	0	0
		mouse 2	85	85	0	0
		mouse 3	79	79	0	0
2014-043	12	mouse 1	80	80	0	0
		mouse 2	89	89	0	0
2015-001	12	mouse 1	93	93	0	0
		mouse 2	98	98	0	0
		mouse 3	95	95	0	0
		mouse 4	90	90	0	0

ND: no data

* CD19+ cells were detected in blood of these mice at week 12

** SSC/FSC/CD45 data indicating a lymphocyte morphology

Supplementary Table 10 | Order of lesion acquisition in 49 patients with lesions in epigenetic regulators in first position

UPN	Somatic events: event 1	event 2	event 3	event 4	event 5	event 6	event 7	event 8	event 9	event 10	event 11	Quantification of mutant alleles or cells*: evt. 1	evt. 2	evt. 3	evt. 4	evt. 5	evt. 6	evt. 7	evt. 8	evt. 9	evt. 10	evt. 11	
2014-002	t(8;21)	NRAS										0.864	0.41										
2014-007	inv16	KIT										0.903	0.435										
2014-038	MLL	FLT3-ITD										1.000	0.277										
2015-007	inv(16)	KIT<br>CSF3R<br>FLT3										0.667	0.176<br>0.097<br>0.052										*KIT*, *FLT3*, and *CSF3R* VAFs suggest the existence of 3 parallel sub-clones (65% of the cells). Alternative clonal architectures are possible.
2014-032	DNMT3A	RUNX1#1<br>RUNX1#2										0.338	0.226<br>0.071										An alternative linear clonal architecture with bi-allelic *RUNX1* mutation in a single clone is possible.
2014-025	IDH2	SRSF2										0.476	0.455										
2014-028	CUX1	DDX41	SRSF2									0.19	0.156	0.074									An alternative clonal architecture with parallel clones is possible.
2015-004	DNMT3A	IDH2	BCOR									0.466	0.464	0.409									*IDH2* and *BCOR* mutations lost at relapse.
2014-024	TET2#1	TET2#2	NPM1									0.426	0.388	0.373									Order of events confirmed by colony analysis.
2015-048	inv16	NRAS	SPI1									0.905	0.452	0.441									
2014-042	del(20q)	CCND3<br>WT1#1	WT1#2									0.569	0.017<br>0.176	0.021									*WT1*#1 lost at relapse. *CCND3* VAF=0.395 > *WT1*#2 VAF=0.327 at relapse, in a major clone, indicating linear acquisition.
2015-014	DNMT3A	SMC1A	NPM1									0.453	0.869	0.4									At relapse, loss of *SMC1A* mutation, and change in *NPM1* mutation (antedated by an *IDH2* mutation in the relapse clone).
2014-034	IDH2	BCOR#1	BCOR#2<br>CEBPA									0.403	0.37	0.147<br>0.124									
2015-024	TET2#1	TET2#2	NPM1									0.51	0.475	0.391									
2015-002	inv16	TET2	NRAS G12<br>NRAS Q61<br>Tri8									1.000	0.433	0.058<br>0.325<br>0.037									*NRAS* G12 and Q61 VAFs, and the percentage of cells with +8 suggest the existence of 3 parallel sub-clones representing 80% of the cells. A linear alternative clonal architecture is possible.
2014-015	del(20q)	U2AF1	RET									0.61	0.268	0.174									Order of events confirmed by colony analysis.
2014-020	DNMT3A	GATA2<br>FLT3-ITD	FLT3-TKD<br>FLT3-LOH									0.463	0.182<br>0.093	0.242<br>ND									Order of events confirmed by colony analysis. *FLT3*-LOH detected by colony analysis.
2014-022	TET2#1	BCLAF1	TET2#2<br>TET2#3	SRSF2<br>CTCF								0.426	0.199	0.278<br>0.065	0.305<br>0.063								Order of events confirmed by colony analysis. No colony with *CTCF* mutation. VAFs suggest that *CTCF* mutation occured in the *TET2*#3 mutant subclone.
2015-052	DNMT3A	IDH2	SRSF2	NPM1								0.483	0.469	0.44	0.308								
2014-036	DNMT3A	NPM1	FLT3	FLT3-LOH								0.447	0.5	0.5	0.5								*FLT3* LOH in 52% of the cells. *DNMT3A* mutant was placed before *NPM1* mutant based on the analysis of blood sample at relapse with 19/494 *DNMT3A* mutant reads and 0/167 *NPM1* mutant reads.
2015-062	DNMT3A	NPM1	add(X)	FLT3<br>PTPN11								0.409	0.346	0.61	0.188<br>0.157								An alternative clonal architecture with linear acquisition of *FLT3* and *PTPN11* mutations is possible.
2014-033	TET2	DNMT3A	RUNX1	FLT3-ITD#1<br>FLT3-ITD#2								0.519	0.431	0.352	0.13<br>0.13								An alternative clonal architecture with linear *FLT3*-ITD acquisition is possible.
2014-001	TET2	DNMT3A	NPM1<br>TET2	KRAS<br>NRAS								0.484	0.399	0.337<br>0.163	0.131<br>0.098								Order of events confirmed by colony analysis.
2014-019	MLL	SPI1	OXCT1	FLT3 M664I<br>FLT3 S451F								0.788	0.38	0.4	0.107<br>0.115								Order of events confirmed by colony analysis. No colony was found with *FLT3* S451F mutation. An alternative clonal architecture with linear acquisition of *FLT3* S451F mutation after M664I mutation is possible.
2013-001	MLL	HNRNPU	ABCC2	NRAS								0.995	0.22	0.36	0.39								Order of events confirmed by colony analysis.
2014-041	DNMT3A	IDH1	NPM1	PTPN11<br>NRAS								0.468	0.498	0.438	0.161<br>0.246								Order of events confirmed by colony analysis. No *NRAS* mutant colony was found. VAFs indicate that *NRAS* and *PTPN11* mutations occured in distinct subclones (NRAS > PTPN11, non overlapping 95% CI)
2015-008	inv16	tri22	KIT#1<br>KIT#2	ATM								0.955	0.955	0.325<br>0.052	0.233								An alternative linear clonal architecture is possible.
2015-021	TET2	-Y	TET2-LOH	NPM1								0.959	1.000	0.959	0.376								*TET2* LOH in 90% of the cells, indicating that 100% of the cells are *TET2* mutant
2014-021	TET2	t(8;21)	FLT3<br>-7	del11p								0.5	0.865	0.476<br>0.01	0.945								Karyotype, FISH, and NGS data at relapse indicate the loss of del(11p) and *FLT3* mutation.
2014-039	TET2	DNMT3A	TET2-LOH	NPM1<br>ZRSR2								0.916	0.483	0.916	0.318<br>0.037								Deep sequencing and follow-up analyses indicating the presence of the *ZRSR2* mutation in a subclone at diagnosis.
2015-018	IDH2	der(11)	DNMT3A	FBXW7								0.512	0.955	0.42	0.337								
2015-059	DNMT3A	FLT3-ITD	FLT3-LOH	NPM1								0.474	0.943	0.943	0.456								*FLT3* LOH in 94% of the cells.
2015-022	TET2	DNMT3A	TET2-LOH	NPM1<br>CEBPA								0.941	0.462	0.941	0.171<br>0.267								Order of events confirmed by colony analysis, with a complex pattern. *CEBPA* analysis was not done on colonies. VAFs suggest that *NPM1* and *CEBPA* mutations occured in distinct subclones.
2014-029	DNMT3A	ASXL1	SRSF2	IDH1<br>IDH2	RUNX1							0.35	0.33	0.32	0.45<br>0.007	0.014							Changes at relapse indicate that *DNMT3A*, *ASXL1*, and *SRSF2* mutations preceded *IDH1* mutation. VAFs suggest that *IDH1* and *IDH2* mutations occured in distinct clones, and that *RUNX1* mutation is in last position.
2015-006	IDH2	NOTCH1	SRSF2	NPM1	+Y							0.512	0.474	0.443	0.402	0.2							
2014-017	IDH1	DNMT3A	ATM	NPM1	KDM6A							0.482	0.462	0.456	0.482	0.409							Loss of *NPM1* and *KDM6A* mutations at relapse.
2015-019	BCOR	IDH2	DNMT3A	RUNX1<br>STAG2	FLT3							0.491	0.453	0.446	0.294<br>0.071	0.273							An alternative linear clonal architecture is possible.
2013-002	ASXL1	EZH2#1	EZH2#2	RUNX1	FLT3							0.357	0.322	0.291	0.316	0.088							Order of events confirmed by colony analysis.
2014-003	MLL	JAG1	RALBP1	CDC27	FLT3							0.975	0.38	0.38	0.42	0.157							Order of events confirmed by colony analysis.
2015-049	ASXL1	SRSF2	BCOR	ETV6	RUNX1							0.511	0.481	0.919	0.459	0.364							
2013-004	MLL	XRCC5	ASXL1	KRAS	SOS2							0.864	0.43	0.43	0.511	0.49							Order of events confirmed by colony analysis.
2014-016	TET2	DNMT3A	JAK1	NPM1	FLT3-ITD	FLT3-LOH						0.454	0.458	0.43	0.413	0.709	0.709						Order of events confirmed by colony analysis. *FLT3*-LOH in 83% of the cells, detected in colonies.
2013-003	TET2	SMC1A	DNMT3A	NPM1	FLT3-ITD	CBL						0.483	0.478	0.439	0.413	0.412	0.07						Alternative clonal architectures are possible with *CBL* mutation branching after either *SMC1A*, *DNMT3A*, or *NPM1* mutations
2014-043	DNMT3A	CTCF	TET2#1	TET2#2	NPM1	TET3						0.547	0.532	0.48	0.421	0.291	0.275						Changes at relapse indicating that the *TET3* mutation occured after the *NPM1* mutation .
2015-005	DNMT3A	ASXL1	IDH1	RUNX1	DSCAM	LOH chr21						0.359	0.303	0.316	0.607	0.565	0.624						Chr. 21 LOH in 62% of the cells. *IDH1*, *RUNX1*, and *DSCAM* mutations lost at relapse.
2014-037	TET2	EZH2	BCOR	ASXL1	CEBPA<br>RUNX1	PTPN11<br>BCORL1<br>WT1	FLT3-ITD					0.402	0.4 (10 reads)	0.447	0.424	0.35<br>0.106	0.137<br>0.25<br>0.016	0.207					Loss of *PTPN11* mutation at relapse. VAFs, complete remission, and relapse NGS analyses suggest the proposed clonal architecture. Alternative clonal architectures are possible.
2015-054	KDM6A	CTCF	TET2#1	t(12;13)	TET2#2	PHF6	t(7;12)	ASXL1				0.529	0.506	0.501	1.000	0.5	0.494	0.857	0.356				
2015-001	TET3	TET2#1	RUNX1	CEBPA	TET2#2	ASXL1	EZH2	NRAS	CEBPA			0.535	0.511	0.5	0.465	0.464	0.451	0.451	0.379	0.297			
2015-055	TET2	EZH2	t(1;16)	SRSF2	tri16	tri19	tri8	ASXL1	IDH2	tri13	NF1	0.601	0.517	1.000	0.495	0.923	0.541	0.436	0.215	0.191	0.274	0.086	

Epigenetic events	Proliferation/signaling mut
NPM1/Factors	Other events

Subclonal events with possible alternative architectures:

Delineates lesions with order of occurrence inferred from colony analysis and/or allele/cell quantification (non overlapping 95% confidence intervals), and/or remission/relapse data → lesion1 | lesion2

*Black: VAFs. Blue: VAFs (X linked gene, male patient). Red: quantification of chromosome aberrations (karyotype, interphasic FISH, SNP analysis of NGS data) Green: FLT3-ITD quantification (sizing).

Supplementary Table 11 | Order of lesion acquisition in 25 patients with no evidence for lesions in epigenetic regulators in first position

UPN	Somatic events<br>event 1	event 2	event 3	event 4	event 5	event 6	event 7	event 8	event 9	event 10	11 to 15
2014-004	no somatic mutation detected										
2014-010	*RUNX1*										
2015-053	*FLT3-ITD*	*CEBPA*									
2014-006	*NPM1*	*DSCAM*	*IDH1*								
2013-006	*CCND3*	*WT1*	*FLT3-ITD#1*<br>*FLT3-ITD#2*								
2015-051	*GATA2*	*CEBPA*	*NRAS Q61*<br>*NRAS G12*<br>*CCND3*	 <br>*KIT*							
2014-008	*U2AF1*	del(16q)	*FLT3-ITD*	*FLT3-LOH*							
2014-009	*SF3B1*	*PTPN11*	-7	*FLT3*							
2015-061	*SMC1A*	*NRAS*	*IDH1*	*NPM1*							
2015-056	*DDX1*	del(5q)	*RUNX1*	-7	*KRAS*						
2014-040	t(3;13)	t(5;8)	del(7q)	*BCOR*	*NRAS*						
2014-018	*BRCC3*	*CDK19*	*SRSF2*	*DDX41*	*OGT*	*CUX1*					
2015-060	*PHF6*	*RUNX1*	*IDH1*	*BCOR*	*RUNX1*	*ASXL1*					
2014-031	*RUNX1*	tri.21	tri.10	*BCOR*	i(18q)	*ETV6*					
2014-013	*CEBPA*	tri. 8	*NPM1*	*FLT3-ITD*	*WT1**	*PDS5B*	*WT1*				
2015-003	*NRAS*	del(6q)	t(?x;8)	*SH2B2*	*TP53*	*SF3B1*	*add(6)*				
2014-027	*TP53*	-16	del(5q)	*DNMT3A*	t(3;7)	*IDH1*	*TP53*				
2015-057	*JAK2*	*JAK2*^{LOH}	*TP53*	del(5q)	-7	der(17)	add(22)x2	del(1p)			
2015-017	*NUMA1*	del(7q?)	del(9q)	*IDH1*	*U2AF1*	*RB1*	tri.11	*JAK2*			
2014-014	*TP53*	*TP53*	t(8;5)	del(7q)	add(3p)	del(12p)	?add(14q	der(15)			
2015-027	*NF1*	*TP53*	*DNMT3A*	*TP53*	t(5;12)	-16	-18	add(17p)	t(15;20)	der(11)	
2015-047	*TP53*	-18	-7	del(5q)	mar2(17)	add(11p)	add(5q)	-8	?20	add(3q)	
2015-050	*TP53*	*NSD1*	*DNMT3A*	Complex karyotype with 5q, 11p, 17p rearrangements, trisomies (2,6,8,10.11,13,19,21							
2015-058	*SMC1A*	*RUNX1*	*SF3B1*	*FLT3-ITD*	relapse sample						
2015-020	*STAG2*	*BCOR*	*SRSF2*	*FLT3-ITD*	*FLT3-LOH*	*ASXL1*	Sample collected at time of re-evolution after treatment failure at relapse.				

UPN	Quantification of mutant alleles or cells*<br>evt. 1	evt. 2	evt. 3	evt. 4	evt. 5	evt. 6	evt. 7	evt. 8	evt. 9	evt. 10	evt.s 11 to 15	
2014-004	no somatic mutation detected											
2014-010	0.49											
2015-053	0.324	0.208										
2014-006	0.377	0.369	0.352									
2013-006	0.467	0.378	0.167<br>0.275									An alternative clonal architecture with linear FLT3-ITD acquisition is possible.
2015-051	0.474	0.44	0.19<br>0.102<br>0.151	 <br>0.061								An alternative clonal architecture with linear acquisition or different branching events is possible.
2014-008	0.464	0.955	0.17	0.17								Order of events confirmed by colony analysis.FLT3 LOH in 23% of the cells, also detected on colonies
2014-009	0.403	0.446	0.325	0.12								Order of events confirmed by colony analysis.
2015-061	0.857	0.378	0.371	0.291								
2015-056	0.492	0.888	0.437	0.869	0.413							
2014-040	1.000	1.000	1.000	0.951	0.474							
2014-018	0.317	0.270	0.240	0.218	0.100	0.080						
2015-060	0.467	0.224	0.208	0.36	0.174	0.163						
2014-031	0.319	0.638	0.502	0.266	0.192	0.094						84% of *RUNX1* mutant cells (+21). An alternative clonal architecture is possible for *BCOR,* i(18q), and *ETV6* lesions.
2014-013	0.525	1,000	0.466	0.248	0.195	0.164	0.111					An alternative clonal architecture is possible for FLT3, WT1, and PDS5B mutations.
2015-003	0.377	0.263	0.263	0.129	0.11	0.09	0.105					Other karyotypic aberrations in 3/19 metaphases
2014-027	0.486	0.957	0.947	0.44	0.958	0.44	0.411					Colony analysis of TP53, DNMT3A, IDH1, and chromosome 7 lesions,<br>VAFs and cytogenetic/FISH data, and follow up data suggest the proposed clonal architecture.
2015-057	0.999	0.999	0.401	0.53	0.51	0.46	<0.46	<0.46				Add(22) found in all metaphases with -7, del(1p) found subclonal with other various rearrangements.
2015-017	0.374	0.6	0.6	0.279	0.239	0.196	0.3	0.106				An alternative clonal architecture is possible for RB1, JAK2, and chromosome 11 lesions.
2014-014	0.335	0.348	NA	0.647	0.583	NA	NA	NA				Cytogenetic follow up analysis found 5q aberration as first chromosomal aberration,<br>SNP analysis of NGS data indicate 64,7% of cells with del(7q), and 58,3% with PBRM1 deletion (3p11)
2015-027	0.487	0.47	0.445	0.43	0.903	0.896	0.895	NA	NA	0.793		Colony analysis indicate that *TP53* and *DNMT3A* mutations precede chromosomal aberrations.<br>SNP analysis of NGS data was used to quantify chromosome 5, 16, and 18 aberrations. Der(11) in 317/400 nuclei (FISH)
2015-047	0.433	0.782	0.752	0.752	0.752	0.641	NA					Complex karyotype, FISH analysis showing 108/176 nuclei with del(5q) and del (7q).<br>VAF analysis of SNPs on chr.7, 18, 6, 11, and 3 showing LOHs, 50% TP53 mutant at relapse.
2015-050	0.89	0.635	0.617	NA								
2015-058	0.973	0.482	0.48	0.324								
2015-020	0.982	0.969	0.429	0.456	0.211							Karyotype not done. FLT3-LOH detected in 53,2% of the cells.

Epigenetic events | Proliferation/signaling mut
NPM1/Factors | Other events

Subclonal events with possible alternative architectures:

Delineates lesions with order of occurrence inferred from colony analysis and/or allele/cell quantification (non overlaping 95% confidence intervals), and/or remission/relapse data → lesion1 | lesion2

*Black: VAFs. Blue: VAFs (X linked gene, male patient). Red: quantification of chromosome aberrations (karyotype, interphasic FISH, SNP analysis of NGS data) Green: FLT3-ITD quantification (sizing).

Supplementary Table 12 | Primers used for Sanger sequencing and FLT3-ITD detection (5'-3')

ABCC2_V669A_F	TCAATACCCAACCCCTGCTA
ABCC2_V669A_R	ATTCGGGAGTCAGAGGCTTT
AKAP13_G1613R_FWD	CCAAAGGGATTTTGTGTCCT
AKAP13_G1613R_REV	CACCACTGAATGGGTGTCTG
APH1A_P446A_FWD	GGTGGGTTTGAATCTGCACT
APH1A_P446A_REV	ATACAGGGCGAGGACATCAG
ASXL1_delGT_F	AGAGGTCACCACTGCCATAGA
ASXL1_delGT_R	TGGGTATGCTCCCCATTTAG
ASXL1_FWD	ACCCTGGGTGGTTAAAGGTC
ASXL1_FWD	ACCCTGGGTGGTTAAAGGTC
ASXL1_FWD	ACCCTGGGTGGTTAAAGGTC
ASXL1_G1397S_FWD	GGGGTCCTCTTAAGGCAAAT
ASXL1_G1397S_REV	GAGTTGGGAGGGGAGAGAAG
ASXL1_Q373*_FWD	CCTGAAACTGATGGCTGTGA
ASXL1_Q373*_R	CAGATTCCTTCTGGCTCTGG
ASXL1_Q882STOP_FWD	AATCCTCACCGACTGATTGC
ASXL1_Q882STOP_REV	CATTCGATGGGATGGGTATC
ASXL1_REV	CTCTGCCACCTCCCTCATC
ASXL1_REV	CTCTGCCACCTCCCTCATC
ASXL1_REV	CTCTGCCACCTCCCTCATC
ASXL1_Y300C_FWD	GATGAGGGAGGTGGCAGAG
ASXL1_Y300C_R	GAAGGCAGGTCCTCTCTCCT
ATM_D1853V_FWD	ATATGTCAACGGGGCATGAA
ATM_D1853V_R	GTGTCGAAGACAGCTGGTGA
ATM_F858L_FWD	GTGCCCAGCCTGATTAGGTA
ATM_F858L_FWD	GTGCCCAGCCTGATTAGGTA
ATM_F858L_REV	CTTTGGCTCTCTCCAGGTTC
ATM_F858L_REV	CTTTGGCTCTCTCCAGGTTC
ATM_L1046F_FWD	GAAAACTTACTTGATTTCAGGCATC
ATM_L1046F_REV	TGGCCCATTTTGAATAAGGA
ATM_P1054R_FWD	TGCTTGAGGTGAGTTTTTGC
ATM_P1054R_REV	GATTGACTCTGCAGCCAACA
ATM_P2029R_FWD	TTGTTGTTTCCATGTTTTCAGG
ATM_P2029R_R	CCACATTGCTTCGTGTTCAT
ATM_Q1128R_FWD	ATTGTTCCAGGACACGAAGG
ATM_Q1128R_FWD	ATTGTTCCAGGACACGAAGG
ATM_Q1128R_REV	ATGGGATATTCATAGCAAGCA
ATM_Q1128R_REV	ATGGGATATTCATAGCAAGCA
ATP2C2_M466I_F	CAACAATGCGGTCATCAGAA
ATP2C2_M466I_R	CTCCTCCACCACCAACAGAT
BCLAF1_K162E_FWD	CGGATGTGGCTGATGACTTA
BCLAF1_K162E_REV	CAGATCTCGCCGGTCATATAG
BCLAF1_R107C_FWD	ATGGGTACAGAGGAAGGGGTA
BCLAF1_R107C_REV	TGGGGATGAAGAACGAGAAG
BCLAF1_S343P_FWD	GGGATTGGGCAACACATTTA
BCLAF1_S343P_REV	CCCTCCCTTTCTCTGATCCT
BCOR_E1167*_FWD	CAACCTGAAGGTGTGCATTG
BCOR_E1167*_REV	TCTTCCGACCAGCTTCTGTT
BCOR_FER_FWD	AAAACAAGGCATTGGACTGG
BCOR_FER_REV	TACTGTGCTTGGCAGGAGTG
BCOR_LET_FWD	ACCGAGTTTCAAAGCAAACG
BCOR_LET_REV	CCAGGAAACAGACTGCCATT
BCOR_R810STOP_FWD	CCCACCGACAAGAACCTAAA
BCOR_R810STOP_REV	TCTCTCAGGGCGATGAAATC
BCORL1_R1196STOP_FWD	CCAGGAATCCACCAAGAAAA
BCORL1_R1196STOP_REV	TTCCAGAGTGAAGGAGTCAGC
BRPF1_G1058S_FWD	ACTTTCCCAGAGGACAGCAG
BRPF1_G1058S_REV	GGCCCAGACTCTTCCTTACC
CBL_L380P_FWD	GGAAACAAGTCTTCACTTTTTCTGT
CBL_L380P_REV	CCGTACCTGCCAGGATGTAA
CBL_R844W_FWD	TGCAGGTGAAGATGCTTTTG
CBL_R844W_REV	TTGGCTTTTCCCTCCTACCT
CCND3_D205H_FWD	GAAGCTGCACTCAGGGAGAG
CCND3_D205H_REV	CCCTTCAGGCTTAGATGTGG
CCND3_FS_FWD	GAAGCTGCACTCAGGGAGAG
CCND3_FS_REV	CCCTTCAGGCTTAGATGTGG
CHEK2_G222STOP_FWD	CCACTGAGAATGCCACTTGA
CHEK2_G222STOP_REV	AGAAACTCCCACCACAGCAC
CSF3R_W547STOP_FWD	GGAAGCCACAAGAAGTCCAA

CSF3R_W547STOP_REV	TGTGGGGGAACTGAGGATAG
CTCF_R377H_FWD	ACTTCAGTGCCCCAAAGCTA
CTCF_R377H_REV	TCATGTGCCTTTTCAGCTTG
CUX1_FWD	AGAAAGGCCGAGAACCCTTC
CUX1_REV	AGTTCCCTCCTCCCTCTCTG
CUX2_P834L_FWD	TCCTCCTCTGGCTACTCTGG
CUX2_P834L_R	ACGTAGGCCGGGTAGTAGG
DAAM2_H650Q_FWD	GGCACCGTATGGAATGAGAT
DAAM2_H650Q_REV	GAAGGGGAAGATGGAGGAAG
DAAM2_R335Q_FWD	CTCAACCAAGGGTGTGTCCT
DAAM2_R335Q_REV	CACGGGGTCTAGAAAAGTGG
DAAM2_R481Q_FWD	TCCAGAACACATGGAGCTTG
DAAM2_R481Q_REV	TGTAAAGGGGGAGATGCTTG
DAXX_D339H_FWD	AAAAGTGAGGAGGGCGAGAG
DAXX_D339H_R	CAGAGGGTTTGGTTCTTGCT
DDX41_G267E_FWD	GGTTCAGGCAAGACACTGGT
DDX41_G267E_REV	CGGCAGTAGTACTCCAGGATG
DDX41_NIC_FWD	ATGGGTTAGGCCGGAAAAG
DDX41_NIC_REV	GCTCTGCAGTCACCTCCAAT
DDX41_R525H_FW	TGACATGCCAGAGGAGATTG
DDX41_R525H_R	ACCCAGGGAACAGCTAAGGT
DDX41_T472S_FWD	TGCTGCAGTAGCAGTGCTTT
DDX41_T472S_REV	AATTGATGACGTGCTGGATG
DIS3_D408N_FWD	CCTCTTGGTTTCAGGGACAC
DIS3_D408N_FWD	TAATGCCTCTTGGTTTCAGG
DIS3_D408N_R	TAATGCTCCAGGGCATCTTT
DIS3_D408N_REV	TAATGCTCCAGGGCATCTTT
DNMT3A_C162Y_FWD	CTCCTCTTTGCATCGGGTAA
DNMT3A_C162Y_REV	ACCTGCACTCCAACTTCCAG
DNMT3A_D513E_FWD	CTTCCTGTCTGCCTCTGTCC
DNMT3A_D513E_REV	CAAGGAGGAAGCCTATGTGC
DNMT3A_ex23_F	CTGCCCTCTCTGCCTTTTCT
DNMT3A_ex23_R	TTGTGTCGCTACCTCAGTTTG
dnmt3a_fwd	GCACCCTGGACTCTTTTCTG
DNMT3A_G34S_FWD	TACACTGCTGGGATCCACCT
DNMT3A_G34S_REV	TCCCTGCAGGACATACATCA
DNMT3A_L548H_FWD	GGCTTTCTCTTCCGACCTCT
DNMT3A_L548H_R	ATGAAGCAGCAGTCCAAGGT
dnmt3a_rev	TACCTTGCAGTTTTGGCACA
DNMT3A_V527L_FWD	CTTCCTGTCTGCCTCTGTCC
DNMT3A_V527L_REV	CAAGGAGGAAGCCTATGTGC
DNMT3A_W220STOP_FWD	CTCAGAGTCTGGCCTTGAGC
DNMT3A_W220STOP_REV	TGCCCTCATTTACCTTCTGG
dnmt3afs_fwd	AGGTGGCCTTGCTAATTCCT
dnmt3afs_rev	CTTTGGTGGCATTCTTGTCC
DOK2_FWD	CTGCCCTGTCCCTCTATGAC
DOK2_REV	GGAGGAGTCACCAGCAGAAG
DSCAM_E1836STOP_FWD	GATGGTGAGTCGTCCGTCTT
DSCAM_E1836STOP_REV	GGAGGTCAGACTGTCCGTGT
DSCAM_K322R_FWD	CGTGATGCACTTGCTTTGTT
DSCAM_K322R_REV	TCCAGGGTTGAGGATTTCAC
DSCAM_S171P_FWD	GTGGTTGGGTTGCTTTCATT
DSCAM_S171P_REV	ACAAAAAGTCTGGCGCTGTT
EGR1_P459S_FWD	AAAGTGTTGTGGCCTCTTCG
EGR1_P459S_REV	GGGGAACAGAGGAGTACGTG
EZH2_G538STOP_FWD	AAGCACAGTGCAACACCAAG
EZH2_G538STOP_REV	TTTGCCCCAGCTAAATCATC
EZH2_K629M_FWD	AGGCAAACCCTGAAGAACTG
EZH2_K629M_REV	GGACTGAAAAGGGAGTTCCA
EZH2_N637K_FWD	AGGCAAACCCTGAAGAACTG
EZH2_N637K_REV	GGACTGAAAAGGGAGTTCCA
EZH2_R249*_FWD	CCTGAATGTACCCCCAACAT
EZH2_R249*_R	AGCACTCTCCAAGCTGCTTTA
FANCA_G501S_F	CTGGGACAGGTGTGAGGAGT
FANCA_G501S_R	CCAGGCAGTTCCCAGACTAA
FANCA_G809D_F	TCTTCAGTGCCCTGGAAAAT
FANCA_G809D_R	AACAAGGAATCCCTCGTCCT
FANCA_L63V_F	ACCACGCCCAACCCTTTT
FANCA_L63V_R	TGCCTCCTGAACTCCAGAAT
FANCA_R73S_F	AGGCCATGTCCGACTCGT
FANCA_R73S_R	GGCGTTCGGGAAAGAAGG
FANCA_T266A_F	TCCTGAAGTGCCGTTTCTTT
FANCA_T266A_R	TTACAGTCTGGGCTGCAGTG
FBXW7_E112A_FWD	TTGGACCATGGTTCTGAGGT

FBXW7_E112A_REV	TTTCCGGTAATCTCAAAATGTG
FBXW7_F86L_FWD	AGCTGGCTTTTGGAAATGAA
FBXW7_F86L_REV	CCTCATCTTGTTCACCAGCA
FBXW7_FWD	ATTTTCCCCTGCAGAATGTG
FBXW7_REV	TGAGTTGCTGTTGCTGTTCC
FLT3_A680V_FWD	GCTCTGAAAGAGAGGCACTCA
FLT3_A680V_R	CCACTTGGGTTTGAGAGTTCA
FLT3_E444Q_FWD	CCACCTTGGCTTCACAAAGT
FLT3_E444Q_REV	TTCTTCCAGGTCCAAGATGG
FLT3_M664I_FWD	AATGCAGATTGACTCTGAGCTG
FLT3_M664I_R	GGTGATTTTCGTGGAAGTGG
FLT3_Q580P_FWD	TCTGCAGAACTGCCTATTCCT
FLT3_Q580P_REV	CTGCAGAAACATTTGGCACA
FLT3_S451F_FWD	GCCCAGCCAGTGAGCTTAT
FLT3_S451F_R	GGTCAGAGAGTTTTATGTTCTTCCA
FLT3-ITD_F	FAM-TGGTGTTTGTCTCTTCTTCATTGT
FLT3-ITD_R	GTTGCGTTCATCACTTTTCCAA
FOXP1_T2901S_FWD	CCACGCATCCTCTGTGTTAC
FOXP1_T2901S_REV	GTTGGGGTCGTTGGAGTATG
FZD1_P48S_FWD	GCGAGCTGGGAACTTTGTG
FZD1_P48S_REV	GCTCTGTTGCTGCTGAGG
GATA2_N317S_F	CCGGGAGTGTGTCAACTGT
GATA2_N317S_R	AAAGCGTCTGCATTTGAAGG
GATAD2B_R10C_FWD	AACTTCACCATTCCACCTTTTT
GATAD2B_R10C_REV	TTGCCAAATCCTTCCTTTTG
GCSH_S9L_F	CTCTGCCCAGTCAGGCTCT
GCSH_S9L_R	CCAGGGATCCCAACAGAAAT
HJURP_T539I_FWD	CGCAGGCTGAGTTTACCTTC
HJURP_T539I_REV	GTTTTGCTGGGTGACACTGA
HNRNPU_INDEL_F	TCCAGCGAAAAGCTGTTGTA
HNRNPU_INDEL_R	TTTCAGGAGGCCTAAGTCCTAA
HPR_H272D_F	CTGGAGGAGGACACCTGGTA
HPR_H272D_R	ACCCATCAGCTTCAAACCAC
IDH1_F	CCATTTGTCTGAAAAACTTTGCT
IDH1_R	GCAAAATCACATTATTGCCAAC
IDH2_F	GCTGCAGTGGGACCACTATT
IDH2_R	GTGCCCAGGTCAGTGGAT
IDH2_R261H_FWD	ATGCCATCCAGAAGAAATGG
IDH2_R261H_REV	AGCTGAGCCAAATGCACTCT
IKZF1_G103A_FWD	ATTCACCCAGAAGGGCAAC
IKZF1_G103A_REV	GACTCAGGGTTAGCCAGCAA
JAG1_T713M_F	CCACTGGGACTCACACTGAA
JAG1_T713M_R	CCAGGCCCAGAGAAATATCA
JAK1_L4V_FWD	CCAACACCAAATGTTTCTGC
JAK1_L4V_REV	CCTCCAGGTTCACCTCAGTC
JAK3_V722I_FWD	TCCCACTTTCATTCCCTCAG
JAK3_V722I_REV	TTCTGTCAAAGTGGGGGTTC
KDM6A_FS_FWD	CATTTGGCCTCCTCTAACCA
KDM6A_FS_REV	CCTGTAATTTCTGTGGTGTCAAA
KIT_D812Y_FWD	TGGTTTTCTTTTCTCCTCCAA
KIT_D812Y_REV	TGCAGGACTGTCAAGCAGAG
KIT_OLI_FWD	GCTGAGGTTTTCCAGCACTC
KIT_OLI_REV	CCTCTGCTCAGTTCCTGGAC
KIT_V526I_FWD	TGCCAAAGTTTGTGATTCCA
KIT_V526I_REV	GTGGGGAGAAAGGGAAAAAT
KRAS_G13D_FWD	CTTAAGCGTCGATGGAGGAG
KRAS_G13D_REV	AGAATGGTCCTGCACCAGTAA
KRAS_Q61H_FWD	TGTGTTTCTCCCTTCTCAGGA
KRAS_Q61H_R	GCATGGCATTAGCAAAGACTC
MAML1_M759R_FWD	CCCTGGACATGCTTCAGTTT
MAML1_M759R_REV	CCCAGAGAGTTCTGCCCATA
MAP2K2_P16L_FWD	GGCTCCCGATCCCGTTAT
MAP2K2_P16L_R	ACCCCTGCCCACTCACTC
MLL2_H1525R_FWD	CTTCCACTGTGAATGGCAGA
MLL2_H1525R_REV	TGGAGAACAGAGACTGGAGGA
MLL2_P1157T_FWD	CTCTGGATGGGATTGATGCT
MLL2_P1157T_REV	CGTGGCTCTTCCTGTTCTTC
MLL2_P2557L_FWD	TTCACTTTCCCTCAGGCAGT
MLL2_P2557L_REV	GTGGAAGTTCCCTGTGGCTA
MLL2_R1189C_FWD	CTCTGGATGGGATTGATGCT
MLL2_R1189C_REV	CTCTGAGCCAGGAAAACTGG
MLL2_R1299C_FWD	TGACTCACTATTGTGCGATGC
MLL2_R1299C_REV	CACAAAGCAAGGTGGGAAAG
MLL3_E3662G_FWD	TATAACTGCCCCACCGACTC

MLL3_E3662G_REV	GGTTTCCATGGAGAGCTTGT
MPL_T374A_FWD	CTGCCACTTCAAGTCACGAA
MPL_T374A_REV	GGCACAGGGTCAGATTCAGT
MPL_T481S_FWD	GCGATCTCGCTACCGTTTAC
MPL_T481S_R	CACCAAGGAGATCCAGGCTA
MSR1_D174Y_FWD	GGAACACATGAGCAACATGG
MSR1_D174Y_REV	GTTGAGCTGCAAATCAAGCA
MSR1_R293STOP_FWD	CCCATTTTTCCTATGCTTTACAA
MSR1_R293STOP_REV	AGCAATCCTCCCCTACACAT
NCOA7_D219E_FWD	AGATGGGGGCAAGAGAGAAT
NCOA7_D219E_REV	TGCCCTCTACATGGACCTTC
NCSTN_A298S_FWD	CTCCTGGGTTGTCTCCATTG
NCSTN_A298S_REV	GGTCACATCAGGTGCCTTTT
NF1_A2315T_FWD	TCTGCACAAAGCCCTCTTTT
NF1_A2315T_REV	AGGAACCTCAAGGCAAAGTT
NF1_FWD	TGAGTTTTAGAGGCTGTTAATTTGC
NF1_REV	TTGCAGCACTTTCTGTCAGC
NOTCH1_S1588G_FWD	ACTGCAAGGACCACTTCAGC
NOTCH1_S1588G_R	CACGCTTGAAGACCACGTT
NOTCH1_V1671I_FWD	GCAACAGCTCCTTCCACTTC
NOTCH1_V1671I_REV	CGGAACCTCCGTCTCTTTTA
NOTCH1_V1676I_FWD	GTCCTGACTGTGGCGTCAT
NOTCH1_V1676I_REV	CCTCGATCTTGTAGGGGATG
NOTCH1_V2536I_FWD	ACAGCTACTCCTCGCCTGTG
NOTCH1_V2536I_REV	TACTTGAAGGCCTCCGGAAT
NPM1-F	GTGTTGTGGTTCCTTAACCACAT
NPM1-R (TYPE A)	TCCTCCACTGCCAGACAGAG
NPM1-R (TYPE D)	CTCCACTGCCAGGCAGAG
NRAS_G12S_FWD	GATGTGGCTCGCCAATTAAC
NRAS_G12S_REV	CACTGGGCCTCACCTCTATG
NRAS_G13D_FWD	GGTTTCCAACAGGTTCTTGC
NRAS_G13D_REV	CACTGGGCCTCACCTCTATG
NRAS_Q61H_F	CACCCCCAGGATTCTTACAG
NRAS_Q61H_R	CCCCATAAAGATTCAGAACACA
NSD1_F1203C_FWD	GCCCAGAGCTGGACTCTGTA
NSD1_F1203C_REV	GAATTCCTGGCTCCTTTTCC
NUMA1_N314D_FWD	TGCGTGACAAGAATGAGAGG
NUMA1_N314D_REV	CACCACCTTAACACCCACCT
OXCT1_R285Q_F	TGGGAGAGAGCTCATCTTTTT
OXCT1_R285Q_R	CATGCCATCCTCAAACTCAA
PRIMPOL_INDEL_F	CATCACCTAGCAGGCTGTCA
PRIMPOL_INDEL_R	AAAAACAAAAATTGGAAAGCAAA
PTPN11_A72V_FWD	ATGGAGCTGTCACCCACATC
PTPN11_A72V_REV	TCTGACACTCAGGGCACAAG
PTPN11_F285S_FWD	TGGACTAGGCTGGGGAGTAA
PTPN11_F285S_REV	TTTCAGGACATGAGGAAGGA
PTPN11_Q510H_FWD	CCTGGCTCTGCAGTTTCTCT
PTPN11_Q510H_REV	CCTGTCCTCCTGCTCAAAAG
RALBP1_INDEL_F	TGCGAGACCTTCCAGAGAAT
RALBP1_INDEL_R	CCATGTACCTGCACAGTTGG
RB1_A15T_FWD	GGTTTTTCTCAGGGGACGTT
RB1_A15T_REV	CTGTCCTGCTCTGGGTCCT
RET_G733D_F	GGAGGATCCAAAGTGGGAAT
RET_G733D_R	AGCCGCTCTAGAACAGCATT
RUNX1_ex1_FWD	GCTGAAACAGTGACCTGTCTTGG
RUNX1_ex1_REV	AGCTGCCATTTCATTACAGGCAAAGC
RUNX1_ex2_FWD	CTCCAGTGCTAAAAAGTGTAAG
RUNX1_ex2_REV	CATATACACATCTATGAAGGTGTGTAC
RUNX1_ex3_FWD	AGCTGCTTGCTGAAGATCCG
RUNX1_ex3_REV	GCCTGTCCTCCCACCACCCTCTC
RUNX1_ex4_FWD	CAACCTAAAAAGAAATCATTGAA
RUNX1_ex4_REV	CCGAGTTTCTAGGGATTCCA
RUNX1_ex5_FWD	GAAGGGCTGGACAGCATAAA
RUNX1_ex5_REV	GAAAGGTTGAACCCAAGGAA
RUNX1_ex6_fwd	CCCCAGTTTTAGGAAATCCA
RUNX1_ex6_FWD	CCCCAGTTTTAGGAAATCCA
RUNX1_ex6_rev	AGTTGGTCTGGGAAGGTGTG
RUNX1_ex6_REV	AGTTGGTCTGGGAAGGTGTG
RUNX1_ex7_FWD	AACAGAGGCAGATACTTGGACT
RUNX1_ex7_REV	CTTCATGCACCTCTAGTCTCCTG
RUNX1_ex8_FWD	CCATCCTCCTAGGCGGTAT
RUNX1_ex8_REV	GGCTGGTCGCGAACAGGAG
RUNX1_R177Q_FWD	GCCACCAACCTCATTCTGTT
RUNX1_R177Q_R	GGTTGAACCCAAGGAATCTG

SETBP1_C1287I_FWD	CTTTGAGGTGGACACCCTGT
SETBP1_C1287I_REV	CGTTCCAAAGCCTTCATAGC
SETBP1_G870S_FWD	GAAGCTGTCTCCACCCAGAC
SETBP1_G870S_REV	CCAGGGAGCAGAAATCAAAA
SF3A1_N78T_FWD	GTTCCAATGGGCAGTGTTCT
SF3A1_N78T_REV	TTCCTTGAACTCGCTGACCT
SF3A1_P589A_FWD	TCTCATCAGCCCTGAGTCCT
SF3A1_P589A_REV	GAACTCCTCCTCTGGCATGA
SH2B2_A392G_FWD	TTTGTCCCCCTTTCTCCTTC
SH2B2_A392G_REV	GTCTCACTTTGGCGGATCAC
SH2B3_L295V_FWD	TACAGCAGACCCAACCCTGT
SH2B3_L295V_R	TGCATCTCTGCTTCTGTGCT
SHKBP1_G344S_FWD	ACGGAAGGAAAGGAATGGAA
SHKBP1_G344S_REV	GAGCGGGTTTGTTCCAAGT
SMC1A_R699C_FWD	GTCAGCCACCACCTTACCAC
SMC1A_R699C_FWD	GTCAGCCACCACCTTACCAC
SMC1A_R699C_R	ACCTAGGCCAGGAATGTGTG
SMC1A_R699C_REV	ACCTAGGCCAGGAATGTGTG
SOS2_T654A_F	CCACTTTTCGTGGAAGTGGT
SOS2_T654A_R	CTGGTTGGACATATTCCTTGC
SPI1_R171S_FWD	GAGTGTGGGTGCGTTGTGTA
SPI1_R171S_R	CACCAGATGCTGTCCTTCAT
SRSF2_A62T_FWD	CGCCCAGTTGTTACTCAGGT
SRSF2_A62T_REV	CCACCTTGAGGGAGGTCATA
SRSF2_DEL99R_FWD	GACCGCTACACCAAGGAGTC
SRSF2_DEL99R_REV	CCTCAGCCCCGTTTACCT
SRSF2_T132I_FWD	ATGGCATCCATAGCGTCCT
SRSF2_T132I_REV	CAAGGTGGACAACCTGACCT
SRSF2_P95T_WT_REV	CTGTGGTGTGAGTCCGAGG
SRSF2_P95T_MUT_REV	CTGTGGTGTGAGTCCGAGT
SRSF2_P95T_FWD	GCTGAGGACGCTATGGATG
SUZ12_N277S_FWD	GCAAAATTGGAAACATGTGG
SUZ12_N277S_REV	TTGTGCAACAAATGTCTTTTCC
TEK_I718K_FWD	AAGGGCCTAGAGCCTGAAAC
TEK_I718K_REV	CCTATAGGGCTGCACGGTAA
TERC_E20K_FWD	GCTCCCTTTATAAGCCGACTC
TERC_E20K_REV	GCTGAAAGTCAGCGAGAAAA
TET2_A1605S_FWD	TTCTGGATCCACCAATCCAT
TET2_A1605S_REV	ATATGGGGAGCAGTTGTCCA
TET2_C1382Y_FWD	TGTCATTCCATTTTGTTTCTGG
TET2_C1382Y_REV	GCATGAGCACAGAAGTCCAA
TET2_C332STOP_FWD	TGTGATGCTGATGATGCTGA
TET2_C332STOP_REV	ATTGCTGCTGGAACCTGAAC
TET2_FS_FWD	ACATGCTCCATGAACAACCA
TET2_FS_FWD	CAAAATCAAGCGAGTTCGAG
TET2_FS_REV	GTGTGGTTATGCCACAGCTT
TET2_FS_REV	GGGTCTTGTTTCCTGCAAAA
TET2_FWD	ACCACCCAATCTGAGCAATC
TET2_H1912Y_FWD	CAATAGGAATCACCCCACCA
TET2_H1912Y_REV	AGGCTCCCGTTTCACTTTTT
TET2_Q803STOP_FWD	ACTTTTCCTCACCCCCAAAG
TET2_Q803STOP_REV	TGCACTTGATTTCATGGTCTG
TET2_R1261C_FWD	GTGAGGCTGCAGTGATTGTG
TET2_R1261C_REV	ACCAAAGATTGGGCTTTCCT
TET2_REV	TGTAAGCCTCCTTGGACACA
TET2_S1203R_FWD	TGCAAGTGACCCTTGTTTTG
TET2_S1203R_REV	ATTTCCTCAGCGTCTCGGTA
TET2_W1003_FWD	TTAAGGTGGAACCTGGATGC
TET2_W1003_REV	ACTGGCCCTGACATTTCAAC
TET2_Y1294C_FWD	AGGAGAACTTGCGCCTGTC
TET2_Y1294C_R	CAAACCTCTTTTGGGTCATCC
tet21216stop_fwd	TGCAAGTGACCCTTGTTTTG
tet21216stop_fwd	ATTTCCTCAGCGTCTCGGTA
tet2fs_fwd	TAGCCACACCCCAGCTTTAG
tet2fs_rev	TTCTGGATAAACGCCATGTG
TET3_FWD	CTCTGAAGGGTGGATTGTCC
TET3_P175A_FWD	GAAACCACCCAACTGCAACT
TET3_P175A_REV	GAGAGATCTGGGGCACCTCT
TET3_P512I_FWD	GACAGACCACCCAAGGAGAA
TET3_P512I_REV	AAGCCCTTCCAGGACAATCT
TET3_R577Q_FWD	CCTTCTCTTGCGCTATTTGC
TET3_R577Q_REV	GGGAGACATGTTTGCTGGTT
TET3_R899STOP_FWD	ATGTGTGACTGCCCCTCTCT
TET3_R899STOP_REV	ACTCTCCCACTTCCCACCTT

TET3_REV	ACTTGGAGTGGAAGCCATTG
tp53_fwd	CCAACTGGCCAAGACCTG
TP53_FWD	GGGAGTAGATGGAGCCTGGT
TP53_R141H_FWD	CAAGGGTGGTTGGGAGTAGA
TP53_R141H_REV	TTGCGGAGATTCTCTTCCTC
tp53_rev	CTTAACCCCTCCTCCCAGAG
TP53_REV	GCTTCTTGTCCTGCTTGCTT
TYK2_LOB_FWD	CAGGGAGGGTGAGTACCTGA
TYK2_LOB_REV	CCGTCAAAGCAGATCTCCAG
U2AF1_S34F_FWD	TGCTGCTGACATATTCCATGT
U2AF1_S34F_REV	AGTCGATCACCTGCCTCACT
WT1_CAST_FWD	GCCGAGGCTAGACCTTCTCT
WT1_CAST_REV	TCCAATCCCTCTCATCACAA
WT1_FWD	GGAGTGTGAATGGGAGTGGT
WT1_PES_FWD	GGAGTGTGAATGGGAGTGGT
WT1_PES_REV	TTATTGCAGCCTGGGTAAGC
WT1_R140STOP_FWD	GGAGTGTGAATGGGAGTGGT
WT1_R140STOP_REV	TTATTGCAGCCTGGGTAAGC
WT1_REV	TTATTGCAGCCTGGGTAAGC
WT1_Y201*_FWD	CGCCTTCACTGTCCACTTTT
WT1_Y201*_REV	CCGGCCTACTTACCCTGATT
XRCC5_V42I_F	GACGTGGGCTTTACCATGAG
XRCC5_V42I_R	AACAGAGGGGCTGGACACTA
ZRSR2_E133V_FWD	GACCCGAAGAAGAGCATCAG
ZRSR2_E133V_REV	ATGGGAAGAACAGCAGAGGA
ZRSR2_V304M_FWD	CAAGCAGCCCTTTCTCTGTT
ZRSR2_V304M_REV	AGGCAAAAAGCTATGCCTCA

Supplementary Table 13 | Primers and probes for allele specific PCR assays

Forward Primer Name	Forward Primer Seq.	Reverse Primer Name	Reverse Primer Seq.	Reporter 1 Name	Reporter 1 Sequence	Reporter 2 Name	Reporter 2 Sequence
ABCC2_V669A_F	GGACATTATGGCAGGCCAACTT	ABCC2_V669A_R	ATGGCTGATATCAAGGAGGATTTCC	ABCC2_V669A_V	CAGGGCCTATCACAGCC	ABCC2_V669A_M	AGGGCCTATCGCAGCC
ASXL1_FS_F	GGGAGGCCCGAGCAC	ASXL1_FS_R	GCAGTAGTTGTGTTCGCTGTAG	ASXL1_FS_V	CTGACGTACACTTTCCAG	ASXL1_FS_M	ATCTGACGTACTTTCCAG
ASXL1_q373stop_F	GCTGGGTTTGACCAAAGAAGAGT	ASXL1_q373stop_R	GGACACACAAGCCACTTTTGAT	ASXL1_q373stop_V	CCACGTTCTGCTGCAAT	ASXL1_q373stop_M	CCCACGTTCTACTGCAAT
BCLAF1_R107C_F	CCTGTCTGGAATAGAAGGCACTCT	BCLAF1_R107C_R	CTGGATCTTTGAGAAGAAACGGATCT	BCLAF1_R107C_V	AACGTGAACGACCTCG	BCLAF1_R107C_M	CGTGAACAACCTCG
CDC27_G88E_F	AGAATATGTTTACATGAACTGCTTTTTATATT[illegible]	CDC27_G88E_R	CATCATGGCTTTTCTGCTTATTAAACACT	CDC27_G88E_V	AAGATTTGTTCCCCTTCTGC	CDC27_G88E_M	AGATTTGTTCCTCTTCTGC
cux1_F	CTCCTCATCGCTTCCTGTCA	cux1_R	GATGGGCTCTGGGATCTTCTC	cux1_V	AGGGTGCCGCTGAGC	cux1_M	AGGGTGCCACTGAGC
DNMT3A_F	TGTGGGAGCCTCAATGTTACC	DNMT3A_R	CTTGCAGTTTTGGCACATTCCT	DNMT3A_V	AACACCCCCTCTTCGTT	DNMT3A_M	CACCCCCCCTTCGTT
dnmt3a_fs_F	GAATGAATGCTGTGGAAGAAAACCA	dnmt3a_fs_R	GGGCTCAGGCGTGGTA	dnmt3a_fs_V	CAGCCCTCCTGCTGTG	dnmt3a_fs_M	AGCCCTCCTTGCTGTG
DNMT3A_L548H_F	CGTTAGTGACAAGAGGGACATCTC	DNMT3A_L548H_R	TGGCCAAACCAAGGTTGCT	DNMT3A_L548H_V	ATACCTCGAGAAATC	DNMT3A_L548H_M	CTATACCTCGTGAAATC
DNMT3A_R882C_F	AGTCCACTATACTGACGTCTCCAA	DNMT3A_R882C_R	CGGCCCAGCAGTCTCT	DNMT3A_R882C_V	CCAAGCGGCTCATG	DNMT3A_R882C_M	CCAAGCAGCTCATG
DNMT3A_R882H_F	AGTCCACTATACTGACGTCTCCAA	DNMT3A_R882H_R	CGGCCCAGCAGTCTCT	DNMT3A_R882H_V	CATGAGCCACTTGGC	DNMT3A_R882H_M	ATGAGCCGCTTGGC
EZH2_R249stop_F	TGTTCAGAGAGAGCAAAGCTTACAC	EZH2_R249stop_R	CACTTACGATGTAGGAAGCAGTCAT	EZH2_R249stop_V	CTTTTCTGTAGGCGATGTT	EZH2_R249stop_M	CTTTTCTGTAGGTGATGTT
FANCA_G809D_F	GCTCCCAGAGGTGGATGTG	FANCA_G809D_R	GAGCGCAGGGACAGGAA	FANCA_G809D_V	CTGCACCTGGTGCTGG	FANCA_G809D_M	TGCACCTGATGCTGG
FLT3_D835V_F	GGGAAAGTGGTGAAGATATGTGACT	FLT3_D835V_R	ACATTGCCCCTGACAACATAGTT	FLT3_D835V_V	TTGGCTCGAGATATCAT	FLT3_D835V_M	TTGGCTCGAGTTATCAT
FLT3_M664I_F	CTGAAAGAGAGGCACTCATGTCA	FLT3_M664I_R	CCCCAGCAGGTTCACAATATTCTC	FLT3_M664I_V	ACTCAAGATGATGACCC	FLT3_M664I_M	AACTCAAGATAATGACCC
FLT3_S451F_F	ACCTCAAGTGCTCGCAGAAG	FLT3_S451F_R	GGTCCAAGATGGTAATGGGTATCC	FLT3_S451F_V	CGAGAAACAGGACGCCTG	FLT3_S451F_M	CGAGAAACAGAACGCCTG
flt3_tkd_F	GGGAAAGTGGTGAAGATATGTGACT	flt3_tkd_R	ACATTGCCCCTGACAACATAGTT	flt3_tkd_V	TTGGCTCGAGATATCAT	flt3_tkd_M	ATTGGCTCGATATATCAT
GATA2_N317S_F	CGGCACCGGCCACTA	GATA2_N317S_R	TGCCCATTCATCTTGTGGTAGAG	GATA2_N317S_V	CCTGTGCAATGCCTGT	GATA2_N317S_M	CTGTGCAGTGCCTGT
JAG1_T713M_F	CCAGGTGACAGTCAGTGTGAT	JAG1_T713M_R	GCATCCCCCTCATCATAGCA	JAG1_T713M_V	TTGCACGTGGCCTC	JAG1_T713M_M	TTGCACATGGCCTC
KRAS_G13D_F	AGGCCTGCTGAAAATGACTGAATAT	KRAS_G13D_R	GAATTAGCTGTATCGTCAAGGCACT	KRAS_G13D_V	CTTGCCTACGCCACCAG	KRAS_G13D_M	CTTGCCTACGTCACCAG
KRAS_Q61H_F	GATGGAGAAACCTGTCTCTTGGAT	KRAS_Q61H_R	CCTCATGTACTGGTCCCTCATTG	KRAS_Q61H_V	CACTGTACTCCTCTTGACCT	KRAS_Q61H_M	ACTGTACTCCTCGTGACCT
MYBL1_I624M_F	CTGCCCCTTCAAACTCTTCCA	MYBL1_I624M_R	AAGAAGCCCTGGTTGAGCAA	MYBL1_I624M_V	CTGTTGTCTTCTTTGATACCT	MYBL1_I624M_M	CTGTTGTCTTCTTTCATACCT
npm1_F	GATGTCTATGAAGTGTTGTGGTTCCTT	npm1_R	TTGTTTAAACTATTTTCTTAAAGAGACTTCCTCCA	npm1_V	CTGCCAGAGATCTT	npm1_M	CATGCAGAGATCTT
NPM1_type_D_F	GATGTCTATGAAGTGTTGTGGTTCCTT	NPM1_type_D_R	CAGAAATGAAATAAGACGGAAAATTTTTTAACAAATTGT	NPM1_type_D_V	CTCCACTGCCAGAGAT	NPM1_type_D_M	TGCCAGGCAGAGAT
NRAS_Q61H_F	GGTGAAACCTGTTTGTTGGACATAC	NRAS_Q61H_R	CCTGTCCTCATGTATTGGTCTCTCA	NRAS_Q61H_V	CACTGTACTCTTCTTGTCCAG	NRAS_Q61H_M	ACTGTACTCTTCATGTCCAG
OXCT1_R285Q_F	CAAAGCATACCTCAACCACGAATAATTTAT	OXCT1_R285Q_R	TCATCTCCAGGTTTAGCAGATTTGG	OXCT1_R285Q_V	TTTATCAATCCGGAAAGAG	OXCT1_R285Q_M	TTATCAATCCAGAAAGAG
PTPN11_F	GACTTATGTGACCGTGGTCTCTTTT	PTPN11_R	GGATCACCATCGTGTAGGACAAC	PTPN11_V	CCTGGTATGATCAACTAGAA	PTPN11_M	CTGGTATGATCAGCTAGAA
RALPBP1_FS_F	AGGAATTCCAGCGTTTACTCAAAGA	RALPBP1_FS_R	GCACAATGAGCCAAGAAATCAGAAG	RALPBP1_FS_V	CTGCCAGAATGTAACTAT	RALPBP1_FS_M	TGCCAGAATGTTAACTAT
RET_G733D_F	GAATTCCCTCGGAAGAACTTGGTT	RET_G733D_R	CGTTGCCTTGACCACTTTTCC	RET_G733D_V	TAGGAGAAGGCGAATTT	RET_G733D_M	AGGAGAAGACGAATTT
RUNX1_D66fs_F	CCGGGCGAGCTGGT	RUNX1_D66fs_R	GCACGGAGCAGAGGAAGTT	RUNX1_D66fs_V	CACCGACAGCCCC	RUNX1_D66fs_M	CGACCGACAGCCCC
S0S2_T654A_F	CTCCTTTTGCTTTTCTTCTTAGGTTTGAA	S0S2_T654A_R	GCCTTTCTCTATTGCCAATTTGTCT	S0S2_T654A_V	AGCCAGAACCTACTGACG	S0S2_T654A_M	CCAGAACCTGCTGACG
TET21003fs_F	AGCCACATGCCTGTATGCA	TET21003fs_R	GCAGGTGGATTCTCTTGCTTAGTTA	TET21003fs_V	AAAACATGGAAAAAGG	TET21003fs_M	AACAAAACATGTAAAAGG

Forward Primer Name	Forward Primer Seq.	Reverse Primer Name	Reverse Primer Seq.	Reporter 1 Name	Reporter 1 Sequence	Reporter 2 Name	Reporter 2 Sequence
SPI1_R171S_F	CCCGCAGGCAGCAAGA	SPI1_R171S_R	GAGCAGGTCCAACAGGAACT	SPI1_R171S_V	CAGGCGGATCTTC	SPI1_R171S_M	ACAGGCTGATCTTC
tet2_fs_F	AGTCACCTTCCAAATTACTAGATACTCCT	tet2_fs_R	GCACTTACCTACACATCTGCAAGA	tet2_fs_V	TTGGATACACCTGTCAAGAC	tet2_fs_M	TTGGATACACCTTCAAGAC
TET2_FS_F	TGGGACTGGAGGAAGTACAGAAT	TET2_FS_R	AAACCTGTATTTTGCATGCACTTGAT	TET2_FS_V	ATCGTAGAAATTCCCCTTATAG	TET2_FS_M	AATCGTAGAAATTCCCTTATAG
TET2_H1912Y_F	CTCCCTCGTCTTTTACCAGCATAAG	TET2_H1912Y_R	ACGGGCTTTTTCAGCCATTTTG	TET2_H1912Y_V	CAAGCCATGTTTTGGC	TET2_H1912Y_M	CAAGCCATATTTTGGC
TET2_R1216st_F	GTGAAGAGAAGCTACTGTGTTTGGT	TET2_R1216st_R	ACAATCACTGCAGCCTCACA	TET2_R1216st_V	CCAGCTCGCTCCCG	TET2_R1216st_M	CCAGCTCACTCCCG
TET2_y1294c_F	TGCCTCCTTCTCTTTTGGTTGTT	TET2_y1294c_R	TGCTTCTGGCAAACTTACATCCA	TET2_y1294c_V	CATGGAGCATGTACTACAA	TET2_y1294c_M	TGGAGCATGTGCTACAA
TP53_y181c_F	TGTGGAGTATTTGGATGACAGAAACA	TP53_y181c_R	AGACCCCAGTTGCAAACCA	TP53_y181c_V	TGGTGCCCTATGAGCC	TP53_y181c_M	TGGTGCCCTGTGAGCC
U2AF1_S34F_F	AATTGGAGCATGTCGTCATGGA	U2AF1_S34F_R	GCAAACAAACCTGGCTAAACGT	U2AF1_S34F_V	CAACCGAGAGCACC	U2AF1_S34F_M	CAACCGAAAGCACC
XRCC5_V42I_F	CCTGGTATAGAATCCCCATTTGAACA	XRCC5_V42I_R	GGTTACAAGCTCAGTGTCAATCTG	XRCC5_V42I_V	TGATAACCATGTTTGTACAGCG	XRCC5_V42I_M	ATAACCATGTTTATACAGCG
PTPN11_A72V_F	ACTGGTGATTACTATGACCTGTATGGA	PTPN11_A72V_R	GCCCGTGATGTTCCATGTAATACT	PTPN11_A72V_V	AAAGTGGCAAATTT	PTPN11_A72V_M	CAAAGTGACAAATTT
NRAS_Q61K_F	GGTGAAACCTGTTTGTTGGACATAC	NRAS_Q61K_R	CCTGTCCTCATGTATTGGTCTCTCA	NRAS_Q61K_V	CTGTACTCTTCTTGTCCAGC	NRAS_Q61K_M	CTGTACTCTTCTTTTCCAGC
NPM1_typeA_F	GATGTCTATGAAGTGTTGTGGTTCCTT	NPM1_typeA_R	CAGAAATGAAATAAGACGGAAAATTTTTTAACAAATTGT	NPM1_typeA_V	CTCCACTGCCAGAGAT	NPM1_typeA_M	ACTGCCAGACAGAGAT
IDH1_R132H_F	CTTGTGAGTGGATGGGTAAAACCTA	IDH1_R132H_R	CCAACATGACTTACTTGATCCCCATA	IDH1_R132H_V	CATCATAGGTCGTCATGC	IDH1_R132H_M	ATCATAGGTCATCATGC
RUNX1Fs_F	CCGGGCGAGCTGGT	RUNX1Fs_R	GCACGGAGCAGAGGAAGTT	RUNX1Fs_V	CACCGACAGCCCC	RUNX1Fs_M	CGACCGACAGCCCC
EZH2_C504G_F	CGTGCCCTTGTGTGATAGCA	EZH2_C504G_R	AAGCAACAAATACTTACACTCTGAACTACA	EZH2_C504G_V	AACTTTTCACAAAAATT	EZH2_C504G_M	ACTTTTCACCAAAATT
SF3B1_K666N_F	GCTGTGTGCAAAAGCAAGAAGTC	SF3B1_K666N_R	GCACAGCCCATAAGAATAGCTATCT	SF3B1_K666N_V	CACACTGGTATTAAGATTGTA	SF3B1_K666N_M	CACACTGGTATTAATATTGTA
JAK1_L1053V_F	GTGTTCTGCTTCCTTTCAAGGTATG	JAK1_L1053V_R	GACCAGACGTCAGAGGCAATATAAA	JAK1_L1053V_V	CTCCAGAATGTTTAATGCA	JAK1_L1053V_M	CCAGAATGTGTAATGCA
FLT3_D839E_F	GACTTTGGATTGGCTCGAGATATCA	FLT3_D839E_R	GGAAATAGCAGCCTCACATTGC	FLT3_D839E_V	AACATAGTTGGAATCACTC	FLT3_D839E_M	ACATAGTTGGATTCACTC
TET2_Q1654st_F	GGACAACTGCTCCCCATATCTG	TET2_Q1654st_R	GGGTCTTGGCTTGGATACCTATACA	TET2_Q1654st_V	CCATCGGCTGAGACTG	TET2_Q1654st_M	CATCGGCTAAGACTG
CTCF_R14C_F	CTTTGTGACCAGTGGAGAATTGG	CTCF_R14C_R	CATGGAACACTTGAATGGCTTCTC	CTCF_R14C_V	TGTGTTTGTAACGACGATGC	CTCF_R14C_M	TGTGTTTGTAACAACGATGC
TET2_L627fs_F	GCAAGCTTACACCCAGAAAACA	TET2_L627fs_R	GGGACTGCCCTTGATTCATTTCA	TET2_L627fs_V	TTGTGCTCCAGCTGTGT	TET2_L627fs_M	CTTGTGCTCCACTGTGT
FLT3_A680V	na	na	na	na	na	na	na
TET2_splice_F	AGTTCTCAGGGATGTCCTATTGCTA	TET2_splice_R	CCCAAGATTTAAGACCAAAGGCTTT	TET2_splice_V	ATCAAGTCACACTTACCCAC	TET2_splice_M	CAAGTCACACCTACCCAC
2015-027_ATM_F	TCTCAAGCAAATGATCAAGAAGTTGGA	2015-027_ATM_R	AATAACAGTAAAACACTAATCCAGCCAATAAAAAAA	2015-027_ATM_V	TGTGCAGCGGTTTGT	2015-027_ATM_M	TGTGCAGCAGTTTGT
2015-027_NSD1_F	TCACTGGCTTTTGATTCTGAACATTCT	2015-027_NSD1_R	GTCCTTTTTGGATTATCAGAGCTCTTTCT	2015-027_NSD1_V	AAGCCTTGCGCTAAAT	2015-027_NSD1_M	AAAGCCTTGTGCTAAAT
2015-027_ETV6_F	CTCAAGTGGGCTGAAAATGAGTTTT	2015-027_ETV6_R	GCAGGAGAGCTTTGCCATTC	2015-027_ETV6_V	CAGCAACACGTTTGAA	2015-027_ETV6_M	CAGCAACACATTTGAA
2015-027_setbp1_F	CACCACCTCAGTTCCACACA	2015-027_setbp1_R	CATGCTTGGCTTTATGCTTAGCT	2015-027_setbp1_V	ACTCCCACGTAAAGAT	2015-027_setbp1_M	AACTCCCACATAAAGAT
2015-027_dnmt3a_F	GTTCAGCAAAGTGAGGACCATTACT	2015-027_dnmt3a_R	GACAGGAAAATGCTGGTCTTTGC	2015-027_dnmt3a_V	CTTTATGGAGTTTGACCTCG	2015-027_dnmt3a_M	ATGGAGTCTGACCTCG
2015-027_nf1_F	TCTCAGGATAGTGCAGCAGGAT	2015-027_nf1_R	TCTGGGCTTGTCGGCAAAT	2015-027_nf1_V	CAGCGGAACCCCCCCG	2015-027_nf1_M	AGCGGAACCCCCCCG
2015-027_tp53l111r_F	GAAAACCTACCAGGGCAGCTA	2015-027_tp53l111r_R	AGACTTGGCTGTCCCAGAATG	2015-027_tp53l111r_V	CAAGAAGCCCAGACGGA	2015-027_tp53l111r_M	CAAGAAGCCCCGACGGA
2015-027_tp53l198r_F	AGCCAAAGAAGAAACCACTGGAT	2015-027_tp53l198r_R	CACTTGATAAGAGGTCCCAAGACTT	2015-027_tp53l198r_V	AGAATATTTCACCCTTCAGGTAC	2015-027_tp53l198r_M	TTCACCCGTCAGGTAC
2015-027_ctcf_F	GTCCGTTTCAGTGCAGTTTGTG	2015-027_ctcf_R	TCTCATGTGCCTTTTCAGCTTGT	2015-027_ctcf_V	ATGTGTCCCTGCTGGCAT	2015-027_ctcf_M	ATGTGTCCCTACTGGCAT

Supplementary Table 14 | Antibodies used for flow cytometry analyses

Antibody		Clone	Working dilution
PE-Cy™7 Mouse Anti-Human CD45	BD pharmingen	HI30	1:20
APC Mouse Anti-Human CD45	BD pharmingen	HI30	1:5
PE Mouse Anti-Human CD45	BD pharmingen	HI30	1:5
PE Mouse Anti-Human CD33	BD pharmingen	WM53	1:5
FITC Mouse Anti-Human CD33	BD pharmingen	HIM3-4	1:5
PerCP-Cy™5.5 Mouse Anti-Human CD33	BD pharmingen	P67.6	1:5
PE Mouse Anti-Human CD19	BD pharmingen	HIB19	1:5
FITC Mouse Anti-Human CD19	BD pharmingen	HIB19	1:5
PE Mouse Anti-Human CD3	BD pharmingen	UCHT1	1:5
FITC Mouse Anti-Human CD3	BD pharmingen	UCHT1	1:5
APC Mouse Anti-Human CD3	BD pharmingen	UCHT1	1:5
APC Rat anti-mouse CD45	Biolegend	30-F11	1:20
PerCP Rat anti-mouse CD45	Biolegend	30-F11	1:20
PE Mouse IgG1, κ Isotype Control	BD pharmingen	MOPC-21	1:5
PerCP-Cy™5.5 Mouse IgG1, κ Isotype Control	BD pharmingen	MOPC-21	1:5
PE-Cy™7 Mouse IgG1 κ Isotype Control	BD pharmingen	MOPC-21	1:20
FITC Mouse IgG1, κ Isotype Control	BD pharmingen	MOPC-21	1:5
APC-Cy™7 Rat Anti-Mouse CD45	BD pharmingen	30-F11	1:20
APC-Cy™7 Mouse IgG2b, κ Isotype Control	BD pharmingen	27-35	1:20
APC Rat IgG2b, κ Isotype Ctrl	Biolegend	RTK4530	1:20
PerCP Rat IgG2b, κ Isotype Ctrl	Biolegend	RTK4530	1:20
